# 2-Hetaryl-1,3-tropolones based on five-membered nitrogen heterocycles: synthesis, structure and properties

**DOI:** 10.3762/bjoc.11.236

**Published:** 2015-11-12

**Authors:** Yury A Sayapin, Inna O Tupaeva, Alexandra A Kolodina, Eugeny A Gusakov, Vitaly N Komissarov, Igor V Dorogan, Nadezhda I Makarova, Anatoly V Metelitsa, Valery V Tkachev, Sergey M Aldoshin, Vladimir I Minkin

**Affiliations:** 1 Southern Scientific Center of Russian Academy of Sciences, 141 Chekhov St., 344006 Rostov on Don, Russian Federation; 2 Institute of Physical and Organic Chemistry, Southern Federal University, 194/2 Stachka St., 344090 Rostov on Don, Russian Federation; 3 Institute of Problems of Chemical Physics of Russian Academy of Sciences, 1 Akad. Semjonov N.N. Ave., 142432 Chernogolovka, Moscow region, Russian Federation

**Keywords:** β-tropolones, fluorescence, intramolecular hydrogen bond, tautomerism, X-ray analysis

## Abstract

A series of derivatives of 2-hetaryl-1,3-tropolone (β-tropolone) was prepared by the acid-catalyzed reaction of 2-methylbenzoxazoles, 2-methylbenzothiazoles and 2,3,3-trimethylindoline with 3,4,5,6-tetrachloro-1,2-benzoquinone. The molecular structures of the three representative compounds were determined by X-ray crystallography. In crystal and (as shown by the DFT PBE0/6-311+G** calculations) in solution, 2-hetaryl-4,5,6,7-tetrachloro- and 2-hetaryl-5,6,7-trichloro-1,3-tropolones exist in the NH-tautomeric form with a strong resonance-assisted intramolecular N–H···O hydrogen bond. The mechanism of the formation of 1,3-tropolones in the reaction of methylene-active five-membered heterocycles with o-chloranil in acetic acid solution has been studied using density functional theory (DFT) methods. The reaction of 2-(2-benzoxa(thia)zolyl)-5,6,7-trichloro(4,5,6,7-tetrachloro)-1,3-tropolones with alcohols leads to the contraction of the seven-membered tropone ring with the formation of 2-(2-benzoxa(thia)zolyl)-6-alkoxycarbonylphenols. The molecular structure of 2-(2-ethoxycarbonyl-6-hydroxy-3,4,5-trichlorophenyl)benzoxazole has been determined by X-ray diffraction. 2-(2-Benzoxa(thia)zolyl)-6-alkoxycarbonylphenols display intense green fluorescence with anomalous Stokes shifts caused by the excited state intramolecular proton transfer (ESIPT) effects.

## Introduction

Tropolone derivatives represent one of the promising classes of compounds having a wide spectrum of biological activities: in particular, antitumor activity [1–2], barrier properties with respect to various pathogens, insects and microorganisms [3]. The natural compound hinokitiol (4-isopropyl-1,2-tropolone) isolated from *Chamacyparis taiwanensis* possesses antimicrobial and antifungal activity [4–6], pronounced insecticidal properties [7–8], and the capability of inhibiting the growth of plants [9]. It also exerts a cytotoxic effect on tumor cells [10] and serves as a potent inhibitor of catechol-*O*-methyltransferase [11], matrix metalloproteinases, carboxy-peptidase A, collagenase and thermolysin [12].

The biological activity of derivatives of 1,2-tropolone was the impetus for the rapid development of various methods for the synthesis and study of biological activity of these compounds. At the same time, the related molecular system of 1,3-tropolone has been insufficiently investigated. Therefore, the development of methods for the synthesis of 1,3-tropolones and the study of their properties is of considerable interest.

The parent compound was first obtained via decarboxylation of 3,5-dimethylcycloheptatrienecarboxylic acid to 3,5-dimethoxycycloheptatriene, bromination of the latter in chloroform to give β-methoxytropone and its subsequent demethylation [13]. The more general approach to β-tropolone was based on the multistep transformation of 3,4,5-trimethoxybenzoic acid [14–15]. An alternative method [16] involves the photooxygenation of cyclohepta-1,3,5-triene with singlet oxygen in carbon tetrachloride solution, methanolysis of the formed isomeric endoperoxides resulted in 1,2-dihydro-3-hydroxytropone and oxidation of the latter with chromium trioxide.

A convenient approach to the construction of seven-membered rings of 1,3-tropolone derivatives has been afforded by the acid-catalyzed reaction of *o*-quinones with methylene active compounds that proceeds with the expansion of the quinone six-membered ring [17–20]. The study of the reaction had been put forward by Schenk et al. [21] who reported on the preparation of a derivative of 1,2-tropolone **1** through condensation of 3,4,5,6-tetrachloro-1,2-benzoquinone (*o*-chloranil) with acetone. In the subsequent study of this reaction, the structure of its product analyzed with the use of two-dimensional NMR spectroscopy has been corrected and attributed to a derivative of 1,3-tropolone system **2** [17]. Later on this conclusion has been corroborated by the preparation of a series of 2-benzoyl-3-hydroxy-5,6,7-trichlorotropones **3** by the iron trichloride-catalyzed reaction of *o*-chloranil with acetophenones [22].

**Scheme 1:**
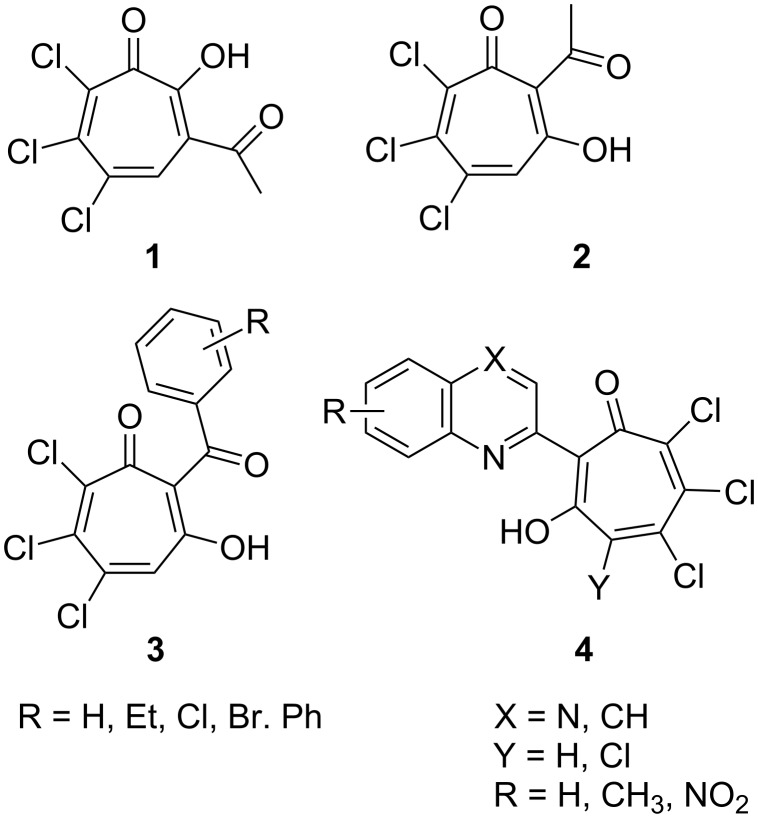
1,3-Tropolones **2**–**4** prepared by the reaction of o-chloranil with methylene active compounds.

The formation of the 1,3-tropolones by the proposed methods [21–22] is accompanied by dehydrochlorination of the dihydrotropone cycle. Our approach [23] to the construction of the 1,3-tropolone cycle enables to obtain additional reaction products with preservation of a functional group in the 4-position of the tropone ring. We have recently shown that coupling of 2-methylquinolines and 2-methylquinoxalines with *o*-chloranil gives rise to the formation of 2-(2-quinolyl)-4,5,6,7-tetrachloro-1,3-tropolones **4** (X = CH) [23] and 2-(2-quinoxalyl)-4,5,6,7-tetrachloro-1,3-tropolones [24], respectively (Scheme 1).

In the present work, we applied the methodology for constructing seven-membered 1,3-tropolone rings optimized in the course of the preparation of 2-quinolinyl-1,3-tropolones [23] for the synthesis of novel 2-hetaryl-1,3-tropolones obtained by the acid-catalyzed condensation of *o*-chloranil and 3,4,5,6-tetrachloro-1,2-benzoquinone with methylene-active five-membered heterocyclic compounds: 2-methylbenzoxazoles, 2-methylbenzothiazoles and 2,3,3-trimethylindoline. We have previously reported the synthesis of 2-(benzoxazolyl)-1,3-tropolones, 2-(benzothiazolyl)-1,3-tropolone, 2-(benzoxazolyl)-5,6,7-trichloro-1,3-tropolone, 2-(benzoxazolyl)-4,5,6,7-tetrachloro-1,3-tropolone, 2-(2-ethoxycarbonyl-3,4-dichloro-6-hydroxyphenyl)benzoxazole, 2-(2-ethoxycarbonyl-6-hydroxy-3,4,5-trichlorophenyl)benzoxazole, 2-(5-chlorobenzothiazolyl)-5,6,7-trichloro-1,3-tropolone and 2-(5-chlorobenzothiazolyl)-4,5,6,7-tetrachloro-1,3-tropolone [25–26], but no comprehensive study of the mechanism of the expansion of the *o*-quinone rings in this reaction has yet been undertaken. Therefore, one of the purposes of the present work was to gain insight into the major details of the mechanism of the formation of 5,6,7-trichloro-1,3-tropolones and 4,5,6,7-tetrachloro-1,3-tropolones in the reaction of methylene-active five-membered heterocycles with o-chloranil using density functional theory (DFT) quantum chemical methods.

Molecular and crystal structures of three representative 2-hetaryl-1,3-tropolones have been determined by X-ray and analyzed on the basis of DFT calculations with an emphasis on the tautomeric O–H···N 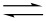 O···H–N relationships. It has been found that 2-(2-benzoxazolyl)- and 2-(2-benzothiazolyl)-4,5,6,7-tetrachloro-1,3-tropolones undergo contraction of their seven-membered rings under prolonged heating of their alcohol solutions with the formation of 2-(2-alkoxycarbonyl-6-hydroxyphenyl)benzazoles. Absorption and emission spectra of these compounds have been studied.

## Results and Discussion

In contrast with the cross-aldol reactions of *o*-chloranil with methylketones [17,21–22] always accompanied by elimination of one of the chlorine atoms from the seven-membered ring, the acid-catalyzed reaction between methylene-active nitrogen heterocycles and *о*-chloranil occurs, depends on the conditions of its performance, with or without inclusion of a stage of dehydrochlorination and leads to 5,6,7-trichloro- or 4,5,6,7-tetrachlorotropones, respectively [23].

**Scheme 2:**
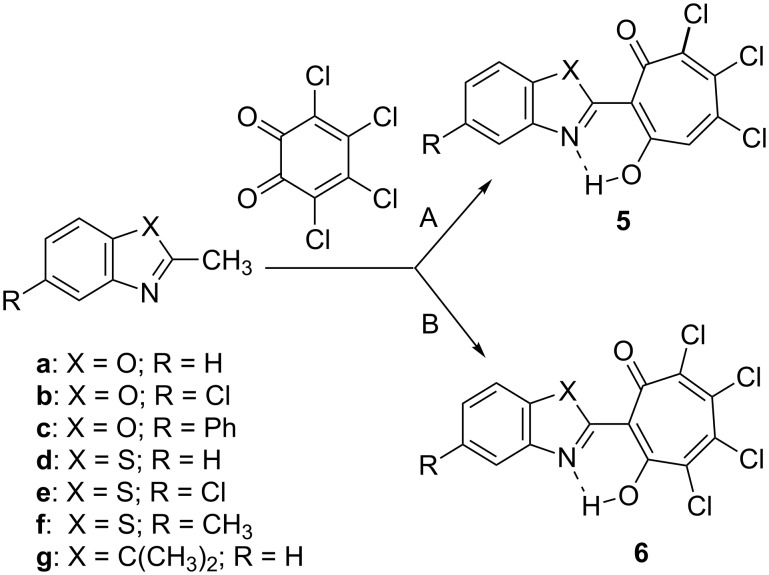
General scheme of the synthesis of 2-(2-hetaryl)-5,6,7-trichloro-1,3-tropolones **5** and 2-(2-hetaryl)-4,5,6,7-tetrachloro-1,3-tropolones **6**. Method A – heating under reflux of dioxane solution of the reactants. Method B – running reaction in acetic acid solution.

We have found that the short-term (10–40 min) heating under reflux of dioxane solutions of equimolar amounts of 2-methylbenzoxazole (2-methylbenzothiazole) and *о*-chloranil (method A) leads to the formation of trichloro-substituted 1,3-tropolones **5** as the main products of the reaction (Scheme 2), whereas only trace amounts of tetrachloro-1,3-tropolones **6** were isolated from the reaction mixture. 2,3,3-Trimethylindoline reacts with *о*-chloranil in a similar way. The attempts to extend this reaction to 2-methylbenzimidazole and 1,2-dimethyl-1*H*-benzo[*d*]imidazole failed, no expected 1,3-tropolones were formed under the above described conditions.

The ratio of the products was inversed when running the reaction under prolonged (3–4 days) heating of acetic acid solution of reactants at about 50 °С (method B). In this case, 4,5,6,7-tetrachloro-1,3-tropolones **6** were obtained in 10–35% yields and 5,6,7-trichloro-1,3-tropolones **5** were formed as the byproducts. The low yields of the desired products (10–35%) are caused by resinification of the reaction mixture and formation of difficult-to-isolate byproducts, the structure of which has not yet been established. The interaction of *o*-chloranil with derivatives of 2-methylquinoline by method A or B proceeds more smoothly giving rise to the corresponding trichloro- and tetrachloro-1,3-tropolones with yields in the range of 60–85% [23]. Among the five-membered nitrogen heterocycles entered into the reaction, the moderately high yields of 60% were achieved only in the case of the interaction of 2,3,3-trimethylindoline with *o*-chloranil using method A, as for the other compounds a further study aimed at optimization of the reaction conditions is still required.

**Scheme 3:**
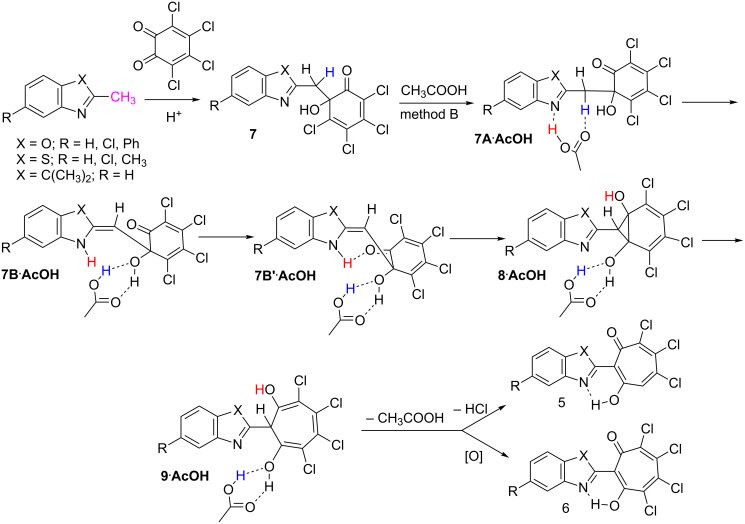
The mechanism for the formation of 5,6,7-trichloro-1,3-tropolones **5** and 4,5,6,7-tetrachloro-1,3-tropolones **6** in the reaction of methylene-active five-membered heterocycles with o-chloranil in acetic acid solution.

The mechanism of the reaction in acetic acid solution (method B) is similar to that considered previously for the reaction of *o*-chloranil with 2-methylquinolines [19] which has been studied by means of DFT PBE0/6-311+G** modelling (Scheme 3, Table 1).

**Table 1:** Total energies with zero-point energy correction (*E*_tot_ + ZPE, а.u.) and relative energies (Δ*E*, kcal/mol) of the intermediates and corresponding transition states (Ts) occurring along the reaction path of the formation of 1,3-tropolones **5**, and **6a**,**d**,**g** calculated using the PBE0/6-311+G** method in the gas phase.

Compound	(**a**) X = O, R = H		(**d**) X = S, R = H		(**g**) X = C(CH_3_)_2_, R = H	

	*E*_tot_ + ZPE	Δ*E*	*E*_tot_ + ZPE	Δ*E*	*E*_tot_ + ZPE	Δ*E*

**7A∙AcOH**	−2886.293775	0	−3209.198043	0	−2928.859452	0
**Ts(7A-7B)**	−2886.258805	21.9	−3209.164786	20.9	−2928.83261	16.8
**7B∙AcOH**	−2886.274487	12.1	−3209.181351	10.5	−2928.859918	−0.3
**7B'∙AcOH**	−2886.272219	13.5	−3209.178935	12.0	−2928.858531	0.6
**Ts(7B'-8)**	−2886.25786	22.5	−3209.164486	21.1	−2928.834626	15.6
**8∙AcOH**	−2886.272810	13.2	−3209.178937	12.0	−2928.842587	10.6
**Ts(8-9)**	−2886.268827	15.7	−3209.175382	14.2	−2928.838856	12.9
**9∙AcOH**	−2886.290779	1.9	−3209.196645	0.9	−2928.861403	−1.2

At the initial stage, the aldol condensation of the methylene-active heterocyclic compounds with *o*-chloranil affords the intermediate adducts, 6-(2-hetarylmethylene)-6-hydroxy-2,4-cyclohexadiene-1-ones **7**. Such type intermediates were isolated and structurally characterized in the reactions of 2-methylquinoline with 3,5-di-*tert*-butyl-*o*-quinone [19] and benzophenones with *o*-chloranil [22].

The addition of a methylene group of bismuthonium 2-oxoalkylides to a carbonyl carbon of an *o*-quinone ring is also considered to be the primary stage of the reaction leading to the formation of derivatives of 2-acyl-1,3-tropolones [27]. As shown previously [19] and in the present study as well, the essential ring-closing step preceded by the formation of the norcaradiene intermediates **8** must include the stage of the proton transfer from the methylene group of the intermediate adduct **7** to the nitrogen atom of the heterocycle. The double proton transfer resulting in the (**7A**∙**AcOH**) → (**7B**∙**AcOH**) isomerization is the limiting step of the whole reaction. The next reaction stage includes a minor structural transformation (**7B**∙**AcOH**) → (**7B'**∙**AcOH**), for which the transition state structure has not been located, most probably, because of the extreme flatness of the potential energy surface (PES) in this area. The subsequent proton transfer accompanied by cyclization leads to the norcaradiene derivatives **8**∙**AcOH**, which then rearrange to the dihydrotropolone derivatives **9**∙**AcOH**. In the presence of acetic acid with the twofold excess of o-chloranil, the main channel of the subsequent transformation is determined by the oxidation of **9** with the formation of 2-(2-hetaryl)-4,5,6,7-tetrachloro-1,3-tropolones **6** as the final products. The reaction mechanism in the dioxane solution (method A) differs from that above described only by the intramolecular character of the proton transfer stage.

The results of the calculations (Table 1) indicate that increase in the acceptor properties of the fragment X are manifested in the significant increase in the activation barrier of the double proton transfer stage (**7A**∙**AcOH**) → (**7B**∙**AcOH**) and in decrease in the activation barrier to the formation of norcaradiene derivatives (**7B'**∙**AcOH**) → (**8**∙**AcOH**). On the other hand, the low energy barrier transformation (**8**∙**AcOH**) → (**9**∙**AcOH**) does not reasonably depends on the nature of X.

It is worth noting that the structure of products of the reaction of 2-methylbenzazoles with 1,2-benzoquinones can be affected by various factors. Among the important ones, that determine the possibility of expansion of the *o*-quinone cycle and double proton transfer is basicity of the heterocyclic nitrogen. At the same time, this reaction can be fully inhibited by the presence of a heterocyclic component with additional reaction centers that drastically change the course of the reaction. It has been shown that the priority path of the reaction at the nitrogen atom unsubstituted 2-methylbenzimidazole with o-chloranil includes the nucleophilic addition of the quinone to the NH group [28], whereas the interaction of 3,5-di(*tert*-butyl)-1,2-benzoquinone with 2-methylbenzimidazole leads to the formation of polycyclic isoquinoline derivatives [29] and the reaction of 3,5-di(*tert*-butyl)-1,2-benzoquinone with 1,2,3-trimethyl-benzimidazolium salts gives rise a spirocyclic derivative of sterically hindered pyrocatechol [30].

**Figure 1:**
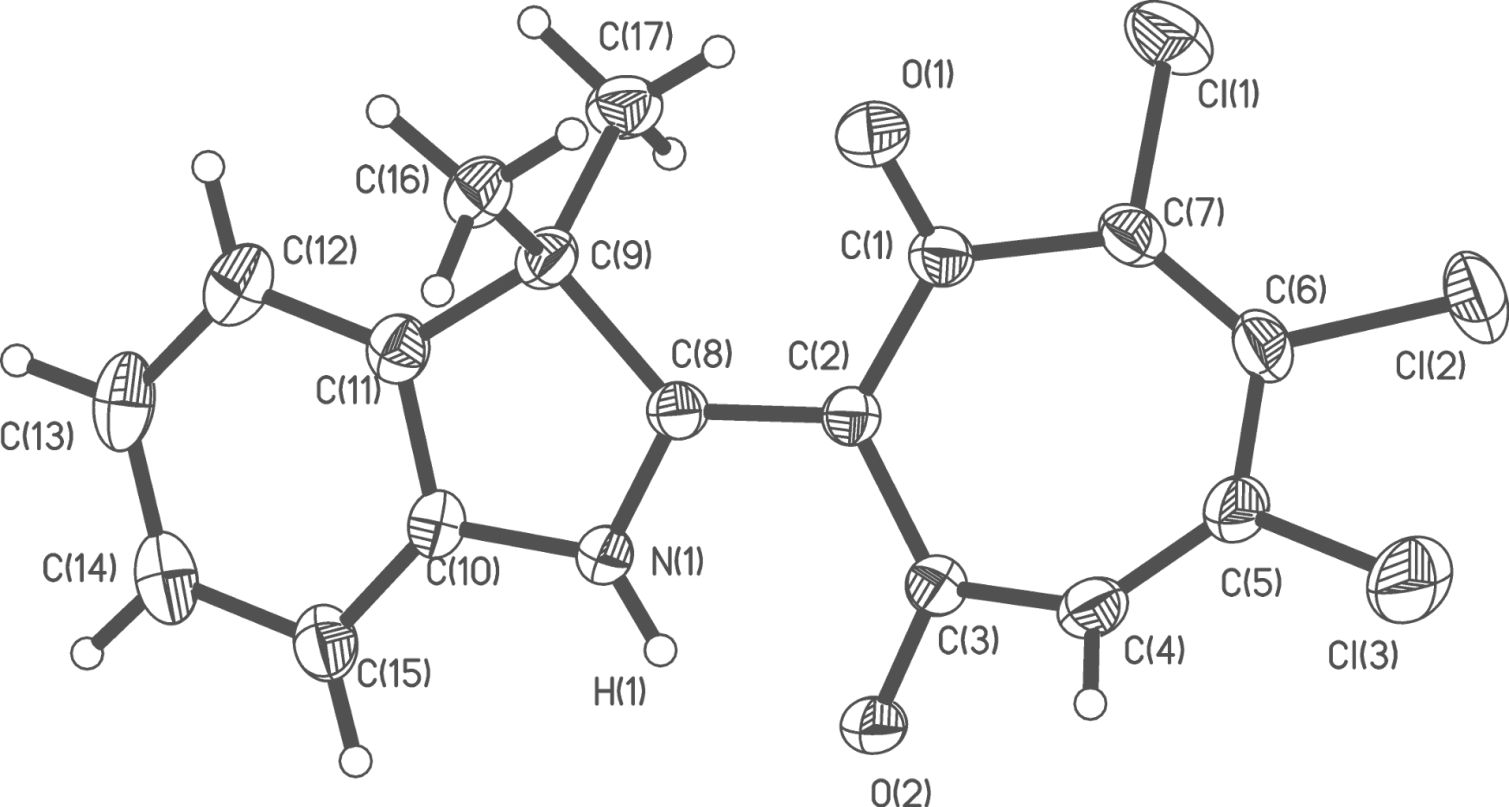
Molecular structure of 2-(3,3-dimethylindolyl)-5,6,7-trichloro-1,3-tropolone **5g**. Thermal ellipsoids are drawn on the 30% probability level. Selected bond lengths (Å): O(1)–C(1) 1.210(3), O(2)–C(3) 1.248(3), N(1)–C(8) 1.333(3), C(1)–C(2) 1.468(4), C(1)–C(7) 1.723(3), C(2)–C(3) 1.447(4), C(2)–C(8) 1.415(4), C(3)–C(4) 1.472(4), C(4)–C(5) 1.326(4), C(5)–C(6) 1.440(5), C(6)–C(7) 1.328(4), C(8)–C(9) 1.536(4); selected bond angles (^o^): O(1)–C(1)–C(2) 125.7(3), C(3)–C(2)–C(1) 119.7(2), C(3)–C(2)–C(8) 118.4(2), O(2)–C(3)–C(2) 123.5(2), N(1)–C(8)–C(2) 120.2(2).

**Figure 2:**
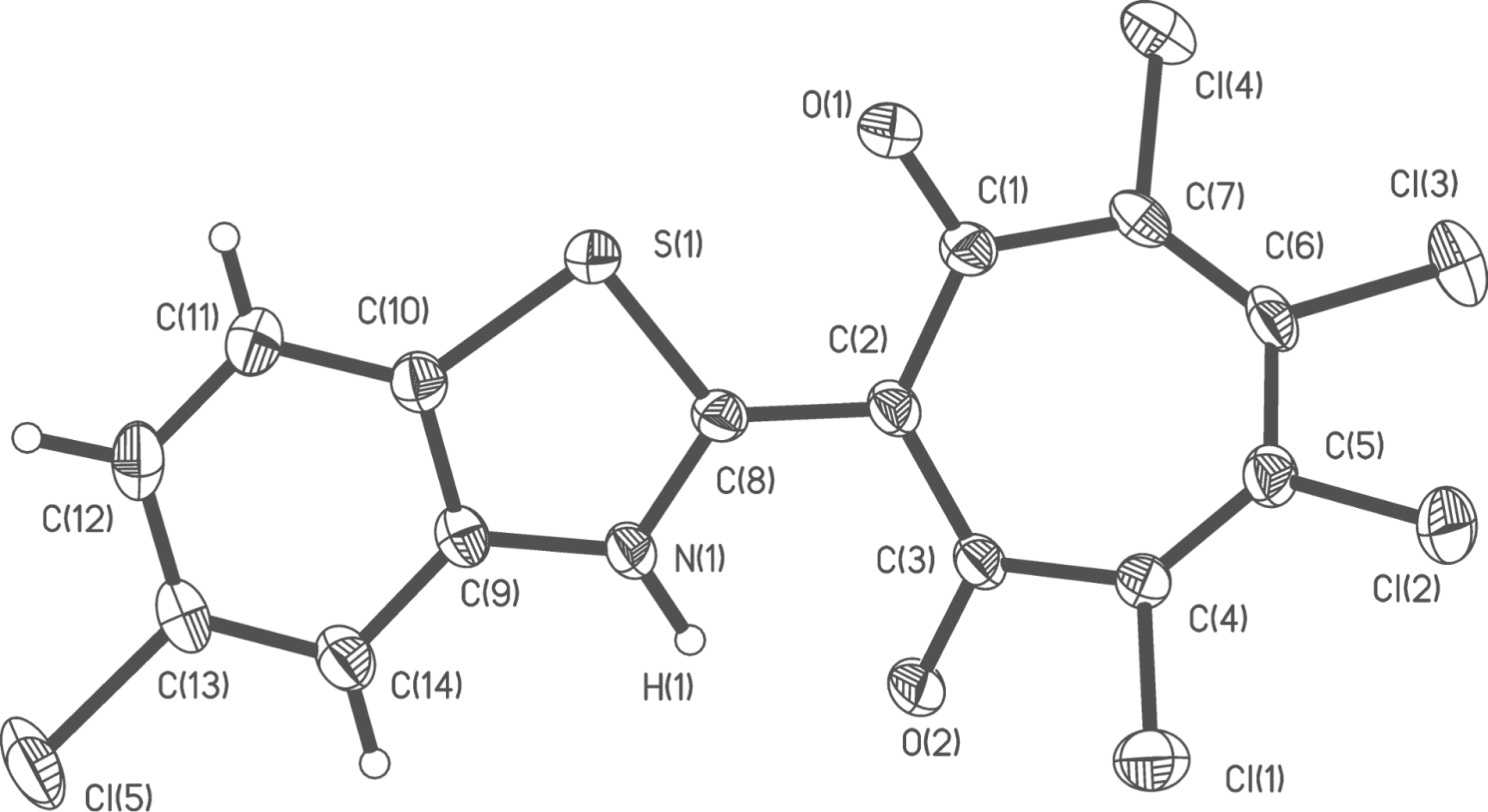
Molecular structure of 2-(5-chlorobenzothiazolyl)-4,5,6,7-tetrachloro-1,3-tropolone **6e**. Thermal ellipsoids are drawn on the 30% probability level. Selected bond lengths (Å): O(1)–C(1) 1.229(3), O(2)–C(3) 1.230(3), N(1)–C(8) 1.346(3), C(1)–C(2) 1.444(4), C(1)–C(7) 1.503(4), C(2)–C(3) 1.433(4), C(2)–C(8) 1.419(4), C(3)–C(4) 1.499(4), C(4)–C(5) 1.339(4), C(5)–C(6) 1.455(4), C(6)–C(7) 1.345(4), S(1)–C(8) 1.734(3); selected bond angles (^o^): O(1)–C(1)–C(2) 122.5(3), C(3)–C(2)–C(1) 126.2(2), C(3)–C(2)–C(8) 116.8(2), O(2)–C(3)–C(2) 123.4(3), N(1)–C(8)–C(2) 124.1(3). In the elementary unit cell of compound **6e** containing four molecules of the compound is also present a molecule of benzene located in the center of symmetry (0,0,1/2).

The structures of the prepared 2-hetaryl-1,3-tropolones have been characterized by ^1^H NMR, IR spectroscopy and mass spectrometry and also by the X-ray diffraction studies of the two compounds **5g** and **6e**, representing trichloro- and tetrachloro derivatives of the prepared tropolones. The overall views of the molecules are shown in Figure 1 and Figure 2. Unfortunately, we were unable to obtain crystals of 2-(benzoxazol-2-yl)-1,3-tropolones suitable for X-ray analysis.

In the independent parts of the elementary unit cells of **5g** and **6e** two molecules are located slightly differing in their structural details (Figures S1 and S2 in Supporting Information File 1). The geometric features of compounds **5g** and **6e** are similar to those of their 2-quinolyl congeners, 2-(2-quinolyl)-5,6,7-trichloro-1,3-tropolones and 2-(2-quinolyl)-4,5,6,7-tetrachloro-1,3-tropolones [23]. In all these compounds, the seven-membered rings are acoplanar and have bath conformations folded along the C(2)–C(7) and C(3)–C(6) lines by 140–150°.

**Scheme 4:**
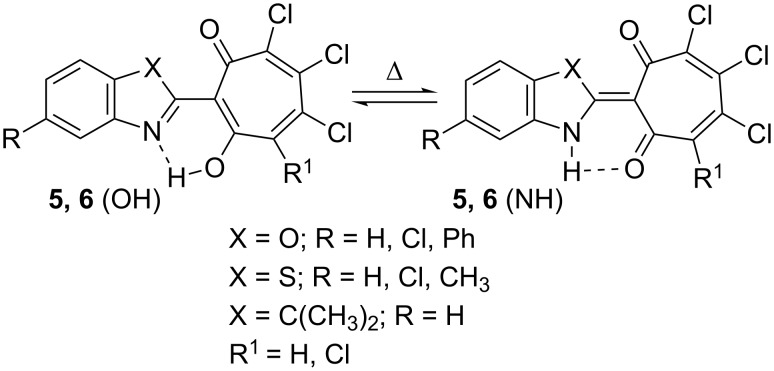
The fast prototropic N–H···O 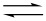 N···H–O equilibrium in solutions of 2-hetaryl-5,6,7-trichloro- and 4,5,6,7-tetrachloro-1,3-tropolones.

As in the previously studied 2-hetaryl-1,3-tropolones [19–20,23] the O···N distances in **5g** and **6e** are about 0.5 Å shorter than the corresponding van der Waals contact. This geometric feature points to the very strong intramolecular N-H···O hydrogen bond belonging to the type of the so-called resonance assisted hydrogen bonds [31]. Correspondingly, the protons in hydrogen bridges of **5g** and **6e** are characterized by very high downfield chemical shifts in the ^1^Н NMR spectra, although smaller than those (17–19 ppm) recorded for the 2-quinolinyl analogues. For all compounds under study these proton signals are significantly broadened because of the rapid N–H 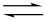 H–O exchanges depicted in Scheme 4.

In crystal, the proton within the N–H···O 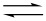 N···H–O bridge resides on the imine nitrogen of the 2-hetaryl groups of compounds **5g** and **6e**. Theoretical estimation of relative stabilities of the tautomeric NH and OH forms of the representative derivatives of 1,3-tropolones **5** and **6** in the gas phase and in a polar solvent (DMSO, Table 2) revealed that the decrease in the electronegativity of the fragment X in the row **6a**, **6d**, **6g** (X = O, S, C(CH_3_)_2_) shifts the chemical equilibrium toward the NH isomer. The same effect exerts an additional acceptor substituent (Cl) in the tropolone moiety. By contrast, the same substituent in the heterocyclic part of the molecule reduces the stabilization of the NH form. The polar environment additionally stabilizes the NH isomer relative the OH counterpart.

**Table 2:** Total energies with zero-point energy correction (*E***_tot_** + ZPE, а.u.) and relative energies (Δ*E*, kcal/mol) of the NH and OH tautomeric forms of compounds **5** and **6** calculated using the PBE0/6-311+G** method in the gas phase and in DMSO solution.

Compound	*E*_tot_ + ZPE(gas)	Δ*E*(gas)	*E*_tot_ + ZPE(sol)	Δ*E*(sol)

**6a(NH)**	−2656.243267	0	−2656.253163	0
**6a (OH)**	−2656.244467	−0.8	−2656.250511	2.3
**6d (NH)**	−2979.159003	0	−2979.166299	0
**6d (OH)**	−2979.156079	1.8	−2979.161255	3.8
**6e (NH)**	−3438.62948	0	−3438.63690	0
**6e (OH)**	−3438.627326	1.4	−3438.63255	3.4
**6g (NH)**	−2698.822842	0	−2698.82963	0
**6g (OH)**^a^	−2698.81666	3.9	–	–
**5g (NH)**	−2239.360137	0	−2239.367005	0
**5g (OH)**	−2239.356401	2.3	−2239.361745	3.5

^a^Isomer **6g** (OH) exist only in the gas phase.

The calculations reproduce sufficiently well the experimentally determined geometries of compounds **5g** and **6e** as well as their main structural features such as the very short O···N distances and the folding of the seven-membered rings (see Supporting Information File 1).

We have observed that long-term (6–8 h) heating under reflux of solutions of 2-hetaryl-1,3-tropolones **5**, **6а** and **5е** in methanol, ethanol or isopropanol leads to the contraction of the tropolone ring with the formation of 2-(2-alkoxycarbonyl-6-hydroxyphenyl)benzoxa(thia)zoles **11a–g**. Contraction of the tropone ring of 2-acyl-5,6,7-trichloro-1,3-tropolones under the action of alcohols [17,32] or hydrazines [32] has been previously reported.

The suggested primary step of these reactions is a nucleophilic addition to the carbonyl carbon of an acyl group. In our case (compounds **5** and **6**), the reaction most probably starts with an addition of an alcohol molecule to the most electrophilic center of the seven-membered ring and follows by elimination of hydrogen chloride.

**Scheme 5:**
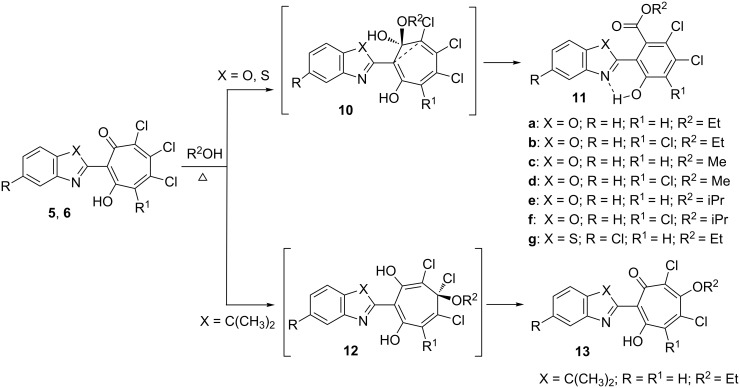
Two reaction paths for coupling 2-hetaryl-1,3-tropolones **5** and **6** with alcohols.

**Figure 3:**
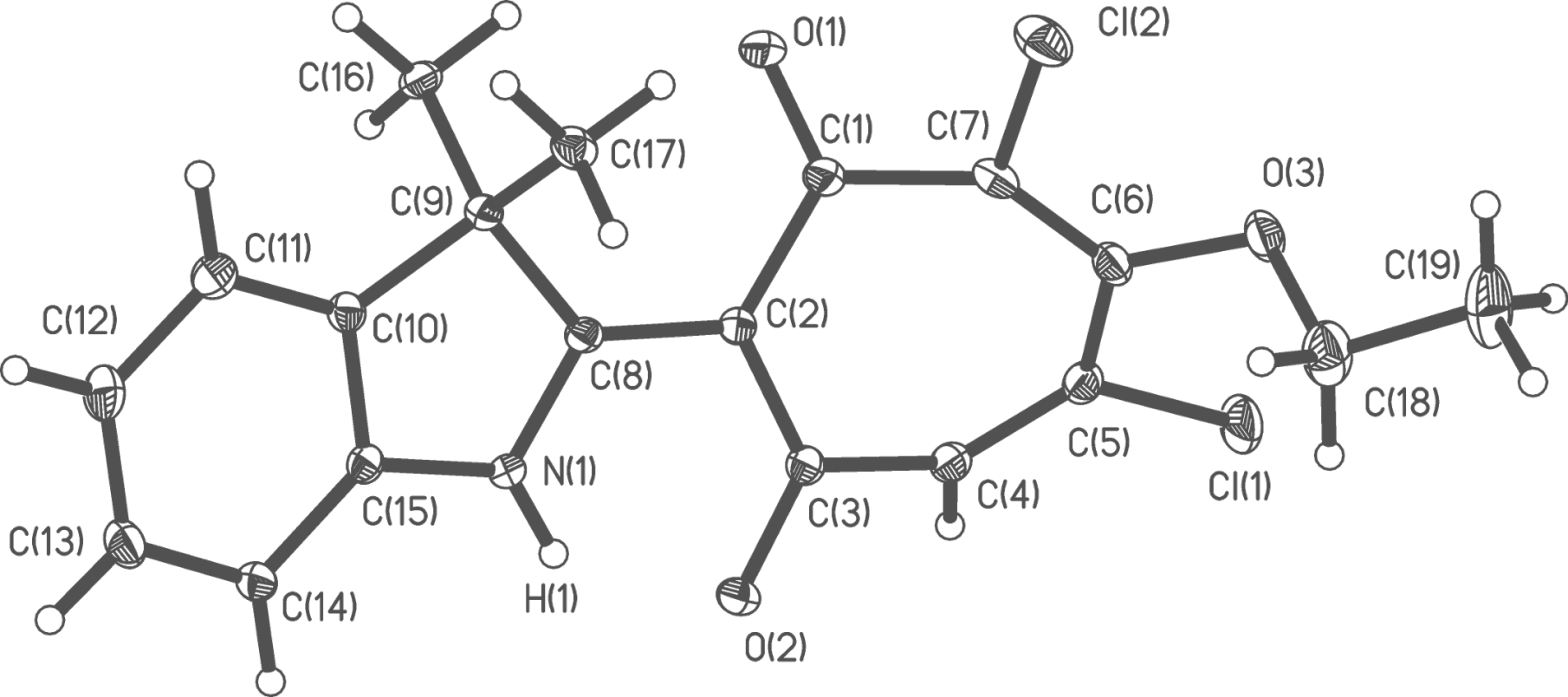
Molecular structure of 2-(3,3-dimethylindolyl)-5,7-dichloro-6-ethoxy-1,3-tropolone **13**. Selected bond lengths (Å): O(1)–C(1) 1.217(2), O(2)–C(3) 1.2563(19), N(1)–C(8) 1.334(2), C(1)–C(2) 1.471(2), C(1)–C(7) 1.508(2), C(2)–C(3) 1.444(2), C(2)–C(8) 1.422(2), C(3)–C(4) 1.475(2), C(4)–C(5) 1.335(2), C(5)–C(6) 1.461(2), C(6)–C(7) 1.343(2), C(8)–C(9) 1.546(2); selected bond angles (^o^) O(1)–C(1)–C(2) 123.71(16), C(3)–C(2)–C(1) 120.98(15), C(3)–C(2)–C(8) 118.71(14), O(2)–C(3)–C(2) 123.16(15), N(1)–C(8)–C(2) 120.27(14), N(1)–H(2)–O(2)= 136.5(9).

A nucleophilic substitution of a chlorine atom in the tropolone ring represents a competitive and in the case of **5g** energy preferable path of the reaction. A tentative mechanism for the reaction is proposed in Scheme 5. The molecular structure of the obtained 2-(3,3-dimethylindolyl)-1,3-tropolone **13** is shown in Figure 3.

**Figure 4:**
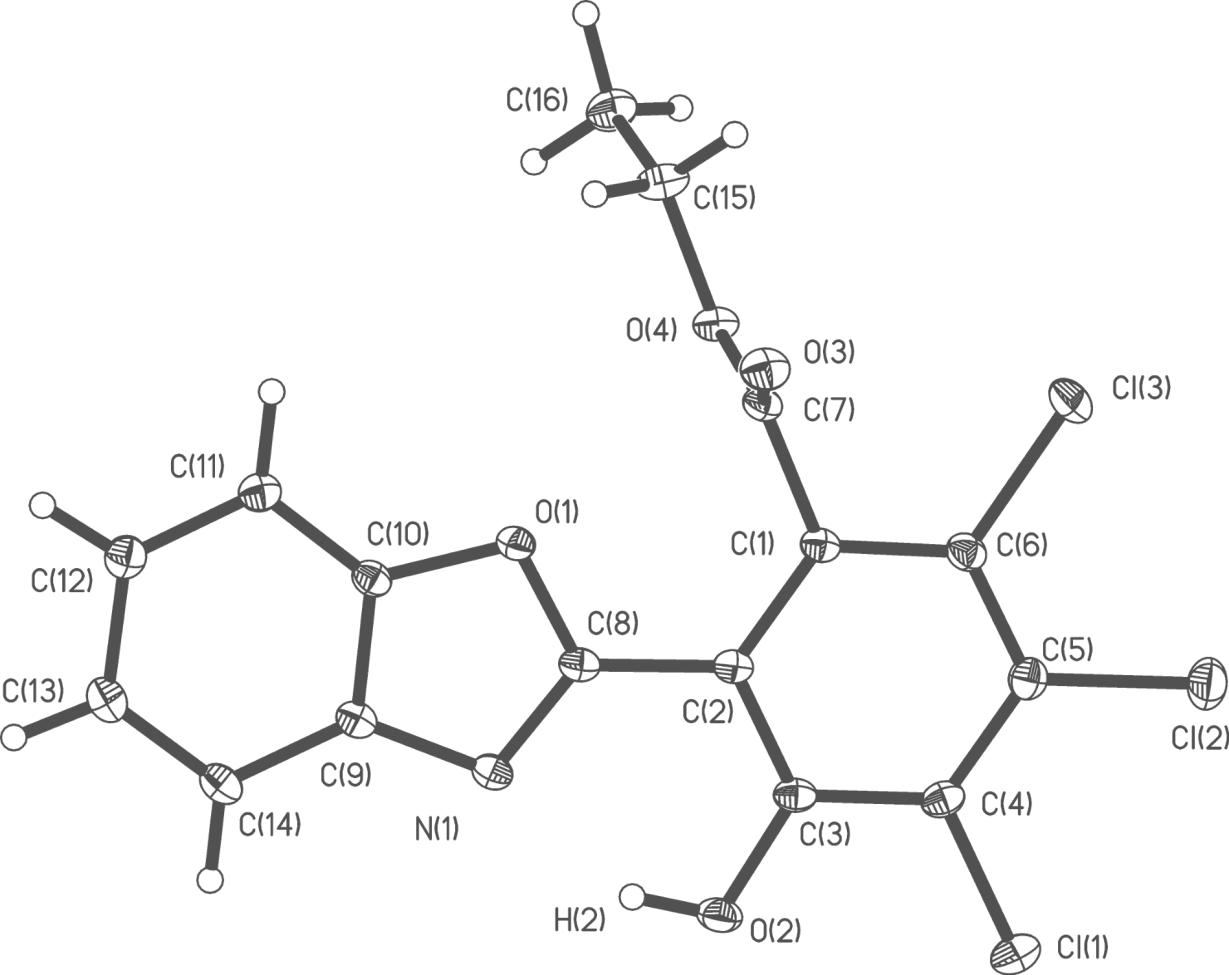
Molecular structure of 2-(2-ethoxycarbonyl-6-hydroxy-3,4,5-trichlorophenyl)benzoxazole **11b**. Selected bond lengths (Å): C(1)–C(2) 1.4071(13), C(2)–C(3) 1.4130(13), C(2)–C(8) 1.4511(14), N(1)–C(8) 1.3084(12), O(1)–C(8) 1.3621(12), O(2)–C(3) 1.3397(12), O(2)–H(2) 0.845(17); selected bond angles (^o^) N(1)–C(8)–O(1) 114.99(9), O(1)–C(8)–C(2) 119.76(8), N(1)–C(8)–C(2) 125.24(9), C(3)–C(2)–C(8) 117.85(8), C(1)–C(2)–C(8) 122.45(9).

As is the case of other studied 2-hetaryl-1,3-tropolones, compound **13** exists in the (NH) ground state tautomeric form with a very strong intramolecular N–H···O hydrogen bond that closes up the cycle Н(1)–N(1)–С(8)–С(2)–С(3)–О(2). The N(1)···О(2) distance (2.514 Å) is 0.5 Å shorter than the corresponding van der Waals contact angle. The molecular structure of a representative 2-(2-ethoxycarbonyl-6-hydroxy-3,4,5-trichlorophenyl)benzoxazole **11b** is shown in Figure 4.

All atoms of the benzoxazolyl fragment of **11b** are located in a single plane with the accuracy of 0.005 Å. A common plane (with the accuracy of 0.02 Å) is formed by С(1), С(2), С(3), С(4), С(5), C(7), О(2) and two chlorine atoms Cl(1) and Cl(2). The torsion angle N(1)–С(8)–С(2)–С(3) is equal to 1.5°. The intramolecular hydrogen bond in the cycle N(1)–С(8)–С(2)–С(3)–О(2)–Н(2) is characterized by the distances N(1)···О(2) = 2.599(4), N(1)···Н(2) = 1.830(6) Å and the angle N(1)–H(2)–O(2) of 150.4(9)°.

The proton signals of the hydroxy groups of **11** (R^1^ = H) and **11** (R^1^ = Cl) (Scheme 4) appearing in chloroform solution at 12.4 and 13.2 ppm are shifted towards stronger fields compared to the OH proton signals of the initial 1,3-tropolones **5** and **6** by about 3 and 2 ppm, respectively.

**Figure 5:**
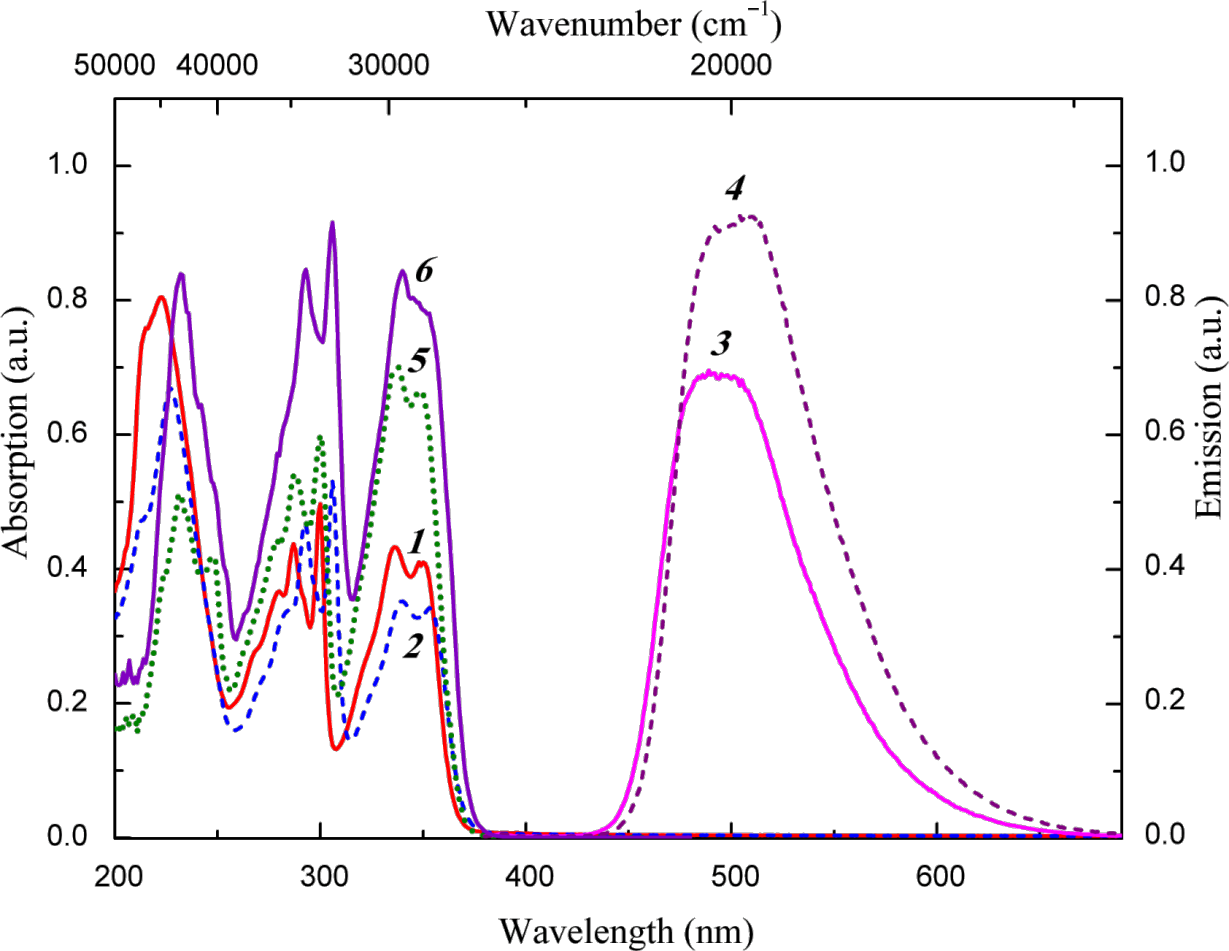
Electronic absorption (1, 2), fluorescence emission (λ_exc_ = 350 nm) (3, 4) and fluorescence excitation [λ_obs_ = 495 nm (5), λ_obs_ = 510 nm (6)] spectra of **11c** (1, 3, 5) and **11d** (2, 4, 6) in heptane solution (*c* = 2∙10^−5^ mol × L^−1^, l = 1 cm) at 293 K.

Compounds **11a–f** belong to the extensively studied class of 2-(2-hydroxyphenyl)benzazoles which has attracted much attention due to the applications in various molecular probes and luminescent materials because of the remarkable photophysical properties of the ESIPT (excited state intramolecular proton transfer) chromophores [33–34]. UV-irradiation of solutions of these compounds in nonpolar solvents results in the subpicosecond OH → NH ESIPT processes manifested by the fluorescence with anomalous Stokes shifts (ASS) in the spectral region around 500 nm. Accordingly, UV-illumination of heptane solutions of compounds **11a–f** excite their intense green fluorescence with maxima at 494 and 507 nm and large ASS (Δν_St_) of 8493 and 8605 cm^−1^, respectively (Figure 5, Table 3, Figure S7 (Supporting Information File 1)).

**Table 3:** Optical properties of 2-(2-alkoxycarbonyl-6-hydroxyphenyl)benzoxazoles **11a–f** in heptane solution at 293 K. Ф – Quantum yield of the fluorescence.

Compound	Absorption λ_max_ (nm)/ε (10^3^ M^−1^ сm^−1^)	Fluorescence		Stokes shift Δν_St_ (cm^−1^)

		Excitation λ_max_ (nm)	Emission λ_max_ (nm)/ Ф	

**11a**	348/22.25	348	494/0.16	8493
	336/23.35	337		

**11b**	353/17.15	353	507/0.19	8605
	339/17.70	339		

**11c**	348/20.55	348	494/0.18	8493
	336/21.65	336		

**11d**	353/17.15	353	507/0.22	8605
	339/17.16	340		

**11e**	348/19.40	348	494/0.15	8493
	336/20.35	336		

**11f**	353/19.30	353	507/0.20	8605
	339/20.10	339		

The electronic absorption spectra of compounds **11a–f** contain similar in form and position structured long-wavelength absorption bands with two maxima in the region of 336–353 nm (Figure 5, Table 3, Figure S8 (Supporting Information File 1)), the positions of which are relatively slightly affected by the substituents R^1^ and R^2^.

The most distinct is the effect exerted by the electron-withdrawing substituents (Cl, COOR) in the 2-hydroxyphenyl ring of compounds **11a–f** which is manifested in the batochromic (Δλ = 15–19 nm) shift of the longest wavelength absorption band as compared with the parent 2-(2-hydroxyphenyl)benzoxazole (cyclohexane, λ_max_ = 321, 334 nm [35]).

The excitation spectra of ASS-luminescence correspond to the absorption spectra of the hydroxy form of compounds **11a–f**, which indicates that irradiation of solutions of **11a–f** in heptane at 293 K initiates the ESIPT O−H···N → O···H−N responsible for the ASS-fluorescence.

It should be noted that the quantum yields of the ASS-fluorescence of compounds **11a,c,e** (R^1^ = H) (0.15–0.18) and **11b,d,f** (R^1^ = Cl) (0.19–0.22) are significantly higher than those displayed by the parent 2-(2-hydroxyphenyl)benzoxazole (0.02) and 2-(2-hydroxyphenyl)benzothiazole (0.005) [34].

## Conclusion

The ring expansion of the 3,4,5,6-tetrachloro-1,2-benzoquinones occuring under coupling with 2-methylbenzoxazoles, 2-methylbenzothiazoles and 2,3,3-trimethylindolines depending on the conditions leads to 2-hetaryl-5,6,7-trichloro-1,3-tropolones **5** (short-term heating under reflux of reactants in dioxane solution) or 2-hetaryl-4,5,6,7-tetrachloro-1,3-tropolones **6** (long-term heating of acetic acid solution at 50 °C). X-ray determinations of molecular structures of three compounds of these series as well as quantum chemical DFT PBE0/6-311+G** calculations point to energy preference of the NH-tautomeric form of the obtained 1,3-tropolones with the very strong intramolecular N–H···O bond closing up the conjugate six-membered ring.

The theoretically predicted activation energy barrier of the limiting step of the formation of 1,3-tropolone derivatives in the reaction of methylene-active five-membered heterocycles with o-chloranil in acetic acid solution strongly depends on the properties of the fragment X in the heterocyclic compounds. The larger the electronegativity of X, the higher the energy barrier to the limiting step of the proton transfer.

The reaction of 2-hetaryl-4,5,6,7-tri(tetra)chloro-1,3-tropolones **5** and **6** with alcohols proceeds through two reaction paths giving rise to either the products of nucleophilic substitution of a chlorine in the tropolone ring or to the contraction of the seven-membered ring with the formation of derivatives of 2-(hydroxyaryl)benzoxa(thia)zole **11***.* The 2-(2-alkoxycarbonyl-6-hydroxyphenyl)benzoxazoles **11** possess intense ASS-fluorescence with maxima at about 500 nm and quantum efficiencies in the range 0.15–0.22 (in heptane).

## Supporting Information

File 1 Experimental section, crystallographic data for compounds **5g**, **6e**, **11b**, **13**; optimized geometries of the intermediates and transition states involved in the routes of the formation of 1,3-tropolones **5**, **6a**,**d**,**g**; calculated geometries of the compounds **6a**, **6d**, **6e**, **6g** and **5g** in their OH and NH tautomeric forms in the gas phase; absorption and fluorescence spectra of compounds **11a**–**f** in heptane solution.

File 2 Copies of ^1^H and ^13^C NMR spectra of **5a-g**, **6a-g**, **11a-g**, **13**.
