# Supplementary material for: 2-Hetaryl-1,3-tropolones based on five-membered nitrogen heterocycles: synthesis, structure and properties
Source: Beilstein J Org Chem. 2015 Nov 12;11:2179–88. doi: 10.3762/bjoc.11.236 (PMC4661002; doi:10.3762/bjoc.11.236)
Supplement: File 1 — Experimental section, crystallographic data for compounds 5g, 6e, 11b, 13; optimized geometries of the intermediates and transition states involved in the routes of the formation of 1,3-tropolones 5, 6a,d,g; calculated geometries of the compounds 6a, 6d, 6e, 6g and 5g in their OH and NH tautomeric forms in the gas phase; absorption and fluorescence spectra of compounds 11a–f in heptane solution. [file Beilstein_J_Org_Chem-11-2179-s001.pdf]

Supporting Information File 1

for

2-Hetaryl-1,3-tropolones based on five-membered nitrogen heterocycles: synthesis, structure and properties

Yury A. Sayapin[1,2], Inna O. Tupaeva[2], Alexandra A. Kolodina[2], Eugeny A. Gusakov[2], Vitaly N. Komissarov[2], Igor V. Dorogan[2*], Nadezhda I. Makarova[2], Anatoly V. Metelitsa[2], Valery V. Tkachev[3], Sergey M. Aldoshin[3] and Vladimir I. Minkin[1,2].

Address: [1]Southern Scientific Center of Russian Academy of Sciences, 141 Chekhov St., 344006 Rostov on Don, Russian Federation, [2]Institute of Physical and Organic Chemistry, Southern Federal University, 194/2 Stachka St., 344090 Rostov on Don, Russian Federation and [3]Institute of Problems of Chemical Physics of Russian Academy of Sciences, 1 Akad. Semjonov N.N. Ave., 142432 Chernogolovka, Moscow region, Russian Federation

Email: Igor V. Dorogan - id@ipoc.sfedu.ru

* Corresponding author

Experimental section, crystallographic data for compounds 5g, 6e, 11b, 13; optimized geometries of the intermediates and transition states involved in the routes of the formation of 1,3-tropolones 5, 6a,d,g; calculated geometries of the compounds 6a, 6d, 6e, 6g and 5g in their OH and NH tautomeric forms in the gas phase; absorption and fluorescence spectra of compounds 11a–f in heptane solution.

Table of contents

1. Materials and methods……………………………………………….……….………S2–S3

2. Experimental section…………………………………………………………………..S3–S8

3. Principal experimental data of the X-ray diffraction analyses……………..………...S9–S10

4. Crystallographic data for **5g**, **6e**, **11b** and **13**....…………..........………....…………S11–S16

5. Calcululated geometries of the compounds **6a**, **6d**, **6e**, **6g** and **5g** in their OH and NH tautomeric forms in the gas phase...S17–S27
6. Calculated geometries of the intermediates and transition states involved in the routes of the formation of 1,3-tropolones **5,6a,d,g**...S28–S54
7. Fluorescence emission, fluorescence excitation and electronic absorption spectra spectra of compounds **11a-f** in heptane S55
8. References S56

Materials and methods

General procedure: The ^{1}H, ^{13}C NMR spectra were recorded on a VarianUnity-300 (300 MHz), «Bruker DPX-250» (250 MHz) and «Bruker AVANCE 600» (600 MHz) spectrometers. The IR spectra were measured on a Varian 3100FT-IR Excalibur Series instrument in the ATR (attenuated total reflectance) mode. The high resolution mass spectra (electrospray ionization) were obtained on a Bruker microTOF II instrument (capillary voltage 4500 V for positive ions or 3200 V for negative ions; a.m.u. range 50–3000; external or internal calibration using Electrospray Calibrant Solution (Fluka)). Samples were injected as solutions in acetonitrile, methanol, or water using a syringe; flow rate 3 μL/min; nebulizer gas nitrogen, flow rate 4 L/min; interface temperature 180 °C. The electronic absorption spectra were recorded on «Cary 100» (Varian) spectrophotometer. The fluorescence emission and fluorescence excitation spectra were recorded on «Cary Eclipse» (Varian) spectrofluorimiter. The fluorescence quantum yields were determined by the Parker-Rice method [1] with 3-methoxybenzanthrone in toluene (Φ = 0.1, λ_{irr} = 365 nm) as a standard luminophore [2]. Column chromatography was performed on Al_2O_3 (Brockmann activity grade II–III). Melting points were determined in glass capillaries on a PTP melting point apparatus and were not corrected.

X-ray crystal data: The elementary cell parameters of crystals and the three-dimensional intensity sets for compounds **5g**, **6e**, **11b**, and **13** were obtained using auto diffractometers indicated in Table S1, information is collected on the main experimental and crystallographic data. The structures were identified using the direct method and refined by the least-squares matrix method with respect to F^2 using the SHELXTL program [3] and anisotropic approximation for non-hydrogen atoms. The hydrogen atoms in the crystal structures were localized in the Fourier syntheses of the difference electron density. The coordinates and the isotropic thermal parameters were subsequently refined using the rider method (where it was possible) and with constraint imposition on the values of the isotropic thermal parameters [3].

Atomic coordinates, full tables of bond lengths, bond angles and thermal parameters have been deposited at the Cambridge Crystallographic Data Center (CCDC 1020684 (**5g**), CCDC 1020685 (**6e**), CCDC 1020686 (**11b**), CCDC 1020687 (**13**)).

Computational methods: The calculations were performed with the PBE0 hybrid functional [4] and 6-311+G** basis set using GAUSSIAN 09 set of programs [5]. Solvations effects were accounted for with the use of the PCM model [6] with the solvent parameters for dimethyl sulfoxide (ε = 46.7). All the structures were fully geometry optimized and the nature of the stationary points on the potential energy surfaces was characterized by calculations of the Hessian force constant matrices.

Synthesis: 2-Methylbenzoxazoles, 2-methylbenzothiazoles and 2,3,3-trimethylindolines were obtained from Fluka (Switzerland). 3,4,5,6-Tetrachloro-1,2-benzoquinone, 2-(benzoxazol-2-yl)-5,6,7-trichloro-1,3-tropolone (**5a**), 2-(benzoxazol-2-yl)-4,5,6,7-tetrachloro-1,3-tropolone (**6a**), 2-(2-ethoxycarbonyl-3,4-dichloro-6-hydroxyphenyl)benzoxazole (**11a**), 2-(2-ethoxycarbonyl-6-hydroxy-3,4,5-trichlorophenyl)benzoxazole (**11b**), 2-(5-chlorobenzothiazolyl)-5,6,7-trichloro-1,3-tropolone (**5e**) and 2-(5-chlorobenzothiazolyl)-4,5,6,7-tetrachloro-1,3-tropolone (**6e**) were prepared according to the known procedures [7,8].

Experimental section

General procedure for the synthesis of 2-(benzazolyl)-5,6,7-trichloro-1,3-tropolones (5b-d,5f) and 2-(3,3-dimethylindolyl)-5,6,7-thrichloro-1,3-tropolone (5g): A solution of 2-methylbenzazole (2,3,3-trimethylindoline) (10 mmol) and 2.46 g (10 mmol) of *o*-chloranil in 5 mL of dioxane was refluxed during 10–40 mins and then left for a day at room temperature. The precipitate was filtered off, washed with 10 mL of dioxane and subsequently with 10 mL of hexane, dried and recrystallized from benzene. For the compounds **5d,f,g** the deposited crystals were dissolved in chloroform and purified by column chromatography eluting with CH_2Cl_2. The yellow fraction was collected and recrystallized from benzene.

2-(5-Chlorobenzoxazol-2-yl)-5,6,7-trichloro-1,3-tropolone (5b): yellow crystals, yield 31%, mp 258-260 ºC. ^1H NMR ($CDCl_3$, 300 MHz): δ 7.33 (1H, s, CH_{trop}), 7.36-7.39 (1H, m, CH_{Ar}), 7.55-7.58 (1H, m, CH_{Ar}), 7.65 (1H, s, CH_{Ar}), 14.65 (1H, s, OH). ^{13}C NMR (DMSO-d^6, 151 MHz): δ 107.8, 111.9, 117.2, 120.3, 126.6, 133.1, 133.7, 134.5, 135.7, 140.6, 148.2, 162.3, 173.0, 176.7. IR: ν=3099, 3043, 2356, 1897, 1652, 1564, 1519, 1453, 1373, 1276, 1129, 1060, 856, 814, 788, 764, 712, 592 cm^{-1}. MS: m/z (%): 375 (8) [M^+], 349 (100), 321

(11), 284 (24), 147 (7), 137 (7), 119 (6), 99 (7), 84 (8), 63 (26), 51 (9), 38 (6). Anal. calc. for $C_{14}H_5Cl_4NO_3$: C 44.60; H 1.34; Cl 37.61; N 3.72. Found: C 44.46; H 1.22; Cl 37.52; N 3.58.

2-(5-Phenylbenzoxazol-2-yl)-5,6,7-trichloro-1,3-tropolone (5c): yellow crystals, yield 21%, mp 241-243 ºC. ^1H NMR ($CDCl_3$, 300 MHz): δ 7.33 (1H, s, CH_{trop}), 7.38 (1H, d, CH_{Ar}, J = 7.2 Hz), 7.43-7.50 (2H, m, CH_{Ar}), 7.56-7.74 (4H, m, CH_{Ar}), 7.83 (1H, s, CH_{Ar}), 15.02 (1H, s, OH). ^{13}C NMR (DMSO-d^6, 151 MHz): δ 106.9, 110.9, 114.1, 124.6, 126.6 (2C), 127.2, 128.6 (2C), 131.9, 132.3, 133.4, 133.7, 138.3, 139.2, 139.6, 146.8, 161.7, 171.7, 175.4. IR: ν=3093, 3068, 2356, 1652, 1519, 1470, 1374, 1270, 1131, 860, 822, 787, 754, 733, 694 cm^{-1}. MS: m/z (%): 417 (6) [M^+], 389 (100), 326 (29), 194 (25), 179 (17), 163 (19), 149 (29), 140 (99), 128 (31), 115 (77), 102 (33), 89 (43), 77 (44), 63 (86), 51 (42), 39 (30). Anal. Calc. for $C_{20}H_{10}CI_3NO_3$: C 57.38; H 2.41; Cl 25.40; N 3.35. Found: C 57.24; H 2.28; Cl 25.32; N 3.24.

2-(Benzothiazol-2-yl)-5,6,7-trichloro-1,3-tropolone (5d): yellow crystals, yield 35%, mp 237-239 ºC. ^1H NMR ($CDCl_3$, 300 MHz): *δ* 7.35 (1H, s, CH_{trop}), 7.47-7.59 (2H, m, CH_{Ar}), 7.62-7.80 (1H, m, CH_{Ar}), 7.90-7.95 (1H, m, CH_{Ar}), 17.01 (1H, s, OH). ^{13}C NMR (DMSO-d^6, 151 MHz): δ 112.1, 116.4, 122.1, 125.3, 127.5, 128.9, 133.0, 133.2, 134.5, 138.4, 140.2, 166.8, 174.0, 176.6. IR: *ν*=3095, 3022, 2366, 1602, 1559, 1519, 1485, 1446, 1392, 1353, 1129, 890, 862, 785, 766, 749, 587 cm^{-1}. Anal. Calc. for $C_{14}H_6CI_3NO_2S$: C 46.89; H 1.69; Cl 29.66; N 3.91. Found: C 46.74; H 1.56; Cl 29.52; N 3.84.

2-(5-Methylbenzothiazol-2-yl)-5,6,7-trichloro-1,3-tropolone (5f): yellow crystals, yield 32%, mp 230-232 ºC. ^1H NMR ($CDCl_3$, 300 MHz): *δ* 2.53 (3H, s, CH_3), 7.32 (1H, d, CH_{Ar}, J = 8.0 Hz), 7.34 (1H, s, CH_{trop}), 7.58 (1H, s, CH_{Ar}), 7.79 (1H, d, CH_{Ar}, J = 8.0 Hz), 16.82 (1H, s, OH). ^{13}C NMR (DMSO-d^6, 151 MHz): δ 20.9, 112.3, 116.2, 121.9, 126.2, 127.0, 133.0, 133.2, 134.8, 137.8, 138.7, 140.2, 166.8, 174.0, 176.9. IR: *ν*=3053, 2923, 1598, 1561, 1504, 1449, 1343, 1321, 1117, 1071, 902, 805, 787, 766, 598, 559 cm^{-1}. Anal. Calc. for $C_{15}H_8CI_3NO_2S$: C 48.35; H 2.16; Cl 28.54; N 3.76. Found: C 48.22; H 2.04; Cl 28.42; N 3.62.

2-(3,3-Dimethylindol-2-yl)-5,6,7-trichloro-1,3-tropolone (5g): yellow crystals, yield 60%, mp 167-168 ºC. ^1H NMR ($CDCl_3$, 300 MHz): *δ* 1.69 (6H, s, 3,3'-CH_3), 6.88 (1H, s, CH_{trop}), 7.20-7.36 (4H, m, CH_{Ar}), 14.79 (1H, br.s, OH). ^{13}C NMR (DMSO-d^6, 151 MHz): δ 24.1, 51.8, 111.9, 114.3, 121.9, 125.7, 128.0, 128.1, 130.8, 134.3, 134.7, 139.1, 141.5, 176.7, 182.4, 182.8. IR: *ν*=3046, 2979, 2929, 2868, 2359,1718, 1655, 1542, 1498, 1474, 1458, 1398, 1375, 1360, 1315, 1292, 1213, 1086, 1049, 1015, 942, 913, 858, 795, 770, 751, 734, 702, 668 cm^{-1}. MS: m/z (%): 367 (10) [M^+], 339 (40), 324 (40), 304 (3), 289 (31), 254 (15), 225 (13),

191 (23), 170 (5), 144 (100), 128 (15), 115 (48), 103 (22), 91 (19), 77 (43), 63 (26), 51 (327), 39 (32). Anal. Calc. for $C_{17}H_{12}CI_3NO_2$: C 55.39; H 3.28; Cl 28.85; N 3.80. Found: C 55.24; H 3.16; Cl 28.66; N 3.68.

2.4.2 General procedure for the synthesis of 2-(benzazol-2-yl)-4,5,6,7-tetrachloro-1,3-tropolones (6b–d) and 2-(3,3-dimethylindolyl)-4,5,6,7-tetrachloro-1,3-tropolone (6g).

A solution of 2-methylbenzazole (2,3,3-trimethylindoline) (10 mmol) and 4.92 g (20 mmol) of *o*-chloranil in 10 ml AcOH was allowed to stand at 50 ^{o}C during 72–96 hours. The precipitate was filtered, recrystallized with hot filtering from the mixture of solvents (chloroform/benzene), dissolved in chloroform and passed through the chromatographic column with silica gel (eluent/hexane - CH_2Cl_2 1:2). The yellow fraction was collected, the mother liquor diluted with water and extracted with chloroform (2 × 30 mL). The organic phase was washed with water (3 × 50 mL), dried during 3–4 h over Na_2SO_4, filtered and concentrated in vacuum. The crude product was purified by column chromatography with silica gel (eluent/hexane - CH_2Cl_2, 1:2) and the yellow fraction was collected.

2-(5-Chlorobenzoxazol-2-yl)-4,5,6,7-tetrachloro-1,3-tropolone (6b): yellow crystals, yield 33%, mp 264-266 ºC. ^{1}H NMR ($CDCl_3$, 300 MHz): δ 7.17-7.42 (2H, m, CH_{Ar}), 7.55-7.66 (1H, m, CH_{Ar}), 14.60 (1H, br.s, OH). ^{13}C NMR (DMSO-d^6, 151 MHz): δ (major isomer) 104.0, 112.4, 114.6, 125.2, 125.6, 127.9, 129.8, 136.9, 145.5, 162.4, 175.8. IR: ν=3101, 2384, 1896, 1656, 1520, 1454, 1371, 1297,1259, 1161, 1060, 923, 812, 762, 713, 592 cm^{-1}. MS: m/z (%): 383 (8) [M$^+$ –CO], 349 (3), 248 (2), 181 (8), 153 (12), 137 (22), 118 (22), 99 (23), 87 (17), 76 (23), 63 (100), 51 (35), 38 (11). Anal. Calc. for $C_{14}H_4CI_5NO_3$: C 40.87; H 0.98; Cl 43.08; N 3.40. Found: C 40.74; H 0.82; Cl 42.96; N 3.28.

2-(5-Phenylbenzoxazol-2-yl)-4,5,6,7-tetrachloro-1,3-tropolone (6c): yellow crystals, yield 15%, mp 252-254 ºC. ^{1}H NMR ($CDCl_3$, 300 MHz): 7.17-7.81 (8H, m, CH_{Ar}), 14.27 (1H, br.s, OH). ^{13}C NMR (DMSO-d^6, 151 MHz): δ 107.3, 111.5, 111.9, 116.9, 117.3, 124.0, 124.8, 126.6, 128.3, 128.8, 138.4, 139.6, 147.2, 164.2, 174.4. IR: ν=3092, 3030, 1655, 1519, 1470, 1373, 1271, 1131, 1119, 862, 822, 764, 755, 733, 694 cm^{-1}. MS: m/z (%): 425 (6) [M$^+$ - CO], 417 (67), 389 (100), 380 (28), 324 (38), 261 (25), 225 (13), 162 (19), 152 (13), 139 (100), 130 (12), 121 (12), 113 (18), 102 (25), 87 (14), 77 (7), 63 (20), 44 (22), 36 (53). Anal. Calc. for $C_{20}H_9CI_4NO_3$: C 53.02; H 2.00; Cl 31.30; N 3.09. Found: C 52.88; H 1.90; Cl 31.26; N 2.84.

2-(Benzothiazol-2-yl)-4,5,6,7-tetrachloro-1,3-tropolone (6d): yellow crystals, yield 10%, mp 244-246 ºC. ^{1}H NMR ($CDCl_3$, 300 MHz): δ 7.49-7.55 (1H, m, CH_{Ar}), 7.60-7.65 (1H, m,

CH_{Ar}), 7.73 (1H, d, CH_{Ar}, J = 8.1 Hz), 7.92 (1H, d, CH_{Ar}, J = 8.1 Hz), 14.99 (1H, br.s, OH). ^{13}C NMR (DMSO-d^6, 151 MHz): δ 111.4, 116.5, 122.4, 125.5, 127.8, 128.2, 129.7, 137.3, 138.5, 165.8, 175.3. IR: ν=3617, 3018, 2359, 1593, 1534, 1496, 1446, 1354, 1304, 1173, 1091, 946, 802, 774, 750, 718, 616 cm^{-1}. Anal. Calc. for $C_{14}H_5CI_4NO_2S$: C 42.78; H 1.28; Cl 36.08; N 3.56. Found: C 42.64; H 1.16; Cl 35.96; N 3.44.

2-(3,3-Dimethylindol-2-yl)-4,5,6,7-tetrachloro-1,3-tropolone (6g): yellow crystals, yield 29%, mp 169-172 ºC. ^{1}H NMR ($CDCl_3$, 300 MHz): δ 1.68 (6H, s, 3,3'-CH_3), 7.16-7.23 (1H, m, CH_{Ar}), 7.25-7.34 (3H, m, CH_{Ar}), 13.97 (1H, s, OH). ^{13}C NMR (DMSO-d^6, 151 MHz): δ 23.7 (2C), 51.9, 111.0, 114.6, 121.9, 125.9, 126.7, 128.0, 134.7, 139.0, 141.3, 175.6, 181.2. IR: ν= 3033, 2975, 2939, 1665, 1558, 1501, 1475, 1456, 1396, 1370, 1357, 1287, 1216, 1066, 931, 822, 782, 756, 686, 673 cm^{-1}. MS: m/z (%): 403 (64) [M$^+$], 374 (40), 360 (46), 352 (92), 338 (30), 330 (24), 323 (17), 302 (18), 288 (19), 259 (15), 225 (23), 190 (23), 181 (16), 170 (15), 155 (29), 144 (100), 130 (28), 115 (44), 103 (18), 89 (27), 77 (39), 63 (31), 51 (30), 39 (34). Anal. Calc. for $C_{17}H_{11}CI_4NO_2$: C 50.66; H 2.75; Cl 35.18; N 3.47. Found: C 50.42; H 2.56; Cl 35.02; N 3.34.

2.4.3 General procedure for the synthesis of 2-(2-alkoxycabonyl-6-hydroxyphenyl)benzoxa(thia)zoles (11c–g):

A solution of 0.82 mmol of 2-(2-benzoxa(thia)zol-2-yl)-1,3-tropolone **5a** (**6a** or **5e**) and 50–60 mL of an alcohol (MeOH, EtOH, iPrOH) was refluxed during 6–8 hours until the initial product was completely dissolved. The obtained solution was concentrated, cooled down, and the precipitate filtered off. The precipitate was dissolved in chloroform, passed through the chromatographic column with silica gel (eluent – ethyl acetate/CH_2Cl_2, 1:5), and the first colorless fraction was collected. The product was recrystallized from the respective alcohol (MeOH, EtOH, iPrOH) to give crystals of compounds **11c–g**.

2-(3,4-Dichloro-6-hydroxy-2-methoxycarbonylphenyl)benzoxazole (11c): colorless crystals, yield 72%, mp 161-163 ºC (methanol). ^{1}H NMR ($CDCl_3$, 250 MHz): δ 4.08 (3H, s, CH_3), 7.32 (1H, s, CH_{Ar}), 7.40-7.45 (2H, m, CH_{Ar}), 7.52-7.56 (1H, m, CH_{Ar}), 7.72-7.76 (1H, m, CH_{Ar}), 12.37 (1H, s, OH). ^{13}C NMR ($CDCl_3$, 63 MHz): δ 53.7, 107.6, 111.2, 120.0, 120.7, 126.2, 126.8, 130.2, 133.9, 138.2, 139.0, 149.3, 158.3, 160.3, 166.5. IR: ν= 2948, 2362, 2341, 1744, 1619, 1577, 1530, 1434, 1328, 1300, 1243, 1219, 1178, 1155, 1111, 1074, 1018, 945, 858, 829, 770, 744, 678, 576 cm^{-1}. MS: m/z (%): 317 (100) [M$^+$], 305 (76), 277 (54), 250 (7), 214 (24), 180 (18), 159 (11), 151 (6), 107 (7), 93 (8), 67 (60), 55 (11), 41 (49), 29 (12). Anal. Calc. for $C_{15}H_9C_{12}NO_4$: C 53.28; H 2.68; Cl 20.97; N 4.14. Found: C 53.12; H 2.54; Cl 20.76; N 4.02.

2-(6-Hydroxy-2-methoxycarbonyl-3,4,5-trichlorophenyl)benzoxazole (11d): colorless crystals, yield 67%, mp 182-184 ºC (methanol). ^{1}H NMR ($CDCl_3$, 250 MHz): δ 4.08 (3H, s, CH_3), 7.43-7.46 (2H, m, CH_{Ar}), 7.54-7.58 (1H, m, CH_{Ar}), 7.75-7.78 (1H, m, CH_{Ar}), 13.18 (1H, s, OH). ^{13}C NMR ($CDCl_3$, 63 MHz): δ 53.8, 107.4, 111.3, 120.1, 121.6, 126.4, 127.2, 131.5, 133.8, 137.2, 138.6, 149.5, 155.1, 160.0, 166.1. IR: ν= 3456, 2815, 2362, 2338, 1737, 1615, 1567, 1452, 1409, 1364, 1315, 1226, 1181, 1101, 1026, 947, 909, 884, 831, 809, 771, 740, 745, 730, 704, 605 cm^{-1}. MS: m/z (%): 371 (91) [M^+], 339 (100), 311 (50), 304 (9), 284 (11), 248 (45), 222 (9), 214 (25), 194 (10), 185 (17), 170 (11), 158 (10), 151 (16), 131 (9), 124 (11), 111 (7), 95 (9), 87 (9), 78 (16), 63 (75), 51 (24), 39 (37). Anal. Calc. for $C_{15}H_8Cl_3NO_4$: C 48.35; H 2.16; Cl 28.55; N 3.76. Found: C 48.18; H 2.04; Cl 28.42; N 3.62.

2-(3,4-Dichloro-6-hydroxy-2-isopropoxycarbonylphenyl)benzoxazole (11e): colorless crystals, yield 82%, mp 157-159 ºC (propan-2-ol). ^{1}H NMR ($CDCl_3$, 250 MHz): δ 1.46 (6H, d, $CH(CH_3)_2$, J = 6.3 Hz), 5.49 (1H, septet, CH, J = 6.3 Hz), 7.30 (1H, s, CH_{Ar}), 7.39-7.45 (2H, m, CH_{Ar}), 7.48-7.53 (1H, m, CH_{Ar}), 7.72-7.76 (1H, m, CH_{Ar}), 12.41 (1H, s, OH). ^{13}C NMR ($CDCl_3$, 63 MHz): δ 22.1 (2C), 71.2, 107.4, 111.2, 120.0, 120.5, 126.1, 126.8, 130.2, 134.6, 138.2, 139.1, 149.3, 158.3, 160.6, 165.5. IR: ν= 3454, 3074, 2979, 2931, 2362, 1732, 1620, 1576, 1529, 1435, 1375, 1348, 1321, 1301, 1222, 1182, 1159, 1103, 1073, 1001, 920, 904, 828, 782, 760, 735, 677, 575 cm^{-1}. MS: m/z (%): 365 (55) [M^+], 323 (8), 306 (31), 279 (100), 250 (11), 214 (24), 188 (9), 180 (21), 160 (10), 151 (11), 125 (10), 107 (14), 97 (8), 77 (7), 63 (29), 51 (8), 43 (58). Anal. Calc. for $C_{17}H_{13}Cl_2NO_4$: C 55.76; H, 3.58; Cl 19.36; N 3.82. Found: C 55.60; H 3.44; Cl 19.22; N 3.68.

2-(6-Hydroxy-2-isopropoxycarbonyl-3,4,5-trichlorophenyl)benzoxazole (11f): colorless crystals, yield, 94%, mp 173-174 ºC (propan-2-ol). ^{1}H NMR ($CDCl_3$, 250 MHz): δ 1.46 (6H, d, $CH(CH_3)_2$, J = 6.3 Hz), 5.49 (1H, septet, CH, J = 6.3 Hz), 7.42-7.47 (2H, m, CH_{Ar}), 7.50-7.55 (1H, m, CH_{Ar}), 7.75-7.79 (1H, m, CH_{Ar}), 13.23 (1H, s, OH). ^{13}C NMR ($CDCl_3$, 63 MHz): δ 22.0 (2C), 71.4, 107.2, 111.2, 120.1, 121.8, 126.3, 127.1, 130.1, 132.1, 137.2, 138.7, 149.5, 155.1, 160.3, 165.0. IR: ν= 3448, 2988, 2358, 2341, 1732, 1618, 1568, 1450, 1409, 1374, 1341, 1299, 1222, 1180, 1096, 1013, 903, 802, 780, 767, 733, 603 cm^{-1}. MS: m/z (%): 399 (50) [M^+], 365 (7), 357 (6), 340 (31), 313 (100), 306 (6), 287 (6), 279 (19), 250 (31), 222 (6), 214 (20), 194 (6), 185 (7), 171 (5), 151 (9), 124 (13), 108 (5), 92 (5), 63 (29), 51 (7), 43 (62), 29 (12). Anal. Calc. for $C_{17}H_{12}Cl_3NO_4$: C 50.96; H 3.02; Cl 26.55; N 3.50. Found: C 50.84; H 2.90; Cl 26.44; N 3.38.

5-Chloro-2-(3,4-dichloro-2-ethoxycarbonyl-6-hydroxyphenyl)benzothiazole (11g): yellow crystals, yield 86%, mp 202-204 ºC (ethanol). ^{1}H NMR (DMSO-d^6, 300 MHz): δ 1.26

(3H, t, CH_3, *J* = 7.2 Hz), 4.42 (2H, qu, CH_2, *J* = 7.2 Hz), 7.37 (1H, s, CH_{Ar}), 7.53 (1H, dd, CH_{Ar}, J_1 = 8.4 Hz, J_2 = 1.5 Hz), 8.03 (1H, d, CH_{Ar}, *J* = 1.5 Hz), 8.20 (1H, d, CH_{Ar}, *J* = 8.4 Hz), 12.43 (1H, s, OH). ^{13}C NMR ($CDCl_3$, 151 MHz): δ 13.8, 63.3, 113.5, 120.5, 120.7, 122.1, 122.2, 127.0, 131.3, 133.5, 134.5, 137.1, 150.6, 157.7, 166.2, 166.3. IR: *ν*=1735, 1560, 1548, 1474, 1424, 1349, 1315, 1269, 1248, 1196, 1150, 1110, 1067, 1024, 1007, 964, 863, 812, 750, 708, 674 cm^{-1}. Anal. Calc. for $C_{16}H_{10}Cl_3NO_3S$: C 47.72; H 2.50; Cl 26.41; N 3.48. Found: C 47.64; H 2.40; Cl 26.34; N 3.28.

2.4.4 Synthesis of 5,7-dichloro-2-(3,3-dimethylindol-2-yl)-6-ethoxy-1,3-tropolone (13). A solution of 0.3 mmol of 2-(3,3-dimethylindolyl)-5,6,7-thrichloro-1,3-tropolone (**5g**) in 40 mL of ethanol was alkalified with 40% NaOH solution (0.1 mL) and refluxed during 15 min. Formation of the product was controlled using TLC. The solution was cooled down, diluted with water, extracted with chloroform (2 × 30 mL), and the chloroform solution was washed with water (3 × 50 mL) and then dried during 3–4 hours over Na_2SO_4. The solvent was evaporated and the crude product purified by column chromatography with silica gel (eluent – ethyl acetate/CH_2Cl_2, 1:1). The second yellow fraction was collected (R_f ~ 0,3). The recrystallization was performed from the mixture of chloroform/propan-2-ol (1:5). Yellow crystals, yield 72% mp: 156-158 ºC (propan-2-ol). ^{1}H NMR ($CDCl_3$, 300 MHz): *δ* 1.41 (3H, t, CH_3, *J* = 6.9 Hz), 1.70 (6H, s, CH_3(3',3')), 4.03 (2H, qu, CH_2, *J* = 6.9 Hz), 6.91 (1H, s, CH_{trop}), 7.18-7.33 (4H, m, CH_{Ar}), 15.17 (1H, s, OH). ^{13}C NMR (DMSO-d^6, 151 MHz): δ 14.7, 24.3, 57.3, 68.5, 113.0, 113.7, 121.6, 125.3, 127.6, 127.7, 131.6, 134.1, 138.9, 141.5, 147.4, 177.4, 182.4, 183.0. IR: *ν* = 3051, 2979, 2936, 2889, 1951, 1911, 1797, 1719, 1642, 1617, 1574, 1528, 1476, 1454, 1396, 1374, 1327, 1221, 1108, 1028, 1013, 926, 879, 863, 753, 681 cm^{-1}. MS: m/z (%): 377 (16) [M^+], 349 (64), 342 (7), 334 (37), 320 (34), 306 (24), 299 (13), 270 (25), 254 (6), 191 (12), 178 (22), 154 (12), 144 (78), 130 (18), 115 (41), 103 (18), 91 (14), 77 (28), 63 (12), 51 (13), 40 (23), 29 (100). Anal. Calc. for $C_{19}H_{17}Cl_2NO_3$: C 60.33; H 4.53; Cl 18.75; N 3.70. Found: C 60.22; H 4.40; Cl 18.62; N 3.58.

Principal experimental data of the X-ray diffraction analyses

Table S1: Principal experimental data of the X-ray diffraction analyses

Compound	**5g**	**6e**	**11b**	**13**
Formula	$C_{17}H_{12}Cl_3NO_2$	$C_{31}H_{11}Cl_{10}N_4O_4S_2$	$C_{16}H_{10}Cl_3NO_4$	$C_{19} H_{17} Cl_2 N O_3$
Crystal size	0.45x0.40x0.40	0.40x0.30x0.25	0.35x0.30x0.30	0.50x030x020
Color	Yellow	Yellow	Colorless	Colorless
M	368.63	894.04	386.60	378.24
Diffractometer	KUMA KM-4	Bruker P-4	Xcalibur, Eos	Xcalibur, Eos
Temperature	RT	RT	-123°C	-123°C
Spatial group.	P 2(1)/c	P-1	P-1	P-1
a(Å)	10.968(2)	9.7780(10)	7.3811(2)	5.8538(5)
b(Å)	14.884(3)	13.7780(10)	10.0145(4)	11.4536(7)
c(Å)	20.665(4)	14.2690(10)	12.2021(4)	13.8762(9)
α^o	90.00	63.080(10)	66.803(3)	95.898(5)
β^o	104.31(3)	87.310(10)	82.220(2)	93.599(6)
γ^o	90.00	77.610(10)	74.139(3)	104.120(6)
V(Å^3)	3268.8(11)	1671.3(2)	797.08(4)	893.75(11)
Z	8	2	2	2
D g/cm^3	1.498	1.777	1.611	1.405
μ, cm^{-1}	0.568	1.002	0.596	0.381
θ range	1.70–26.02	2.42-25.05	2.86-33.06	2.96-26.32

Total number of reflections	6401	5645	5591	6315
Reflections with I> 2σ(I)	3628	4330	4886	3375
Refined parameters	415	448	247	231
R	0.0463	0.0430	0.0301	0.0404
wR_2	0.1169	0.0608	0.0363	0.0894
Irradiation	Mo(Kα)	Mo(Kα)	Mo(Kα)	Mo(Kα)
GOF	1.007	1.012	1.032	1.028

Crystallographic data for 5g, 6e, 11b and 13

Table S2: The main bond lengths (d) and valence angles (ω) of (**5g**).

Bond	d/Å	Bond	d/Å
Cl(1)-C(7)	1.723(3)	Cl(2)-C(6)	1.737(3)
Cl(3)-C(5)	1.745(3)	N(1)-C(8)	1.333(3)
N(1)-C(10)	1.406(3)	O(1)-C(1)	1.210(3)
O(2)-C(3)	1.248(3)	C(1)-C(2)	1.468(4)
C(1)-C(7)	1.513(4)	C(2)-C(8)	1.415(4)
C(2)-C(3)	1.447(4)	C(3)-C(4)	1.472(4)
C(4)-C(5)	1.326(4)	C(5)-C(6)	1.440(5)
C(6)-C(7)	1.328(4)	C(8)-C(9)	1.536(4)
C(9)-C(11)	1.510(4)	C(9)-C(17)	1.541(4)
C(9)-C(16)	1.550(4)	C(10)-C(11)	1.374(4)
C(10)-C(15)	1.375(4)	C(11)-C(12)	1.385(4)
C(12)-C(13)	1.392(5)	C(13)-C(14)	1.386(5)
C(14)-C(15)	1.386(4)	Cl(21)-C(25)	1.735(3)
Cl(22)-C(26)	1.725(3)	Cl(23)-C(27)	1.719(3)
N(21)-C(28)	1.344(3)	N(21)-C(30)	1.401(4)
O(21)-C(21)	1.210(3)	O(22)-C(23)	1.248(3)
C(21)-C(22)	1.466(4)	C(21)-C(27)	1.504(4)
C(22)-C(28)	1.407(4)	C(22)-C(23)	1.445(4)
C(23)-C(24)	1.470(4)	C(24)-C(25)	1.326(5)
C(25)-C(26)	1.453(5)	C(26)-C(27)	1.334(4)
C(28)-C(29)	1.546(4)	C(29)-C(31)	1.512(4)
C(29)-C(37)	1.530(4)	C(29)-C(36)	1.538(4)
C(30)-C(35)	1.383(4)	C(30)-C(31)	1.385(4)
C(31)-C(32)	1.371(4)	C(32)-C(33)	1.398(5)
C(33)-C(34)	1.382(5)	C(34)-C(35)	1.376(5)

Angle	ω/degrees	Angle	ω/degrees
C(8)-N(1)-C(10)	112.9(2)	O(1)-C(1)-C(2)	125.7(3)
O(1)-C(1)-C(7)	117.1(2)	C(2)-C(1)-C(7)	116.8(2)
C(8)-C(2)-C(3)	118.4(2)	C(8)-C(2)-C(1)	121.8(2)
C(3)-C(2)-C(1)	119.7(2)	O(2)-C(3)-C(2)	123.5(2)
O(2)-C(3)-C(4)	114.9(2)	C(2)-C(3)-C(4)	121.1(2)
C(5)-C(4)-C(3)	128.3(3)	C(4)-C(5)-C(6)	125.7(3)
C(4)-C(5)-Cl(3)	117.8(3)	C(6)-C(5)-Cl(3)	116.5(2)
C(7)-C(6)-C(5)	122.5(3)	C(7)-C(6)-Cl(2)	120.5(3)
C(5)-C(6)-Cl(2)	117.0(2)	C(6)-C(7)-C(1)	126.5(3)
C(6)-C(7)-Cl(1)	121.4(2)	C(1)-C(7)-Cl(1)	112.0(2)
N(1)-C(8)-C(2)	120.2(2)	N(1)-C(8)-C(9)	108.2(2)
C(2)-C(8)-C(9)	131.5(2)	C(11)-C(9)-C(8)	100.9(2)
C(11)-C(9)-C(17)	111.2(2)	C(8)-C(9)-C(17)	111.6(2)
C(11)-C(9)-C(16)	109.0(2)	C(8)-C(9)-C(16)	111.8(2)
C(17)-C(9)-C(16)	111.9(2)	C(11)-C(10)-C(15)	124.0(3)
C(11)-C(10)-N(1)	107.9(2)	C(15)-C(10)-N(1)	128.1(3)
C(10)-C(11)-C(12)	119.2(3)	C(10)-C(11)-C(9)	110.1(2)
C(12)-C(11)-C(9)	130.7(3)	C(11)-C(12)-C(13)	118.1(3)

C(14)-C(13)-C(12)	121.4(3)	C(15)-C(14)-C(13)	120.7(3)
C(10)-C(15)-C(14)	116.7(3)	C(28)-N(21)-C(30)	113.3(2)
O(21)-C(21)-C(22)	124.3(3)	O(21)-C(21)-C(27)	117.7(2)
C(22)-C(21)-C(27)	117.7(2)	C(28)-C(22)-C(23)	119.3(2)
C(28)-C(22)-C(21)	119.8(2)	C(23)-C(22)-C(21)	120.9(3)
O(22)-C(23)-C(22)	122.8(3)	O(22)-C(23)-C(24)	114.9(3)
C(22)-C(23)-C(24)	122.1(3)	C(25)-C(24)-C(23)	130.2(3)
C(24)-C(25)-C(26)	126.0(3)	C(24)-C(25)-Cl(21)	118.3(3)
C(26)-C(25)-Cl(21)	115.7(2)	C(27)-C(26)-C(25)	122.4(3)
C(27)-C(26)-Cl(22)	120.5(3)	C(25)-C(26)-Cl(22)	117.1(2)
C(26)-C(27)-C(21)	127.3(3)	C(26)-C(27)-Cl(23)	121.0(2)
C(21)-C(27)-Cl(23)	111.6(2)	N(21)-C(28)-C(22)	121.4(2)
N(21)-C(28)-C(29)	107.4(2)	C(22)-C(28)-C(29)	130.9(2)
C(31)-C(29)-C(37)	111.6(3)	C(31)-C(29)-C(36)	107.4(2)
C(37)-C(29)-C(36)	112.0(3)	C(31)-C(29)-C(28)	101.4(2)
C(37)-C(29)-C(28)	110.3(2)	C(36)-C(29)-C(28)	113.6(3)
C(35)-C(30)-C(31)	122.9(3)	C(35)-C(30)-N(21)	129.0(3)
C(31)-C(30)-N(21)	108.2(2)	C(32)-C(31)-C(30)	119.7(3)
C(32)-C(31)-C(29)	130.7(3)	C(30)-C(31)-C(29)	109.6(3)
C(31)-C(32)-C(33)	118.4(3)	C(34)-C(33)-C(32)	120.8(3)
C(35)-C(34)-C(33)	121.4(3)	C(34)-C(35)-C(30)	116.9(3)

Table S3: The main bond lengths (d) and valence angles (ω) of (**7e**).

Bond	d/Å	Bond	d/Å
S(1)-C(8)	1.734(3)	S(1)-C(10)	1.749(3)
Cl(1)-C(4)	1.717(3)	Cl(2)-C(5)	1.729(3)
Cl(3)-C(6)	1.722(3)	Cl(4)-C(7)	1.721(3)
Cl(5)-C(13)	1.732(3)	O(1)-C(1)	1.229(3)
O(2)-C(3)	1.230(3)	N(1)-C(8)	1.346(3)
N(1)-C(9)	1.389(4)	C(1)-C(2)	1.444(4)
C(1)-C(7)	1.503(4)	C(2)-C(8)	1.419(4)
C(2)-C(3)	1.433(4)	C(3)-C(4)	1.499(4)
C(4)-C(5)	1.339(4)	C(5)-C(6)	1.455(4)
C(6)-C(7)	1.345(4)	C(9)-C(10)	1.379(4)
C(9)-C(14)	1.390(4)	C(10)-C(11)	1.395(4)
C(11)-C(12)	1.369(5)	C(12)-C(13)	1.388(5)
C(13)-C(14)	1.374(4)	S(21)-C(28)	1.734(3)
S(21)-C(30)	1.745(3)	Cl(21)-C(24)	1.717(3)
Cl(22)-C(25)	1.736(3)	Cl(23)-C(26)	1.732(3)
Cl(24)-C(27)	1.721(3)	Cl(25)-C(33)	1.737(3)
O(21)-C(21)	1.231(4)	O(22)-C(23)	1.240(3)
N(21)-C(28)	1.338(3)	N(21)-C(29)	1.387(4)
C(21)-C(22)	1.433(4)	C(21)-C(27)	1.501(4)
C(22)-C(28)	1.433(4)	C(22)-C(23)	1.439(4)
C(23)-C(24)	1.504(4)	C(24)-C(25)	1.337(4)
C(25)-C(26)	1.459(4)	C(26)-C(27)	1.334(4)
C(29)-C(34)	1.384(4)	C(29)-C(30)	1.393(4)
C(30)-C(31)	1.394(4)	C(31)-C(32)	1.371(4)

C(32)-C(33)	1.392(4)	C(33)-C(34)	1.375(4)
C(41)-C(42)	1.361(5)	C(41)-C(43)#1	1.366(5)
C(42)-C(43)#2	1.376(6)	C(43)-C(41)#1	1.366(5)
C(43)-C(42)#3	1.376(6)		

Angle	ω/degrees	Angle	ω/degrees
C(8)-S(1)-C(10)	90.78(13)	C(8)-N(1)-C(9)	115.4(2)
O(1)-C(1)-C(2)	122.5(3)	O(1)-C(1)-C(7)	118.1(2)
C(2)-C(1)-C(7)	118.7(3)	C(8)-C(2)-C(3)	116.8(2)
C(8)-C(2)-C(1)	116.7(2)	C(3)-C(2)-C(1)	126.2(2)
O(2)-C(3)-C(2)	123.4(3)	O(2)-C(3)-C(4)	117.1(3)
C(2)-C(3)-C(4)	118.5(2)	C(5)-C(4)-C(3)	127.8(3)
C(5)-C(4)-Cl(1)	120.9(2)	C(3)-C(4)-Cl(1)	111.2(2)
C(4)-C(5)-C(6)	124.6(3)	C(4)-C(5)-Cl(2)	119.1(2)
C(6)-C(5)-Cl(2)	116.2(2)	C(7)-C(6)-C(5)	124.3(3)
C(7)-C(6)-Cl(3)	120.2(2)	C(5)-C(6)-Cl(3)	115.4(2)
C(6)-C(7)-C(1)	127.5(3)	C(6)-C(7)-Cl(4)	120.2(2)
C(1)-C(7)-Cl(4)	112.3(2)	N(1)-C(8)-C(2)	124.1(3)
N(1)-C(8)-S(1)	110.9(2)	C(2)-C(8)-S(1)	124.9(2)
C(10)-C(9)-N(1)	111.7(2)	C(10)-C(9)-C(14)	121.8(3)
N(1)-C(9)-C(14)	126.5(3)	C(9)-C(10)-C(11)	120.7(3)
C(9)-C(10)-S(1)	111.1(2)	C(11)-C(10)-S(1)	128.2(2)
C(12)-C(11)-C(10)	117.8(3)	C(11)-C(12)-C(13)	120.8(3)
C(14)-C(13)-C(12)	122.3(3)	C(14)-C(13)-Cl(5)	118.4(3)
C(12)-C(13)-Cl(5)	119.3(2)	C(13)-C(14)-C(9)	116.6(3)
C(28)-S(21)-C(30)	90.50(13)	C(28)-N(21)-C(29)	115.8(2)
O(21)-C(21)-C(22)	121.6(3)	O(21)-C(21)-C(27)	117.4(3)
C(22)-C(21)-C(27)	120.4(3)	C(21)-C(22)-C(28)	115.6(2)
C(21)-C(22)-C(23)	127.4(3)	C(28)-C(22)-C(23)	117.0(2)
O(22)-C(23)-C(22)	122.3(3)	O(22)-C(23)-C(24)	16.7(3)
C(22)-C(23)-C(24)	120.2(2)	C(25)-C(24)-C(23)	128.6(3)
C(25)-C(24)-Cl(21)	120.2(2)	C(23)-C(24)-Cl(21)	111.2(2)
C(24)-C(25)-C(26)	125.5(3)	C(24)-C(25)-Cl(22)	118.9(2)
C(26)-C(25)-Cl(22)	115.4(2)	C(27)-C(26)-C(25)	125.6(3)
C(27)-C(26)-Cl(23)	119.2(2)	C(25)-C(26)-Cl(23)	115.2(2)
C(26)-C(27)-C(21)	128.3(3)	C(26)-C(27)-Cl(24)	119.9(2)
C(21)-C(27)-Cl(24)	111.7(2)	N(21)-C(28)-C(22)	123.8(2)
N(21)-C(28)-S(21)	111.3(2)	C(22)-C(28)-S(21)	124.9(2)
N(21)-C(29)-C(34)	127.0(3)	N(21)-C(29)-C(30)	111.0(2)
C(34)-C(29)-C(30)	122.0(3)	C(29)-C(30)-C(31)	120.1(3)
C(29)-C(30)-S(21)	111.3(2)	C(31)-C(30)-S(21)	128.5(2)
C(32)-C(31)-C(30)	118.1(3)	C(31)-C(32)-C(33)	120.9(3)
C(34)-C(33)-C(32)	122.1(3)	C(34)-C(33)-Cl(25)	119.1(2)
C(32)-C(33)-Cl(25)	118.9(2)	C(33)-C(34)-C(29)	116.8(3)

Table S4: The main bond lengths (d) and valence angles (ω) in compound (**11b**).

Bond	d/Å	Bond	d/Å
Cl(1)-C(5)	1.7371(17)	Cl(2)-C(7)	1.7331(17)
O(1)-C(1)	1.217(2)	O(2)-C(3)	1.2563(19)

O(3)-C(6)	1.368(2)	O(3)-C(18)	1.453(2)
N(1)-C(8)	1.334(2)	N(1)-C(15)	1.403(2)
N(1)-H(1)	0.869(19)	C(1)-C(2)	1.471(2)
C(1)-C(7)	1.508(2)	C(2)-C(8)	1.422(2)
C(2)-C(3)	1.444(2)	C(3)-C(4)	1.475(2)
C(4)-C(5)	1.335(2)	C(5)-C(6)	1.461(2)
C(6)-C(7)	1.343(2)	C(8)-C(9)	1.546(2)
C(9)-C(10)	1.518(2)	C(9)-C(17)	1.543(2)
C(9)-C(16)	1.543(2)	C(10)-C(11)	1.383(2)
C(10)-C(15)	1.386(2)	C(11)-C(12)	1.393(3)
C(12)-C(13)	1.388(3)	C(13)-C(14)	1.389(2)
C(14)-C(15)	1.384(2)	C(18)-C(19)	1.491(3)

Angle	ω/degree	Angle	ω/degree
C(6)-O(3)-C(18)	112.93(14)	C(8)-N(1)-C(15)	113.09(13)
C(8)-N(1)-H(1)	118.5(13)	C(15)-N(1)-H(1)	128.0(12)
O(1)-C(1)-C(2)	123.71(16)	O(1)-C(1)-C(7)	117.04(15)
C(2)-C(1)-C(7)	118.90(14)	C(8)-C(2)-C(3)	118.71(14)
C(8)-C(2)-C(1)	120.31(14)	C(3)-C(2)-C(1)	120.98(15)
O(2)-C(3)-C(2)	123.16(15)	O(2)-C(3)-C(4)	113.74(14)
C(2)-C(3)-C(4)	122.74(14)	C(5)-C(4)-C(3)	129.44(16)
C(4)-C(5)-C(6)	127.41(16)	C(4)-C(5)-Cl(1)	117.41(13)
C(6)-C(5)-Cl(1)	115.18(13)	C(7)-C(6)-O(3)	120.51(16)
C(7)-C(6)-C(5)	121.74(16)	O(3)-C(6)-C(5)	117.65(15)
C(6)-C(7)-C(1)	128.90(16)	C(6)-C(7)-Cl(2)	118.04(14)
C(1)-C(7)-Cl(2)	112.96(12)	N(1)-C(8)-C(2)	120.27(14)
N(1)-C(8)-C(9)	108.12(14)	C(2)-C(8)-C(9)	131.38(14)
C(10)-C(9)-C(17)	110.62(14)	C(10)-C(9)-C(16)	108.30(13)
C(17)-C(9)-C(16)	112.11(13)	C(10)-C(9)-C(8)	100.96(12)
C(17)-C(9)-C(8)	110.40(13)	C(16)-C(9)-C(8)	113.88(13)
C(11)-C(10)-C(15)	119.20(15)	C(11)-C(10)-C(9)	131.34(15)
C(15)-C(10)-C(9)	109.45(14)	C(10)-C(11)-C(12)	118.72(16)
C(13)-C(12)-C(11)	121.04(17)	C(12)-C(13)-C(14)	120.91(17)
C(15)-C(14)-C(13)	116.86(16)	C(14)-C(15)-C(10)	123.26(15)
C(14)-C(15)-N(1)	128.40(15)	C(10)-C(15)-N(1)	108.33(14)
O(3)-C(18)-C(19)	108.02(18)		

Table S5: The main bond lengths (d) and valence angles (ω) in compound (**13**).

Bond	d/Å	Bond	d/Å
Cl(1)-C(4)	1.7122(10)	Cl(3)-C(6)	1.7261(10)
Cl(2)-C(5)	1.7132(11)	O(1)-C(8)	1.3621(12)
O(1)-C(10)	1.3839(12)	O(2)-C(3)	1.3397(12)

O(2)-H(2)	0.845(17)	O(3)-C(7)	1.2015(12)
O(4)-C(7)	1.3295(12)	O(4)-C(15)	1.4642(13)
N(1)-C(8)	1.3084(12)	N(1)-C(9)	1.3995(14)
C(1)-C(6)	1.3862(14)	C(1)-C(2)	1.4071(13)
C(1)-C(7)	1.5092(13)	C(2)-C(3)	1.4130(13)
C(2)-C(8)	1.4511(14)	C(3)-C(4)	1.4016(15)
C(4)-C(5)	1.3912(14)	C(5)-C(6)	1.3992(14)
C(9)-C(10)	1.3907(13)	C(9)-C(14)	1.3973(14)
C(10)-C(11)	1.3814(14)	C(11)-C(12)	1.3896(15)
C(12)-C(13)	1.4052(16)	C(13)-C(14)	1.3851(16)
C(15)-C(16)	1.4970(17)		
Angle	ω/degree	Angle	ω/degree
C(8)-O(1)-C(10)	104.25(7)	C(7)-O(4)-C(15)	115.16(8)
C(8)-N(1)-C(9)	104.70(8)	C(6)-C(1)-C(2)	120.05(9)
C(6)-C(1)-C(7)	117.08(8)	C(2)-C(1)-C(7)	122.87(9)
C(1)-C(2)-C(3)	119.69(9)	C(1)-C(2)-C(8)	122.45(9)
C(3)-C(2)-C(8)	117.85(8)	O(2)-C(3)-C(4)	117.60(9)
O(2)-C(3)-C(2)	123.18(9)	C(4)-C(3)-C(2)	119.21(9)
C(5)-C(4)-C(3)	120.75(9)	C(5)-C(4)-Cl(1)	120.48(8)
C(3)-C(4)-Cl(1)	118.74(8)	C(4)-C(5)-C(6)	119.71(9)
C(4)-C(5)-Cl(2)	120.08(8)	C(6)-C(5)-Cl(2)	120.19(8)
C(1)-C(6)-C(5)	120.59(9)	C(1)-C(6)-Cl(3)	119.36(8)
C(5)-C(6)-Cl(3)	120.01(8)	O(3)-C(7)-O(4)	125.99(9)
O(3)-C(7)-C(1)	123.40(9)	O(4)-C(7)-C(1)	110.54(8)
N(1)-C(8)-O(1)	114.99(9)	N(1)-C(8)-C(2)	125.24(9)
O(1)-C(8)-C(2)	119.76(8)	C(10)-C(9)-C(14)	120.18(10)
C(10)-C(9)-N(1)	108.24(8)	C(14)-C(9)-N(1)	131.58(9)
C(11)-C(10)-O(1)	127.72(9)	C(11)-C(10)-C(9)	124.46(9)
O(1)-C(10)-C(9)	107.82(9)	C(10)-C(11)-C(12)	114.90(9)
C(11)-C(12)-C(13)	121.87(10)	C(14)-C(13)-C(12)	122.12(10)
C(13)-C(14)-C(9)	116.46(10)	O(4)-C(15)-C(16)	107.25(10)

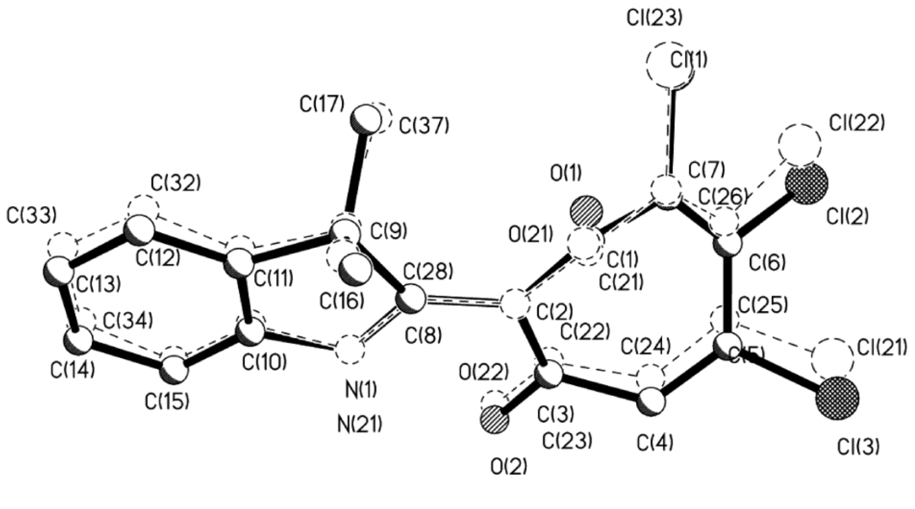

Figure S1: Superimposition of two independent molecules **5g** with respect to positions of atoms N(1), C(2), and C(9). The atomic numbers of the second molecule are increased by a factor of 20 relative to the first one. Hydrogen atoms are not shown.

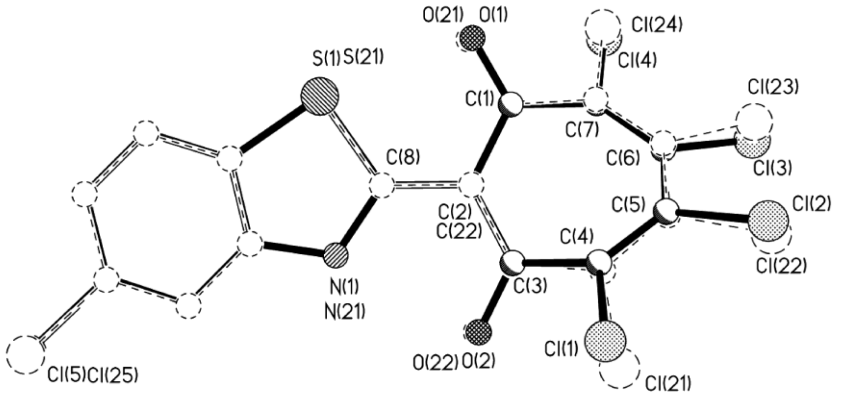

Figure S2: Superimposition of two independent molecules **6e** with respect to positions of atoms S(1), C(2), and C(9). The atomic numbers of the second molecule are increased by 20 relative to the first one. Hydrogen atoms are not shown.

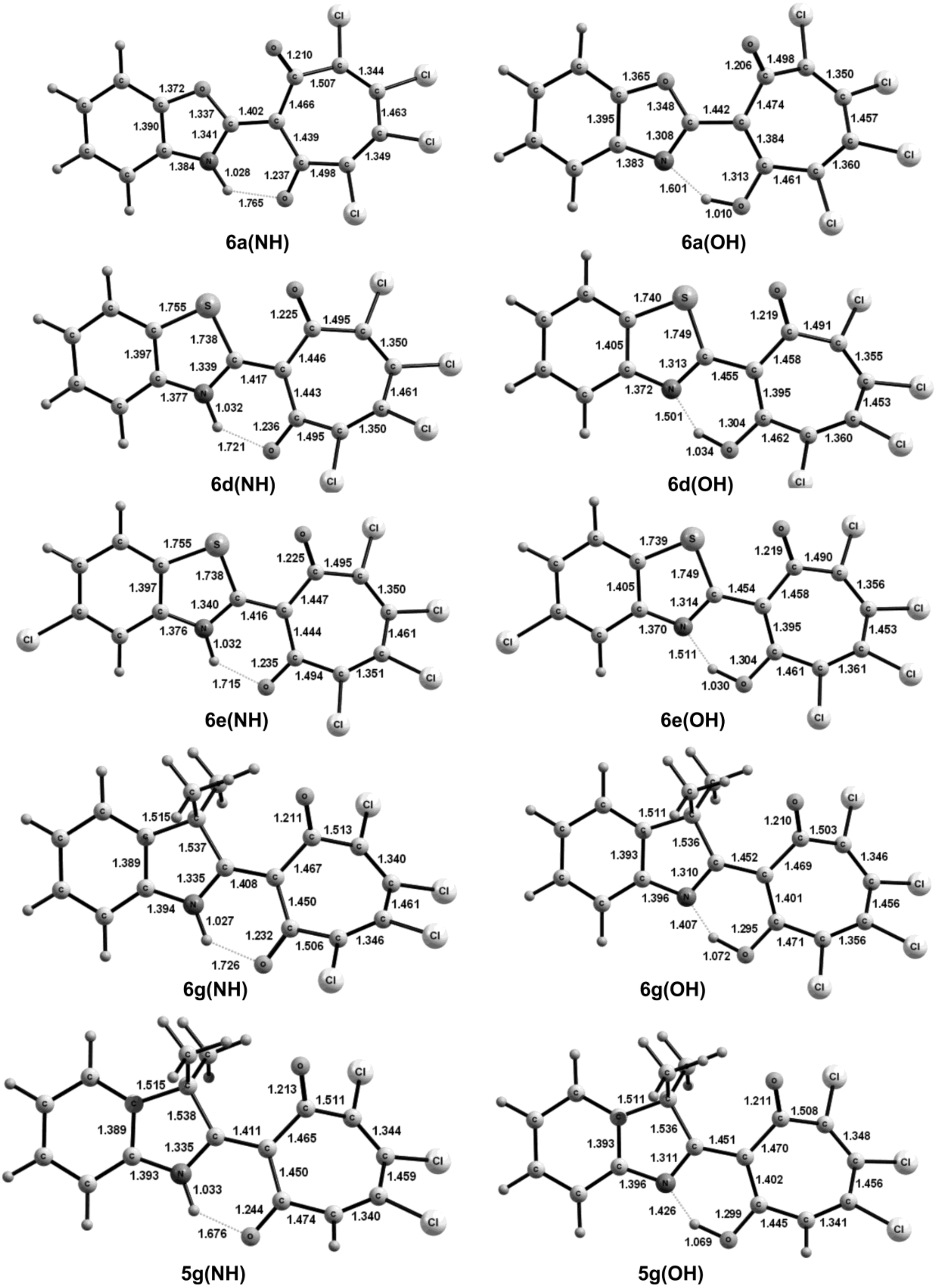

Figure S3: Optimized geometries of the (NH) and (OH) tautomeric forms of the compounds **6a**, **6d**, **6e**, **6g** and **5g** in the gas phase according to the PBE0/6-311+G** calculations. The bond lengths are given in angstroms.

Table S6: Optimized structure (Cartesian coordinates, Å) of **6a(NH)**.

	X	Y	Z		X	Y	Z
1 C	-6.137042	0.651874	0.153323	20 Cl	3.937195	1.524191	-1.112679
2 C	-6.159329	-0.570787	-0.522717	21 Cl	4.363522	-1.081516	0.559046
3 C	-4.992331	-1.273232	-0.808835	22 H	-1.979665	-1.858369	-0.895058
4 C	-3.805892	-0.694997	-0.387386	23 H	-4.906082	2.176476	1.102239
5 C	-3.798751	0.522327	0.282925	24 H	-7.071076	1.163555	0.355494
6 C	-4.942091	1.230871	0.575820	25 H	-5.012007	-2.221443	-1.332481
7 C	-1.720205	-0.118974	0.109629	26 Cl	2.093288	-3.168782	0.427786
8 N	-2.474401	-1.060883	-0.475654	27 O	-2.501869	0.858364	0.580894
9 C	-0.321151	-0.116969	0.192958				
10 H	-7.111918	-0.985246	-0.833084				
11 C	2.770437	-0.605294	0.089022				
12 C	2.622604	0.811530	-0.246002				
13 C	1.556675	1.552603	0.102116				
14 C	0.325221	1.046036	0.808212				
15 O	-0.162675	1.693339	1.705966				
16 C	0.346133	-1.286206	-0.315841				
17 C	1.779395	-1.520238	0.049525				
18 O	-0.247100	-2.195808	-0.907743				
19 Cl	1.461701	3.227994	-0.261768				

Table S7: Optimized structure (Cartesian coordinates, Å) of **6a(OH)**.

	X	Y	Z		X	Y	Z
1 C	-6.099251	0.693450	-0.063977	20 Cl	3.808860	1.666635	-1.127425
2 C	-6.120956	-0.618006	-0.558384	21 Cl	4.405856	-1.001774	0.302200
3 C	-4.960658	-1.367204	-0.695145	22 H	-1.240505	-2.196402	-0.610083
4 C	-3.768360	-0.756225	-0.319434	23 H	-4.881989	2.324726	0.699643
5 C	-3.772231	0.550183	0.168375	24 H	-7.031552	1.239829	0.026277
6 C	-4.913033	1.312960	0.314493	25 H	-4.976461	-2.381840	-1.075379
7 C	-1.755881	-0.195304	0.144110	26 Cl	2.242895	-3.152074	0.370927
8 N	-2.456939	-1.196931	-0.320421	27 O	-2.483518	0.895592	0.456472
9 C	-0.320149	-0.187957	0.279659				
10 H	-7.071860	-1.057037	-0.839681				
11 C	2.764442	-0.551885	0.022763				
12 C	2.551474	0.862021	-0.258944				
13 C	1.474457	1.563036	0.155943				
14 C	0.305762	0.993516	0.899518				
15 O	-0.176733	1.576589	1.838608				
16 C	0.366985	-1.321777	-0.116012				
17 C	1.803010	-1.512041	0.075867				
18 O	-0.247880	-2.379786	-0.590773				
19 Cl	1.326644	3.247300	-0.127189				

Table S8: Optimized structure (Cartesian coordinates, Å) of **6d(NH)**.

	X	Y	Z		X	Y	Z
1 C	-6.343962	-0.188493	-0.061173	20 Cl	4.389108	-1.184417	0.899571
2 C	-6.073077	1.001829	0.622274	21 Cl	4.336416	1.411997	-0.772623
3 C	-4.771481	1.417611	0.849791	22 H	-1.896552	1.614300	0.945910
4 C	-3.741216	0.611690	0.372898	23 H	-5.525861	-1.912936	-1.061853
5 C	-4.005462	-0.578543	-0.309135	24 H	-7.372717	-0.489116	-0.225801
6 C	-5.315253	-0.990390	-0.532291	25 H	-4.556761	2.338911	1.379944
7 C	-1.591420	-0.101503	-0.052623	26 Cl	1.894283	3.247129	-0.441270
8 N	-2.386106	0.828067	0.491433	27 S	-2.517380	-1.370144	-0.797499
9 C	-0.176478	-0.027012	-0.021806				
10 H	-6.895445	1.610739	0.981263				
11 C	2.856157	0.772331	-0.151274				
12 C	2.896724	-0.639953	0.219256				
13 C	1.883004	-1.514491	0.048425				
14 C	0.510728	-1.219334	-0.467314				
15 O	-0.085066	-2.094806	-1.083599				
16 C	0.408609	1.210682	0.433452				
17 C	1.780847	1.580329	-0.031192				
18 O	-0.241262	2.076510	1.029120				
19 Cl	2.082103	-3.174605	0.444945				

Table S9: Optimized structure (Cartesian coordinates, Å) of **6d(OH)**.

	X	Y	Z		X	Y	Z
1 C	-6.341343	-0.164060	0.128871	20 Cl	4.301240	-1.290982	0.979412
2 C	-6.024286	1.061157	0.733138	21 Cl	4.422668	1.375758	-0.503019
3 C	-4.711903	1.475000	0.859250	22 H	-1.283405	1.914759	0.676044
4 C	-3.701583	0.643120	0.367825	23 H	-5.598901	-1.944309	-0.827120
5 C	-4.025074	-0.583896	-0.235893	24 H	-7.380042	-0.464323	0.042921
6 C	-5.349983	-0.997745	-0.360419	25 H	-4.456158	2.419034	1.327337
7 C	-1.638323	-0.067175	-0.119977	26 Cl	2.026073	3.238870	-0.454276
8 N	-2.354050	0.895017	0.414924	27 S	-2.578616	-1.392685	-0.767006
9 C	-0.186269	0.008010	-0.159708				
10 H	-6.822949	1.692221	1.107563				
11 C	2.875920	0.733286	-0.091436				
12 C	2.859471	-0.678554	0.251961				
13 C	1.832160	-1.529255	0.011673				
14 C	0.504589	-1.204278	-0.583177				
15 O	-0.076081	-2.055557	-1.233776				
16 C	0.407174	1.225027	0.174976				
17 C	1.801366	1.567495	-0.099055				
18 O	-0.299566	2.227498	0.616275				
19 Cl	1.983627	-3.200342	0.372748				

Table S10: Optimized structure (Cartesian coordinates, Å) of **6e(NH)**.

	X	Y	Z		X	Y	Z
1 C	-5.716678	-0.895505	-0.376121	20 Cl	4.789453	1.683109	-0.712847
2 C	-5.575673	0.343186	0.256433	21 H	-1.504158	1.370047	0.722709
3 C	-4.335619	0.899518	0.519443	22 H	-4.700783	-2.571121	-1.252546
4 C	-3.219066	0.169685	0.123088	23 H	-6.708650	-1.288854	-0.561430
5 C	-3.338211	-1.070276	-0.508711	24 H	-4.243541	1.860153	1.011432
6 C	-4.594238	-1.610232	-0.761799	25 Cl	2.178182	3.293208	-0.564621
7 C	-0.996168	-0.354015	-0.175732	26 S	-1.762502	-1.738593	-0.894757
8 N	-1.899137	0.521221	0.287415	27 Cl	-7.001704	1.210953	0.727258
9 C	0.400932	-0.144297	-0.087343				
10 C	3.348027	0.932014	-0.127682				
11 C	3.502564	-0.453947	0.308077				
12 C	2.584236	-1.428233	0.135478				
13 C	1.214296	-1.286266	-0.445113				
14 O	0.726185	-2.240446	-1.039044				
15 C	0.846672	1.163356	0.331884				
16 C	2.198744	1.640567	-0.088920				
17 O	0.089961	1.988011	0.854946				
18 Cl	2.921250	-3.044423	0.610829				
19 Cl	5.007116	-0.826090	1.071084				

Table S11: Optimized structure (Cartesian coordinates, Å) of **6e(OH)**.

	X	Y	Z		X	Y	Z
1 C	-5.725638	-0.901145	-0.226112	20 Cl	4.856174	1.679280	-0.436372
2 C	-5.547621	0.373935	0.327076	21 H	-0.923113	1.710422	0.452792
3 C	-4.297320	0.932830	0.497341	22 H	-4.766698	-2.632575	-1.051354
4 C	-3.190985	0.179743	0.094621	23 H	-6.728002	-1.296024	-0.339528
5 C	-3.358736	-1.099756	-0.459823	24 H	-4.171847	1.918266	0.928719
6 C	-4.629820	-1.646052	-0.623089	25 Cl	2.292501	3.295590	-0.591090
7 C	-1.046776	-0.338813	-0.256304	26 S	-1.814446	-1.781807	-0.878822
8 N	-1.881222	0.570749	0.193981	27 Cl	-6.952156	1.274109	0.812763
9 C	0.390599	-0.121401	-0.231510				
10 C	3.360995	0.905523	-0.064529				
11 C	3.464934	-0.483886	0.349201				
12 C	2.538155	-1.443182	0.107458				
13 C	1.214553	-1.279539	-0.557303				
14 O	0.747638	-2.215620	-1.183068				
15 C	0.846499	1.163199	0.064641				
16 C	2.212706	1.628555	-0.162960				
17 O	0.025526	2.111539	0.419879				
18 Cl	2.835146	-3.072544	0.556285				
19 Cl	4.922144	-0.916072	1.168173				

Table S12: Optimized structure (Cartesian coordinates, Å) of **6g(NH)**.

	X	Y	Z		X	Y	Z
1 C	6.149686	0.033541	-0.344141	20 Cl	-4.166726	-1.365024	-1.330600
2 C	5.847151	1.182878	-1.070071	21 Cl	-4.611735	1.128013	0.590279
3 C	4.531927	1.620608	-1.206383	22 H	1.625738	1.800507	-1.032190
4 C	3.552238	0.861303	-0.589314	23 H	5.388425	-1.603000	0.836626
5 C	3.831481	-0.286171	0.142023	24 H	7.181975	-0.286884	-0.255276
6 C	5.140825	-0.710165	0.270758	25 H	4.288940	2.513829	-1.771226
7 C	1.512642	0.124972	0.111374	26 Cl	-2.246944	3.161168	0.638594
8 N	2.172168	1.059393	-0.577782	27 C	2.542280	-0.849473	0.704444
9 C	0.106297	0.143494	0.183074	28 C	2.333444	-2.298801	0.245092
10 H	6.646247	1.747029	-1.538687	29 H	2.278380	-2.363057	-0.844633
11 C	-3.030799	0.666900	0.073167	30 H	3.187349	-2.897558	0.573465
12 C	-2.886183	-0.717682	-0.370827	31 H	1.433233	-2.733870	0.677295
13 C	-1.813694	-1.454115	-0.050086	32 C	2.582355	-0.740603	2.240777
14 C	-0.639520	-0.973007	0.773463	33 H	3.421040	-1.335881	2.612507
15 O	-0.307995	-1.616002	1.744523	34 H	2.740644	0.294920	2.554159
16 C	-0.596011	1.294019	-0.350954	35 H	1.655566	-1.110865	2.675803
17 C	-2.011906	1.545908	0.096100				
18 O	-0.056889	2.184938	-1.009701				
19 Cl	-1.624415	-3.080278	-0.573150				

Table S13: Optimized structure (Cartesian coordinates, Å) of **6g(OH)**.

	X	Y	Z		X	Y	Z
1 C	6.120175	0.072631	-0.503609	20 Cl	-4.111897	-1.417506	-1.320567
2 C	5.787321	1.308352	-1.056651	21 Cl	-4.625841	1.150456	0.370528
3 C	4.469920	1.756705	-1.065107	22 H	1.089358	2.059802	-0.658446
4 C	3.510665	0.925964	-0.504491	23 H	5.414785	-1.705670	0.492976
5 C	3.830456	-0.309577	0.054158	24 H	7.154224	-0.255380	-0.512478
6 C	5.141841	-0.747278	0.061232	25 H	4.198843	2.715057	-1.494097
7 C	1.552043	0.136739	0.171410	26 Cl	-2.338235	3.187293	0.590647
8 N	2.135777	1.151383	-0.416471	27 C	2.567934	-0.928947	0.607866
9 C	0.104448	0.156256	0.282712	28 C	2.304169	-2.311048	-0.001486
10 H	6.566090	1.928178	-1.488133	29 H	2.188611	-2.249648	-1.086912
11 C	-3.008183	0.661052	0.028855	30 H	3.158383	-2.960973	0.207721
12 C	-2.844581	-0.729068	-0.371559	31 H	1.417870	-2.778860	0.427302
13 C	-1.784290	-1.475536	-0.009471	32 C	2.691204	-1.000592	2.142856
14 C	-0.624798	-1.000147	0.820915	33 H	3.534123	-1.649210	2.397767
15 O	-0.260210	-1.664385	1.764133	34 H	2.890975	-0.010864	2.562554
16 C	-0.569958	1.317564	-0.114707	35 H	1.781931	-1.401212	2.587377
17 C	-1.998612	1.558616	0.140891				
18 O	0.056444	2.341107	-0.601571				
19 Cl	-1.642499	-3.126996	-0.459648				

Table S14: Optimized structure (Cartesian coordinates, Å) of **5g(NH)**.

	X	Y	Z		X	Y	Z
1 C	-5.954602	0.119584	-0.491275	20 Cl	4.506193	0.705044	-1.179710
2 C	-5.748554	-1.196519	-0.897400	21 Cl	4.674298	-1.825418	0.674580
3 C	-4.482055	-1.773785	-0.845348	22 H	-1.599433	-2.168349	-0.465606
4 C	-3.449635	-0.979241	-0.375415	23 H	-5.068893	1.919201	0.301790
5 C	-3.633439	0.334705	0.037255	24 H	-6.950285	0.546303	-0.544549
6 C	-4.895663	0.895617	-0.016184	25 H	-4.313559	-2.797446	-1.161006
7 C	-1.368392	-0.279989	0.229365	26 C	-2.314813	0.893135	0.533283
8 N	-2.099925	-1.294736	-0.235594	27 C	-1.942114	2.181781	-0.209225
9 C	0.034563	-0.400647	0.320267	28 H	-1.832230	2.001402	-1.281625
10 H	-6.585405	-1.782564	-1.261737	29 H	-2.744141	2.912281	-0.072418
11 C	3.128288	-1.167966	0.232848	30 H	-1.022150	2.618767	0.177556
12 C	3.126253	0.199515	-0.275102	31 C	-2.435097	1.127163	2.052782
13 C	2.093309	1.034232	-0.066942	32 H	-3.227058	1.859839	2.231807
14 C	0.850432	0.740798	0.741480	33 H	-2.710930	0.202729	2.567697
15 O	0.527407	1.546641	1.588870	34 H	-1.496337	1.498813	2.458758
16 C	0.640376	-1.688493	0.040302	35 H	2.209319	-2.975267	0.703981
17 C	2.052285	-1.955029	0.370514				
18 O	-0.008902	-2.680678	-0.335425				
19 Cl	2.095138	2.637467	-0.690181				

Table S15: Optimized structure (Cartesian coordinates, Å) of **5g(OH)**.

	X	Y	Z		X	Y	Z
1 C	-5.944219	0.088686	-0.593712	20 Cl	4.542155	0.739705	-1.065935
2 C	-5.734928	-1.269622	-0.828729	21 Cl	4.634521	-1.966875	0.436338
3 C	-4.472994	-1.835542	-0.675520	22 H	-1.099738	-2.390300	-0.157871
4 C	-3.440084	-0.995793	-0.283058	23 H	-5.072740	1.972327	-0.007067
5 C	-3.637666	0.362067	-0.041138	24 H	-6.936672	0.506744	-0.725149
6 C	-4.895034	0.917014	-0.191761	25 H	-4.297327	-2.889914	-0.858534
7 C	-1.419974	-0.269639	0.261649	26 C	-2.328592	0.961509	0.417534
8 N	-2.099320	-1.331258	-0.093972	27 C	-1.902851	2.145777	-0.456825
9 C	0.026727	-0.388939	0.362078	28 H	-1.773073	1.842258	-1.499070
10 H	-6.567190	-1.892950	-1.138165	29 H	-2.682279	2.912251	-0.425874
11 C	3.102630	-1.201097	0.165369	30 H	-0.978232	2.597667	-0.096683
12 C	3.126300	0.188323	-0.247670	31 C	-2.493944	1.378794	1.893253
13 C	2.101718	1.041029	-0.016443	32 H	-3.284479	2.132017	1.956452
14 C	0.843265	0.761774	0.757654	33 H	-2.795519	0.524740	2.505951
15 O	0.486434	1.582211	1.576633	34 H	-1.567718	1.791604	2.287066
16 C	0.611648	-1.647027	0.160318	35 H	2.167664	-3.018007	0.558975
17 C	2.007269	-1.970310	0.328985				
18 O	-0.106035	-2.712796	-0.083758				
19 Cl	2.190602	2.678525	-0.532588				

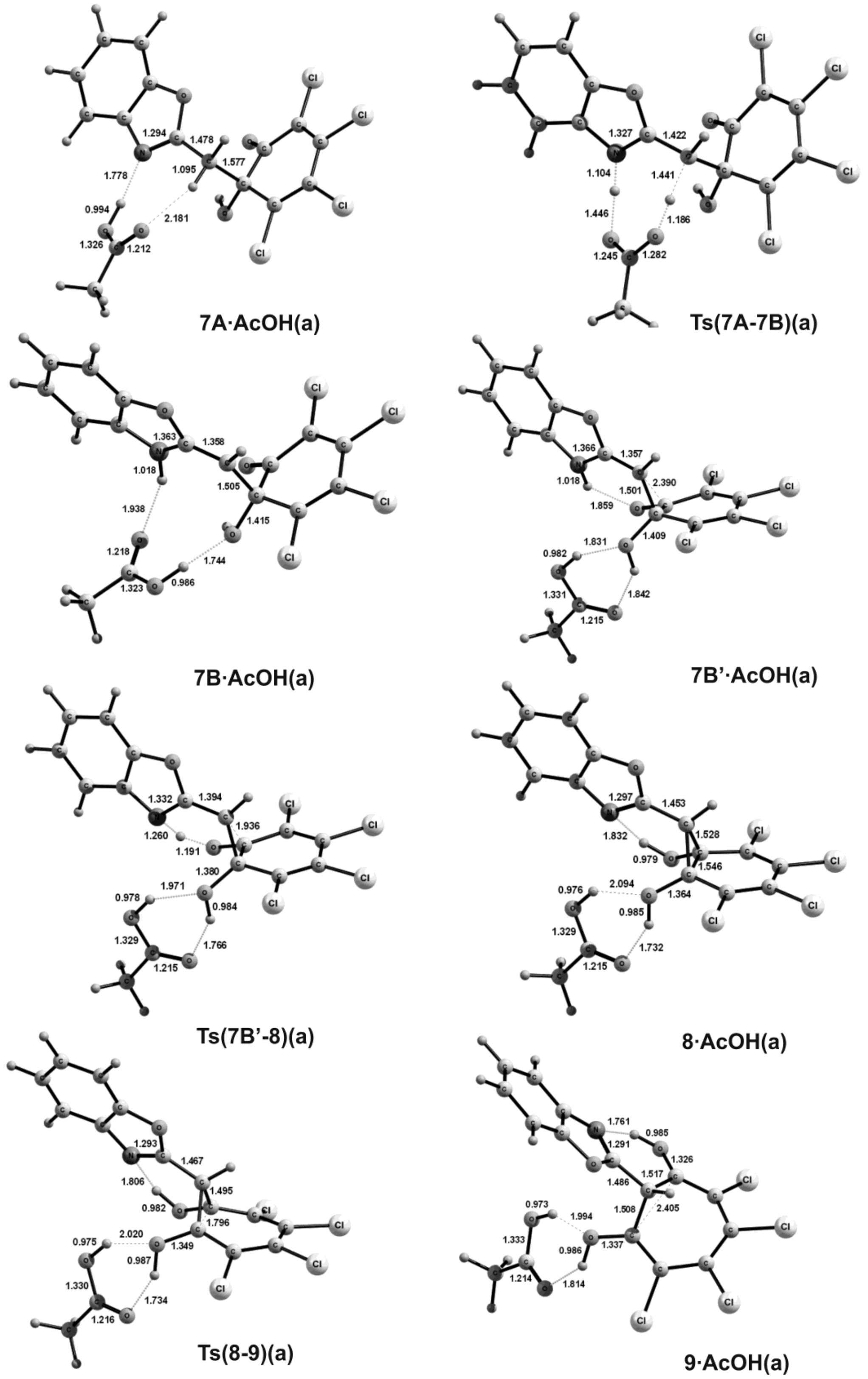

Figure S4: Optimized geometries of the intermediates **7A·AcOH(a)**, **7B·AcOH(a)**, **7B’·AcOH(a)**, **8·AcOH(a)**, **9·AcOH(a)** and transition states **Ts(7A-7B)(a)**, **Ts(7B’-8)(a)**,

Ts(8-9)(a) calculated using the PBE0/6-311+G** method in the gas phase. The bond lengths are given in angstroms.

Table S16: Optimized structure (Cartesian coordinates, Å) of **7A·AcOH(a)**.

	X	Y	Z		X	Y	Z
1 C	1.941381	1.226833	0.340265	20 C	-1.446318	-0.275737	-0.505969
2 C	3.034396	0.565639	-0.089576	21 C	-0.177339	0.468034	-0.651283
3 C	3.054021	-0.900511	-0.114897	22 H	0.408371	0.034942	-1.465769
4 C	0.925222	-0.970267	1.061109	23 H	-0.420597	1.511688	-0.878493
5 C	2.047600	-1.644378	0.416764	24 Cl	1.856763	2.925830	0.402208
6 C	0.674794	0.488200	0.675861	25 Cl	4.441705	1.414058	-0.596622
7 O	-0.018871	1.121557	1.695420	26 Cl	4.412650	-1.674229	-0.805646
8 O	0.152801	-1.503175	1.826012	27 Cl	2.088963	-3.351604	0.512792
9 C	-4.775380	-1.064138	0.241165	28 H	-0.476428	0.420701	2.183731
10 C	-5.342865	-2.317940	0.063624	29 O	-1.640047	3.308817	-1.075627
11 C	-3.435384	-0.924507	-0.105734	30 O	-3.271157	2.811816	0.375545
12 H	-6.384585	-2.471353	0.323483	31 C	-2.542389	3.654603	-0.343604
13 C	-4.604208	-3.396531	-0.443108	32 C	-2.960754	5.081300	-0.142792
14 C	-2.722488	-2.010781	-0.607652	33 H	-2.383451	5.732528	-0.796013
15 H	-5.089446	-4.359080	-0.562676	34 H	-4.028577	5.189969	-0.346227
16 C	-3.265549	-3.266676	-0.794700	35 H	-2.798741	5.362994	0.901016
17 H	-2.684993	-4.093958	-1.184204	36 H	-2.961790	1.879368	0.222839
18 H	-5.343653	-0.228234	0.632568	37 O	-1.451663	-1.584549	-0.863346
19 N	-2.576347	0.166703	-0.058220				

Table S17: Optimized structure (Cartesian coordinates, Å) of **Ts(7A-7B)(a)**.

	X	Y	Z		X	Y	Z
1 C	2.077638	1.067767	0.195838	20 C	-1.437770	-0.157102	-0.468272
2 C	3.034025	0.194742	-0.177475	21 C	-0.169296	0.463415	-0.635381
3 C	2.813438	-1.249758	-0.055908	22 H	0.373606	0.027003	-1.472823
4 C	0.773537	-0.832227	1.202055	23 H	-0.583824	1.823413	-0.870248
5 C	1.724220	-1.752814	0.578747	24 Cl	2.291360	2.754469	0.098626
6 C	0.720760	0.588913	0.641614	25 Cl	4.550400	0.742013	-0.780881
7 O	0.169982	1.430499	1.600460	26 Cl	3.987386	-2.306090	-0.714834
8 O	-0.011735	-1.149458	2.070978	27 Cl	1.473938	-3.426397	0.838823
9 C	-4.822665	-0.546315	0.581312	28 H	-0.258061	0.847390	2.246444
10 C	-5.666050	-1.549940	0.117030	29 O	-0.908155	2.954426	-1.017010
11 C	-3.530977	-0.539772	0.077216	30 O	-2.457139	2.799554	0.602722
12 H	-6.685340	-1.594221	0.484332	31 C	-1.747675	3.451479	-0.185170
13 C	-5.235467	-2.505192	-0.809053	32 C	-1.838375	4.950391	-0.187647
14 C	-3.120529	-1.493547	-0.846194	33 H	-2.763682	5.279441	0.283035
15 H	-5.925953	-3.273591	-1.138159	34 H	-0.991522	5.340936	0.385136
16 C	-3.939842	-2.495076	-1.319626	35 H	-1.756228	5.336712	-1.204244
17 H	-3.593228	-3.227216	-2.038459	36 H	-2.441862	1.354165	0.563207
18 H	-5.155366	0.195304	1.297578	37 O	-1.810262	-1.242856	-1.171187
19 N	-2.434621	0.285091	0.287505				

Table S18: Optimized structure (Cartesian coordinates, Å) of **7B·AcOH(a)**.

	X	Y	Z		X	Y	Z
1 C	2.089388	1.184381	-0.540334	20 C	-1.202700	-0.591484	-0.803669
2 C	3.202109	0.431487	-0.459559	21 C	0.035011	-0.061406	-0.980453
3 C	3.196137	-0.833060	0.284094	22 H	0.537701	-0.307283	-1.905931
4 C	0.993223	-0.298187	1.170873	23 H	-1.230474	2.853780	0.107342
5 C	2.125175	-1.213061	1.025091	24 Cl	2.017963	2.639888	-1.431824
6 C	0.786280	0.729058	0.057358	25 Cl	4.659970	0.922495	-1.230638
7 O	0.076267	1.816897	0.617485	26 Cl	4.586936	-1.827432	0.202981
8 O	0.194319	-0.335504	2.086153	27 Cl	2.098523	-2.633494	1.978313
9 C	-4.478977	-1.317783	0.506536	28 H	-0.216215	1.514811	1.496457
10 C	-5.417223	-2.122257	-0.142215	29 O	-2.021603	3.442789	0.108076
11 C	-3.260689	-1.147588	-0.126533	30 O	-2.728415	1.965022	1.630507
12 H	-6.384336	-2.281352	0.322031	31 C	-2.895752	2.987540	0.991063
13 C	-5.141080	-2.727627	-1.366139	32 C	-4.102423	3.863057	1.114221
14 C	-2.998984	-1.752908	-1.349882	33 H	-4.792962	3.444782	1.843277
15 H	-5.893815	-3.349841	-1.836907	34 H	-3.796862	4.867373	1.417525
16 C	-3.909657	-2.549258	-2.003009	35 H	-4.589744	3.953464	0.140275
17 H	-3.679895	-3.009653	-2.956208	36 H	-2.076889	0.291086	0.901852
18 H	-4.691341	-0.847639	1.459405	37 O	-1.733014	-1.415610	-1.753213
19 N	-2.111756	-0.446929	0.202000				

Table S19: Optimized structure (Cartesian coordinates, Å) of **7B'·AcOH(a)**.

	X	Y	Z		X	Y	Z
1 C	1.992241	0.001674	-1.286566	20 C	-1.573370	-0.975999	-0.743985
2 C	3.002023	-0.553206	-0.590042	21 C	-0.267516	-0.869523	-1.097382
3 C	2.831025	-0.893845	0.821829	22 H	0.120975	-1.632103	-1.757628
4 C	0.633401	0.192502	0.845191	23 H	-1.333830	2.495124	-0.587930
5 C	1.694113	-0.579173	1.493066	24 Cl	2.128828	0.369536	-2.950692
6 C	0.626953	0.264637	-0.689815	25 Cl	4.514766	-0.890752	-1.338102
7 O	0.110418	1.502813	-1.120474	26 Cl	4.107774	-1.707481	1.619266
8 O	-0.260809	0.724366	1.482131	27 Cl	1.483062	-0.909581	3.160777
9 C	-4.777580	-0.364330	0.791790	28 H	0.596593	2.250920	-0.713093
10 C	-5.900893	-1.169389	0.595845	29 O	-1.766326	3.271540	-0.171252
11 C	-3.623541	-0.728651	0.121781	30 O	0.368922	3.902878	0.069701
12 H	-6.824264	-0.916884	1.105327	31 C	-0.816273	4.106684	0.241377
13 C	-5.865051	-2.284117	-0.238327	32 C	-1.369119	5.308182	0.937689
14 C	-3.599495	-1.847378	-0.703933	33 H	-0.566166	6.007388	1.160681
15 H	-6.758682	-2.884152	-0.367460	34 H	-2.130741	5.783937	0.316303
16 C	-4.696732	-2.648462	-0.913437	35 H	-1.853443	4.992894	1.865828
17 H	-4.650363	-3.514603	-1.562231	36 H	-1.924588	0.481324	0.690103
18 H	-4.809428	0.505344	1.437526	37 O	-2.342441	-1.989489	-1.233057
19 N	-2.352944	-0.181854	0.047780				

Table S20: Optimized structure (Cartesian coordinates, Å) of **Ts(7B'-8)(a)**.

	X	Y	Z		X	Y	Z
1 C	2.097905	0.422721	-1.171012	20 C	-1.444246	-0.917434	-0.837963
2 C	3.082209	-0.261301	-0.550101	21 C	-0.062622	-0.805747	-0.988138
3 C	2.826938	-0.981383	0.692792	22 H	0.466801	-1.612211	-1.476780
4 C	0.531332	-0.123528	0.723672	23 H	-1.657977	2.349041	-0.151802
5 C	1.604633	-0.935646	1.280983	24 Cl	2.345088	1.251874	-2.647789
6 C	0.695199	0.454228	-0.650868	25 Cl	4.667384	-0.315529	-1.217473
7 O	0.007787	1.621609	-0.914062	26 Cl	4.095470	-1.905128	1.385617
8 O	-0.442410	0.250562	1.476491	27 Cl	1.267119	-1.725811	2.762832
9 C	-4.685743	-0.396615	0.494105	28 H	0.350085	2.366080	-0.369413
10 C	-5.782909	-1.204763	0.211593	29 O	-2.099619	3.072505	0.336045
11 C	-3.483250	-0.734278	-0.109656	30 O	0.003339	3.806949	0.591550
12 H	-6.740079	-0.975170	0.666914	31 C	-1.188772	3.923397	0.797261
13 C	-5.685151	-2.302284	-0.647782	32 C	-1.793707	5.029859	1.599005
14 C	-3.405625	-1.835546	-0.958081	33 H	-1.026004	5.751664	1.868856
15 H	-6.565504	-2.904145	-0.844009	34 H	-2.590693	5.512371	1.029284
16 C	-4.479886	-2.643472	-1.259371	35 H	-2.244212	4.612205	2.503386
17 H	-4.388670	-3.493112	-1.925046	36 H	-1.460459	0.220673	0.858955
18 H	-4.762179	0.459695	1.153841	37 O	-2.114897	-1.944779	-1.411153
19 N	-2.221226	-0.151858	-0.073994				

Table S21: Optimized structure (Cartesian coordinates, Å) of **8·AcOH(a)**.

	X	Y	Z		X	Y	Z
1 C	1.904800	0.155748	-1.360231	20 C	-1.489461	-1.107375	-0.156060
2 C	3.006607	-0.358264	-0.766713	21 C	-0.039742	-1.014453	-0.167876
3 C	3.007268	-0.718364	0.648332	22 H	0.474488	-1.935487	-0.417448
4 C	0.627222	-0.098102	0.857515	23 H	-1.883053	2.059336	-0.028659
5 C	1.909502	-0.548116	1.420674	24 Cl	1.908570	0.701392	-2.988996
6 C	0.627513	0.278786	-0.642311	25 Cl	4.457633	-0.576503	-1.667275
7 O	-0.235650	1.256504	-1.042118	26 Cl	4.458744	-1.344476	1.330166
8 O	-0.140075	0.641479	1.709146	27 Cl	1.919478	-0.854276	3.108646
9 C	-4.858762	-0.436471	0.519180	28 H	0.069173	2.150288	-0.760451
10 C	-5.913405	-1.243924	0.114139	29 O	-2.265797	2.884745	0.325862
11 C	-3.577876	-0.848266	0.168029	30 O	-0.252179	3.744743	-0.164761
12 H	-6.928380	-0.958608	0.368569	31 C	-1.371831	3.867621	0.290635
13 C	-5.703015	-2.419773	-0.617886	32 C	-1.897250	5.135842	0.880812
14 C	-3.394505	-2.021385	-0.561063	33 H	-1.178017	5.937837	0.730270
15 H	-6.557075	-3.018736	-0.914360	34 H	-2.857559	5.390654	0.427790
16 C	-4.425836	-2.839522	-0.975394	35 H	-2.068298	4.987902	1.950514
17 H	-4.250886	-3.746177	-1.541702	36 H	-1.084540	0.470314	1.516685
18 H	-5.018315	0.475440	1.082724	37 O	-2.050390	-2.179487	-0.762885
19 N	-2.326770	-0.292123	0.407172				

Table S22: Optimized structure (Cartesian coordinates, Å) of **Ts(8-9)(a)**.

	X	Y	Z		X	Y	Z
1 C	1.771871	0.094273	-1.360971	20 C	-1.556674	-1.103568	-0.027055
2 C	2.813659	-0.617903	-0.811265	21 C	-0.093743	-0.993850	-0.049455
3 C	2.861543	-0.945791	0.578867	22 H	0.405777	-1.927758	-0.296466
4 C	0.563310	-0.200474	1.034243	23 H	-1.672043	2.247353	0.102703
5 C	1.873661	-0.571354	1.461963	24 Cl	1.954917	0.958267	-2.848418
6 C	0.509355	0.206573	-0.714658	25 Cl	4.175611	-1.002129	-1.802641
7 O	-0.363945	1.190285	-1.015618	26 Cl	4.284094	-1.709018	1.195274
8 O	-0.168483	0.600065	1.829289	27 Cl	2.176433	-0.474402	3.158276
9 C	-4.912650	-0.569802	0.803274	28 H	0.055227	2.084140	-0.996709
10 C	-5.975798	-1.259935	0.237625	29 O	-1.935286	3.119924	0.448724
11 C	-3.637589	-0.904298	0.360175	30 O	-0.018909	3.751878	-0.526427
12 H	-6.986977	-1.029925	0.555062	31 C	-1.002126	4.015212	0.138036
13 C	-5.779380	-2.248690	-0.736145	32 C	-1.293869	5.366691	0.701516
14 C	-3.468236	-1.890295	-0.609772	33 H	-0.582826	6.090662	0.309851
15 H	-6.640690	-2.760860	-1.150667	34 H	-2.317320	5.661921	0.461894
16 C	-4.508892	-2.590817	-1.185896	35 H	-1.212183	5.321299	1.791022
17 H	-4.344914	-3.352538	-1.938279	36 H	-1.119630	0.386778	1.713087
18 H	-5.061939	0.195749	1.555655	37 O	-2.126324	-2.010414	-0.850012
19 N	-2.379068	-0.426108	0.705713				

Table S23: Optimized structure (Cartesian coordinates, Å) of **9·AcOH(a)**.

	X	Y	Z		X	Y	Z
1 C	1.654460	0.840129	-0.986520	20 C	-1.344711	-1.186160	-0.162252
2 C	2.747128	-0.101213	-0.903249	21 C	0.105608	-0.864412	-0.120651
3 C	2.942565	-0.952984	0.145912	22 H	0.631815	-1.553195	-0.790964
4 C	0.737860	-0.970438	1.253884	23 H	-1.869881	2.148072	0.858882
5 C	2.090949	-1.035507	1.311342	24 Cl	1.975631	2.450693	-1.555519
6 C	0.400627	0.538230	-0.588091	25 Cl	3.846145	-0.094137	-2.245670
7 O	-0.627969	1.387330	-0.502791	26 Cl	4.306920	-2.025120	0.135519
8 O	-0.007210	-0.911953	2.349693	27 Cl	2.842821	-1.184203	2.866625
9 C	-4.643611	-1.775608	0.841205	28 H	-0.383542	2.332998	-0.640987
10 C	-5.677886	-2.093689	-0.027826	29 O	-2.269447	2.928729	1.280841
11 C	-3.389064	-1.572052	0.277215	30 O	-0.920103	3.996068	-0.153527
12 H	-6.673020	-2.260397	0.369623	31 C	-1.748341	4.026884	0.733016
13 C	-5.472224	-2.205335	-1.409938	32 C	-2.297245	5.278987	1.333433
14 C	-3.209464	-1.688773	-1.099279	33 H	-2.075842	5.297431	2.403420
15 H	-6.310991	-2.455570	-2.050177	34 H	-1.857483	6.145204	0.844364
16 C	-4.221845	-2.004199	-1.983405	35 H	-3.384394	5.293359	1.225819
17 H	-4.051627	-2.087884	-3.049756	36 H	-0.953857	-1.032346	2.103848
18 H	-4.799737	-1.688248	1.910000	37 O	-1.890572	-1.443345	-1.368195
19 N	-2.159095	-1.242883	0.837537				

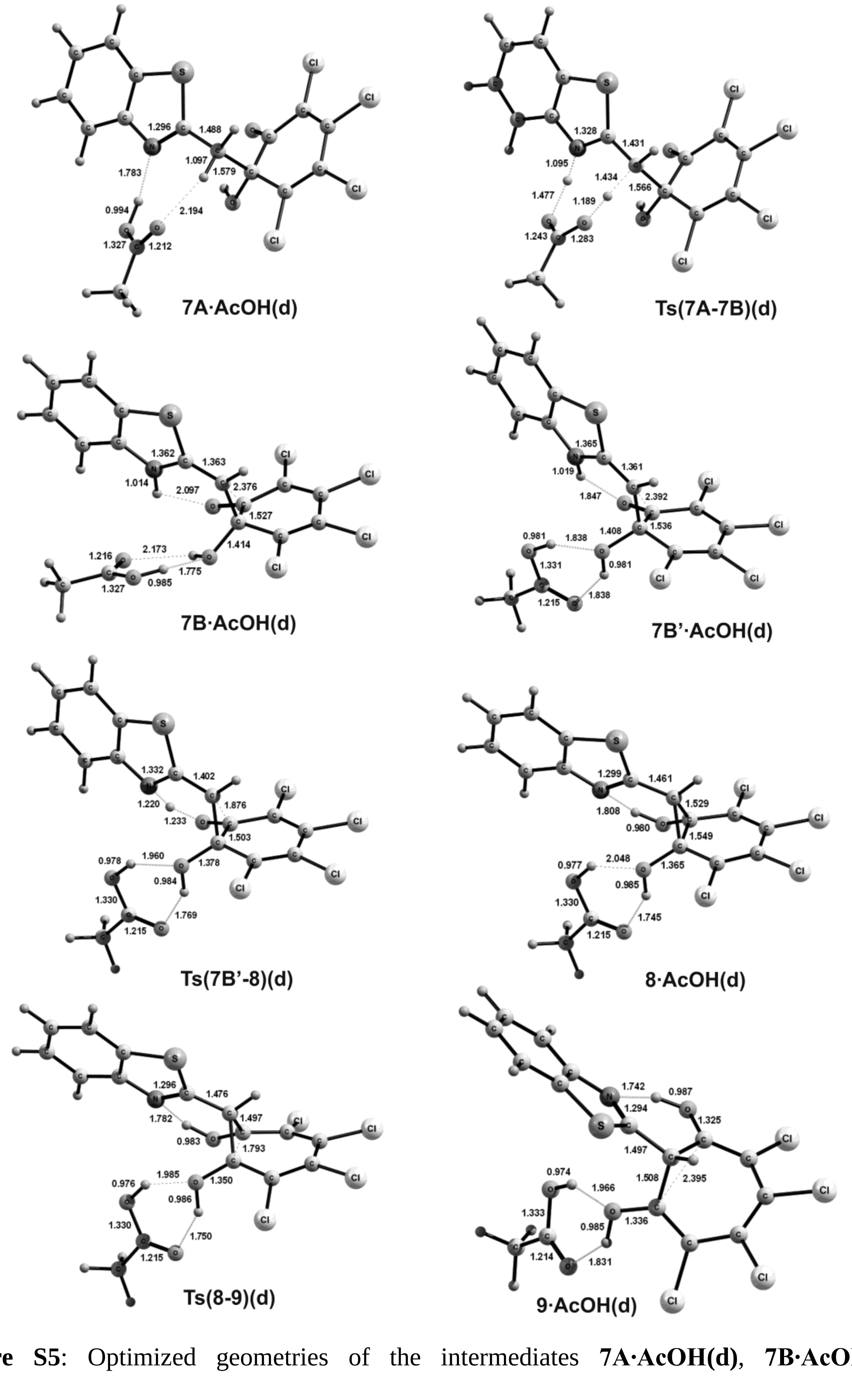

Figure S5: Optimized geometries of the intermediates **7A·AcOH(d)**, **7B·AcOH(d)**, **7B'·AcOH(d)**, **8·AcOH(d)**, **9·AcOH(d)** and transition states **Ts(7A-7B)(d)**, **Ts(7B'-8)(d)**,

Ts(8-9)(d) calculated using the PBE0/6-311+G** method in the gas phase. The bond lengths are given in angstroms.

Table S24: Optimized structure (Cartesian coordinates, Å) of **7A·AcOH(d)**.

	X	Y	Z		X	Y	Z
1 C	2.083962	1.198975	0.280383	20 C	-1.318450	-0.283986	-0.600459
2 C	3.166073	0.480966	-0.081705	21 C	-0.023225	0.437117	-0.733022
3 C	3.138238	-0.983731	-0.024166	22 H	0.597124	-0.027053	-1.503845
4 C	0.984481	-0.915307	1.107774	23 H	-0.238957	1.475077	-1.014400
5 C	2.095710	-1.662793	0.524494	24 Cl	2.054906	2.900485	0.243898
6 C	0.785363	0.519740	0.621143	25 Cl	4.612929	1.254755	-0.597597
7 O	0.082146	1.237249	1.574976	26 Cl	4.485802	-1.838844	-0.637090
8 O	0.186929	-1.377167	1.891855	27 Cl	2.077774	-3.362576	0.718802
9 C	-4.694058	-0.432055	0.493238	28 H	-0.427622	0.585550	2.079133
10 C	-5.647596	-1.430249	0.417053	29 O	-1.381331	3.344649	-1.127427
11 C	-3.424208	-0.676879	-0.036049	30 O	-2.840513	2.892010	0.509159
12 H	-6.637542	-1.257139	0.824911	31 C	-2.169457	3.716115	-0.285203
13 C	-5.356006	-2.664409	-0.179272	32 C	-2.493655	5.155700	-0.014358
14 C	-3.143027	-1.919962	-0.633673	33 H	-2.004088	5.790621	-0.750140
15 H	-6.122108	-3.430952	-0.224476	34 H	-3.574772	5.309388	-0.037520
16 C	-4.104774	-2.923839	-0.713335	35 H	-2.148797	5.419038	0.989279
17 H	-3.882439	-3.879718	-1.173723	36 H	-2.584537	1.951239	0.313064
18 H	-4.909444	0.527378	0.950696	37 S	-1.502731	-1.916176	-1.200450
19 N	-2.373022	0.216302	-0.038222				

Table S25: Optimized structure (Cartesian coordinates, Å) of **Ts(7A-7B)(d)**.

	X	Y	Z		X	Y	Z
1 C	2.192446	1.080229	0.254007	20 C	-1.300254	-0.163020	-0.537938
2 C	3.156690	0.209125	-0.104825	21 C	-0.027619	0.481416	-0.656629
3 C	2.928193	-1.235565	-0.008814	22 H	0.565568	0.071852	-1.474265
4 C	0.856339	-0.826101	1.199854	23 H	-0.421333	1.837188	-0.907782
5 C	1.819273	-1.742524	0.588025	24 Cl	2.415623	2.767198	0.189432
6 C	0.822033	0.599830	0.652912	25 Cl	4.691467	0.759265	-0.657374
7 O	0.241652	1.431815	1.601154	26 Cl	4.116776	-2.287951	-0.648564
8 O	0.049215	-1.151415	2.045032	27 Cl	1.556235	-3.418864	0.819015
9 C	-4.666775	-0.114972	0.782090	28 H	-0.199107	0.841659	2.232009
10 C	-5.755778	-0.939294	0.551186	29 O	-0.725601	2.974073	-1.077354
11 C	-3.473938	-0.407076	0.126065	30 O	-2.294075	2.839135	0.522789
12 H	-6.695040	-0.731892	1.051893	31 C	-1.575628	3.482296	-0.261959
13 C	-5.665432	-2.032805	-0.315916	32 C	-1.662139	4.982129	-0.283897
14 C	-3.386197	-1.500225	-0.745239	33 H	-1.572046	5.355502	-1.304717
15 H	-6.532643	-2.663960	-0.475367	34 H	-2.590217	5.319243	0.175602
16 C	-4.481740	-2.323252	-0.978180	35 H	-0.818497	5.378110	0.289819
17 H	-4.412966	-3.168941	-1.652761	36 H	-2.257745	1.363039	0.490668
18 H	-4.729107	0.738095	1.448239	37 O	-1.774525	-1.591586	-1.410895
19 N	-2.293225	0.305923	0.208700				

Table S26: Optimized structure (Cartesian coordinates, Å) of **7B·AcOH(d)**.

	X	Y	Z		X	Y	Z
1 C	2.257691	1.178512	-0.478771	20 C	-1.103677	-0.435485	-0.922249
2 C	3.352104	0.400171	-0.387278	21 C	0.152459	0.081842	-1.031882
3 C	3.299196	-0.877533	0.326233	22 H	0.645872	-0.010096	-1.990926
4 C	1.054449	-0.351416	1.123066	23 H	-1.095833	2.848739	0.139103
5 C	2.191133	-1.261173	1.010380	24 Cl	2.246855	2.653457	-1.342027
6 C	0.926144	0.754336	0.077697	25 Cl	4.838528	0.875229	-1.112290
7 O	0.280697	1.862951	0.673049	26 Cl	4.679734	-1.887212	0.284047
8 O	0.182894	-0.460475	1.967117	27 Cl	2.109145	-2.708169	1.920575
9 C	-4.218505	-1.089958	0.882006	28 H	-0.173001	1.551191	1.477563
10 C	-5.412287	-1.681119	0.484346	29 O	-1.958550	3.322794	0.167972
11 C	-3.193302	-0.988533	-0.050347	30 O	-2.246394	1.999226	1.948660
12 H	-6.222512	-1.770939	1.199729	31 C	-2.654379	2.867531	1.201716
13 C	-5.582533	-2.159094	-0.813459	32 C	-3.987809	3.533558	1.334385
14 C	-3.364471	-1.467151	-1.353088	33 H	-4.586647	3.326420	0.443653
15 H	-6.521329	-2.618265	-1.102178	34 H	-4.499446	3.165921	2.221253
16 C	-4.555857	-2.054338	-1.747627	35 H	-3.856898	4.616491	1.392968
17 H	-4.684921	-2.425256	-2.758267	36 H	-1.659941	-0.062617	1.048060
18 H	-4.080873	-0.714036	1.889924	37 O	-1.908891	-1.201947	-2.296549
19 N	-1.945142	-0.434210	0.149078				

Table S27: Optimized structure (Cartesian coordinates, Å) of **7B'·AcOH(d)**.

	X	Y	Z		X	Y	Z
1 C	2.141225	0.030256	-1.248699	20 C	-1.409286	-1.056003	-0.731235
2 C	3.163996	-0.472796	-0.532182	21 C	-0.091104	-0.920882	-1.043215
3 C	2.995199	-0.782077	0.887759	22 H	0.366254	-1.694273	-1.645601
4 C	0.764024	0.233502	0.868981	23 H	-1.293773	2.437022	-0.704338
5 C	1.845021	-0.485581	1.544303	24 Cl	2.276358	0.360171	-2.921038
6 C	0.763924	0.259349	-0.667002	25 Cl	4.692290	-0.778503	-1.261989
7 O	0.200883	1.459865	-1.139132	26 Cl	4.292260	-1.533597	1.712867
8 O	-0.149619	0.756026	1.485037	27 Cl	1.634942	-0.774771	3.219363
9 C	-4.494566	0.039309	0.901471	28 H	0.651885	2.240741	-0.753373
10 C	-5.770872	-0.510594	0.932992	29 O	-1.755499	3.215578	-0.325990
11 C	-3.511355	-0.616150	0.170954	30 O	0.355051	3.919455	-0.066443
12 H	-6.548980	-0.011231	1.499895	31 C	-0.838911	4.095202	0.070899
13 C	-6.064214	-1.687368	0.247330	32 C	-1.441762	5.307508	0.703906
14 C	-3.805789	-1.799957	-0.513195	33 H	-1.934494	5.017273	1.635816
15 H	-7.066571	-2.099097	0.283009	34 H	-0.664809	6.040010	0.911576
16 C	-5.079823	-2.344006	-0.485619	35 H	-2.204954	5.732431	0.048593
17 H	-5.303950	-3.261482	-1.018202	36 H	-1.746852	0.475413	0.600945
18 H	-4.265630	0.959201	1.428366	37 O	-2.377579	-2.404411	-1.335164
19 N	-2.196724	-0.222456	0.009576				

Table S28: Optimized structure (Cartesian coordinates, Å) of **Ts(7B'-8)(d)**.

	X	Y	Z		X	Y	Z
1 C	2.231805	0.446789	-1.155622	20 C	-1.284410	-0.993404	-0.794199
2 C	3.221152	-0.202392	-0.504728	21 C	0.107415	-0.845267	-0.878525
3 C	2.968672	-0.882100	0.761688	22 H	0.692924	-1.656919	-1.290548
4 C	0.649117	-0.073644	0.742899	23 H	-1.578489	2.295641	-0.257707
5 C	1.743507	-0.833607	1.341980	24 Cl	2.484251	1.245099	-2.650165
6 C	0.828653	0.465400	-0.648402	25 Cl	4.812435	-0.253840	-1.158446
7 O	0.102553	1.589330	-0.976255	26 Cl	4.250436	-1.756334	1.497222
8 O	-0.330106	0.329673	1.473080	27 Cl	1.413158	-1.563064	2.855692
9 C	-4.433819	0.010740	0.631535	28 H	0.416102	2.370809	-0.467496
10 C	-5.697988	-0.553992	0.561985	29 O	-2.051421	3.008247	0.217288
11 C	-3.388090	-0.617028	-0.040489	30 O	0.018277	3.841637	0.432186
12 H	-6.522908	-0.079116	1.081888	31 C	-1.174623	3.908730	0.652169
13 C	-5.927741	-1.720473	-0.171974	32 C	-1.818289	4.999061	1.445645
14 C	-3.624535	-1.790000	-0.772676	33 H	-2.185356	4.582796	2.387955
15 H	-6.926141	-2.141608	-0.214815	34 H	-1.092226	5.782581	1.651227
16 C	-4.893124	-2.350363	-0.850559	35 H	-2.678292	5.401468	0.906800
17 H	-5.071337	-3.255831	-1.419534	36 H	-1.364674	0.247419	0.807239
18 H	-4.248585	0.923432	1.186773	37 O	-2.138103	-2.346248	-1.507615
19 N	-2.073823	-0.187736	-0.085406				

Table S29: Optimized structure (Cartesian coordinates, Å) of **8·AcOH(d)**.

	X	Y	Z		X	Y	Z
1 C	2.052939	0.180139	-1.335063	20 C	-1.350037	-1.138704	-0.185577
2 C	3.149922	-0.313334	-0.715153	21 C	0.104487	-1.001418	-0.160497
3 C	3.130710	-0.651615	0.704982	22 H	0.663011	-1.904803	-0.381017
4 C	0.739285	-0.061244	0.864700	23 H	-1.743522	2.051477	-0.132474
5 C	2.018204	-0.481219	1.456422	24 Cl	2.082120	0.707854	-2.970291
6 C	0.760963	0.293332	-0.643445	25 Cl	4.619073	-0.527679	-1.587502
7 O	-0.105830	1.256983	-1.071318	26 Cl	4.578414	-1.248305	1.421248
8 O	-0.055326	0.677663	1.689985	27 Cl	2.006798	-0.751806	3.150649
9 C	-4.611353	-0.010752	0.659589	28 H	0.199602	2.157793	-0.816560
10 C	-5.863800	-0.551751	0.430940	29 O	-2.154228	2.867240	0.215620
11 C	-3.488981	-0.700255	0.196151	30 O	-0.154588	3.769890	-0.251686
12 H	-6.745488	-0.028597	0.784890	31 C	-1.282180	3.870572	0.188759
13 C	-6.013006	-1.764689	-0.253049	32 C	-1.842227	5.128802	0.768526
14 C	-3.650808	-1.917174	-0.492277	33 H	-2.002014	4.986313	1.840837
15 H	-7.006293	-2.167180	-0.419816	34 H	-1.146899	5.949651	0.607435
16 C	-4.910962	-2.461097	-0.723067	35 H	-2.811922	5.351683	0.318904
17 H	-5.029803	-3.399111	-1.253363	36 H	-0.990795	0.500166	1.456239
18 H	-4.482580	0.931067	1.181216	37 O	-2.088328	-2.532355	-0.944266
19 N	-2.179728	-0.294803	0.349962				

Table S30: Optimized structure (Cartesian coordinates, Å) of **Ts(8-9)(d)**.

	X	Y	Z		X	Y	Z
1 C	1.963572	0.178804	-1.323042	20 C	-1.378746	-1.136084	-0.113094
2 C	3.006357	-0.509514	-0.745786	21 C	0.086861	-0.967623	-0.075848
3 C	3.019321	-0.844554	0.643851	22 H	0.642460	-1.874907	-0.303128
4 C	0.689953	-0.159132	1.030618	23 H	-1.642912	2.156644	-0.040038
5 C	1.995768	-0.499671	1.497557	24 Cl	2.169684	1.057606	-2.799183
6 C	0.679421	0.253737	-0.713770	25 Cl	4.407956	-0.853105	-1.696519
7 O	-0.208735	1.216592	-1.039441	26 Cl	4.440976	-1.576118	1.300079
8 O	-0.086334	0.614868	1.807261	27 Cl	2.242825	-0.405098	3.202974
9 C	-4.647357	-0.188500	0.899751	28 H	0.176662	2.122764	-0.987433
10 C	-5.894455	-0.688584	0.573077	29 O	-1.989097	3.000573	0.307266
11 C	-3.518705	-0.780502	0.329037	30 O	-0.053269	3.789138	-0.503169
12 H	-6.781453	-0.240228	1.007100	31 C	-1.100546	3.965952	0.087399
13 C	-6.031822	-1.765930	-0.311875	32 C	-1.530159	5.279614	0.653322
14 C	-3.668260	-1.860939	-0.560611	33 H	-1.525898	5.213853	1.744868
15 H	-7.021704	-2.138862	-0.551467	34 H	-0.845965	6.061214	0.330611
16 C	-4.923878	-2.364988	-0.888544	35 H	-2.551531	5.508025	0.342513
17 H	-5.034202	-3.197851	-1.573539	36 H	-1.027472	0.409745	1.609903
18 H	-4.528390	0.647354	1.579989	37 O	-2.100138	-2.381090	-1.101861
19 N	-2.212337	-0.401147	0.553977				

Table S31: Optimized structure (Cartesian coordinates, Å) of **9·AcOH(d)**.

	X	Y	Z		X	Y	Z
1 C	1.865759	0.852085	-0.936569	20 C	-1.239132	-1.099124	-0.283571
2 C	2.922216	-0.124266	-0.807038	21 C	0.223021	-0.804710	-0.155705
3 C	3.040886	-0.985871	0.246234	22 H	0.784630	-1.498888	-0.789721
4 C	0.787348	-0.922493	1.248500	23 H	-1.756019	2.202823	0.689240
5 C	2.133461	-1.041595	1.369285	24 Cl	2.266451	2.455499	-1.476715
6 C	0.584761	0.591209	-0.596721	25 Cl	4.084009	-0.147293	-2.096242
7 O	-0.413481	1.477483	-0.550820	26 Cl	4.372246	-2.098342	0.296368
8 O	-0.006371	-0.828074	2.305145	27 Cl	2.805531	-1.212035	2.959149
9 C	-4.503427	-1.248575	1.105503	28 H	-0.127191	2.414366	-0.656382
10 C	-5.722683	-1.598623	0.555616	29 O	-2.188089	2.972939	1.100154
11 C	-3.368575	-1.286905	0.292960	30 O	-0.697057	4.079755	-0.153896
12 H	-6.614721	-1.574421	1.171849	31 C	-1.606979	4.084759	0.649047
13 C	-5.825373	-1.984118	-0.787561	32 C	-2.200244	5.319355	1.244657
14 C	-3.482466	-1.675519	-1.054716	33 H	-2.064216	5.301170	2.328924
15 H	-6.794163	-2.253849	-1.193825	34 H	-1.719847	6.199105	0.822480
16 C	-4.710631	-2.027755	-1.608384	35 H	-3.275248	5.343243	1.051708
17 H	-4.794366	-2.326883	-2.646887	36 H	-0.943500	-0.846905	1.995604
18 H	-4.412023	-0.946494	2.142706	37 O	-1.913178	-1.632696	-1.800735
19 N	-2.085740	-0.965310	0.685524				

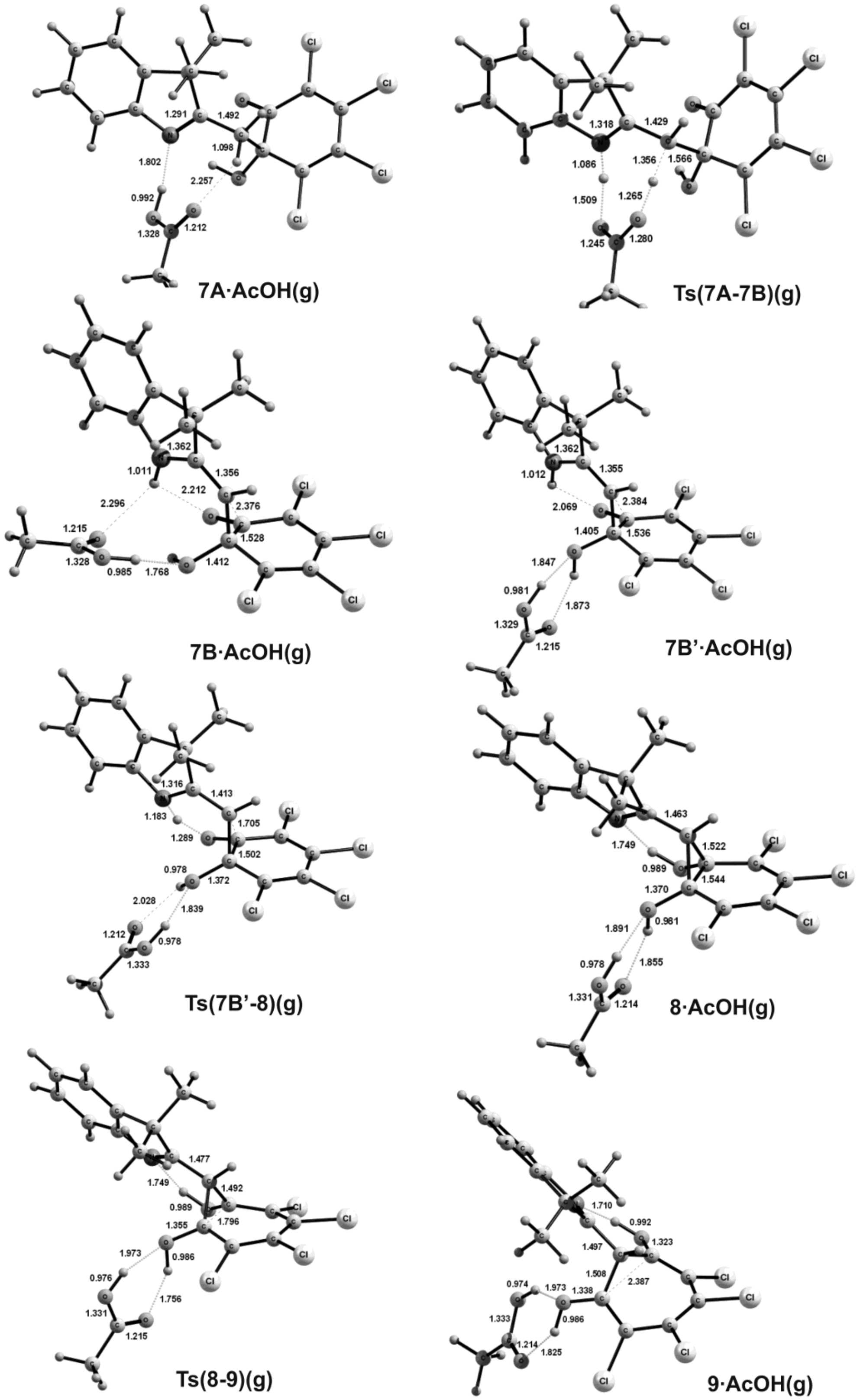

Figure S6: Optimized geometries of the intermediates **7A·AcOH(g), 7B·AcOH(g), 7B’·AcOH(g), 8·AcOH(g), 9·AcOH(g)** and transition states **Ts(7A-7B)(g)**, **Ts(7B’-8)(g)**,

Ts(8-9)(g) calculated using the PBE0/6-311+G** method in the gas phase. The bond lengths are given in angstroms.

Table S32: Optimized structure (Cartesian coordinates, Å) of **7A·AcOH(g)**.

	X	Y	Z		X	Y	Z
1 C	-2.072760	1.264446	-0.265555	24 H	2.573994	-0.242178	2.837933
2 C	-3.213050	0.631621	0.073114	25 H	0.962398	-0.931282	3.118663
3 C	-3.324003	-0.824634	-0.051187	26 C	0.942969	-2.723115	1.014980
4 C	-1.128422	-0.928772	-1.126885	27 H	1.363891	-3.570001	1.564306
5 C	-2.337091	-1.574028	-0.606016	28 H	-0.060293	-2.539925	1.413148
6 C	-0.835291	0.507258	-0.665408	29 H	0.855446	-3.001463	-0.036619
7 O	-0.181689	1.204066	-1.669595	30 C	0.039810	0.380325	0.634960
8 O	-0.327442	-1.491967	-1.834390	31 H	-0.532787	-0.142998	1.405679
9 C	4.520197	-0.524059	-1.120099	32 H	0.236522	1.404919	0.976283
10 C	5.532004	-1.456748	-0.905706	33 Cl	-1.903062	2.957552	-0.149022
11 C	3.362617	-0.652519	-0.369041	34 Cl	-4.578173	1.509874	0.642765
12 H	6.451434	-1.389559	-1.477688	35 Cl	-4.762286	-1.571484	0.497521
13 C	5.380322	-2.475681	0.032576	36 Cl	-2.479699	-3.261882	-0.861856
14 C	3.200312	-1.668841	0.573420	37 H	0.648606	0.739740	-1.848221
15 H	6.183189	-3.190531	0.179427	38 O	1.675727	3.135669	1.145642
16 C	4.209730	-2.589757	0.784510	39 O	2.730682	2.893200	-0.813677
17 H	4.103989	-3.388639	1.512730	40 C	2.289705	3.608867	0.214551
18 H	4.628380	0.274263	-1.846314	41 C	2.635172	5.062380	0.082638
19 C	1.842071	-1.496759	1.201044	42 H	2.320436	5.600221	0.974661
20 N	2.223351	0.180166	-0.419548	43 H	3.710136	5.179588	-0.072359
21 C	1.357363	-0.284559	0.417345	44 H	2.133417	5.474628	-0.796842
22 C	1.950055	-1.126995	2.690750	45 H	2.468152	1.944128	-0.693730
23 H	2.395880	-1.959408	3.242064				

Table S33: Optimized structure (Cartesian coordinates, Å) of **Ts(7A-7B)(g)**.

	X	Y	Z		X	Y	Z
1 C	-2.174499	1.160254	-0.241193	24 H	2.480593	0.043222	2.838929
2 C	-3.167195	0.334863	0.147128	25 H	0.933651	-0.800133	3.051823
3 C	-3.041818	-1.114897	-0.026266	26 C	1.179319	-2.588104	0.961344
4 C	-0.997509	-0.794328	-1.310304	27 H	1.661144	-3.396443	1.518313
5 C	-2.004544	-1.666609	-0.707575	28 H	0.150618	-2.496933	1.321238
6 C	-0.855897	0.616479	-0.730384	29 H	1.156260	-2.866616	-0.094584
7 O	-0.297761	1.439992	-1.696757	30 C	0.061040	0.436254	0.526307
8 O	-0.242588	-1.139663	-2.194431	31 H	-0.494363	-0.019962	1.346469
9 C	4.652133	-0.117725	-1.041541	32 H	0.440538	1.707123	0.807022
10 C	5.735253	-0.937538	-0.733131	33 Cl	-2.300225	2.852545	-0.118890
11 C	3.475344	-0.344918	-0.349555	34 Cl	-4.627611	0.953997	0.816309
12 H	6.676098	-0.793620	-1.253490	35 Cl	-4.272467	-2.115010	0.616498
13 C	5.628948	-1.938024	0.230164	36 Cl	-1.900711	-3.340872	-1.059739
14 C	3.350252	-1.336664	0.619753	37 H	0.107389	0.847032	-2.348050
15 H	6.487072	-2.565023	0.447088	38 O	0.822262	2.896199	1.010540
16 C	4.432748	-2.142908	0.919737	39 O	2.210420	2.882331	-0.755795
17 H	4.362434	-2.921742	1.673146	40 C	1.578188	3.461781	0.146414
18 H	4.725764	0.666722	-1.786225	41 C	1.670463	4.961175	0.255788
19 C	1.949686	-1.282198	1.182437	42 H	0.780220	5.390762	-0.214160
20 N	2.254090	0.339907	-0.469872	43 H	1.674414	5.270206	1.301829
21 C	1.352672	-0.150962	0.356585	44 H	2.553539	5.330765	-0.264146
22 C	1.953804	-0.899002	2.671069	45 H	2.178520	1.373932	-0.791692
23 H	2.455704	-1.682304	3.245449				

Table S34: Optimized structure (Cartesian coordinates, Å) of **7B·AcOH(g)**.

	X	Y	Z		X	Y	Z
1 C	-2.257277	1.197662	0.529963	24 H	2.524487	0.435764	2.830377
2 C	-3.357493	0.431662	0.412611	25 H	1.069108	-0.342791	3.475826
3 C	-3.343536	-0.765891	-0.432713	26 C	1.163191	-2.643321	1.949223
4 C	-1.148979	-0.141533	-1.293654	27 H	1.753180	-3.256466	2.636865
5 C	-2.277330	-1.068768	-1.215336	28 H	0.177449	-2.481388	2.394745
6 C	-0.955686	0.823279	-0.125288	29 H	1.033170	-3.201350	1.018797
7 O	-0.300389	1.979480	-0.600930	30 C	-0.148562	0.000779	0.857275
8 O	-0.337356	-0.136808	-2.198448	31 H	-0.629645	-0.237471	1.797965
9 C	4.225843	-0.937809	-1.132594	32 H	1.120378	2.837391	0.009557
10 C	5.377637	-1.599641	-0.710037	33 Cl	-2.199165	2.577683	1.535924
11 C	3.150875	-0.910676	-0.257398	34 Cl	-4.806520	0.826239	1.253293
12 H	6.236504	-1.641168	-1.371976	35 Cl	-4.719571	-1.783369	-0.418144
13 C	5.444517	-2.206930	0.540274	36 Cl	-2.244882	-2.411337	-2.276039
14 C	3.199073	-1.513497	0.998648	37 H	0.077473	1.773341	-1.475034
15 H	6.352230	-2.716391	0.844582	38 O	1.989280	3.300776	0.033732
16 C	4.347590	-2.164990	1.404789	39 O	2.188224	2.306395	-1.960110
17 H	4.403530	-2.640266	2.379923	40 C	2.638554	3.026983	-1.091410
18 H	4.170917	-0.464082	-2.106725	41 C	3.980246	3.687078	-1.156122
19 C	1.871953	-1.306529	1.697371	42 H	3.888201	4.750914	-0.928159
20 N	1.905210	-0.319733	-0.433299	43 H	4.632037	3.245252	-0.397185
21 C	1.102437	-0.481827	0.655124	44 H	4.416608	3.543260	-2.142357
22 C	2.039322	-0.527269	3.005765	45 H	1.644156	0.185530	-1.269297
23 H	2.653768	-1.099959	3.706702				

Table S35: Optimized structure (Cartesian coordinates, Å) of **7B'·AcOH(g)**.

	X	Y	Z		X	Y	Z
1 C	-1.989954	0.196934	0.751051	24 H	2.988847	1.095325	2.637378
2 C	-2.820503	-0.851804	0.610356	25 H	1.961289	-0.145432	3.375980
3 C	-2.508131	-1.925533	-0.337674	26 C	2.683516	-2.251169	1.734650
4 C	-0.634324	-0.614484	-1.224726	27 H	3.523871	-2.627940	2.325234
5 C	-1.447297	-1.838768	-1.176407	28 H	1.760839	-2.452762	2.286471
6 C	-0.678824	0.303625	0.006304	29 H	2.650224	-2.805119	0.793518
7 O	-0.375037	1.621403	-0.373461	30 C	0.415125	-0.252789	0.885128
8 O	0.095932	-0.353224	-2.157297	31 H	0.116606	-0.697579	1.825716
9 C	4.598967	0.503977	-1.527456	32 H	-0.537783	3.333354	0.301657
10 C	5.946794	0.261952	-1.266982	33 Cl	-2.282915	1.444643	1.887792
11 C	3.683776	0.135004	-0.553405	34 Cl	-4.261865	-0.976087	1.543086
12 H	6.686766	0.538942	-2.010734	35 Cl	-3.523940	-3.304242	-0.362308
13 C	6.357894	-0.325687	-0.074788	36 Cl	-1.084590	-3.038348	-2.342227
14 C	4.078168	-0.460186	0.645343	37 H	-0.969774	1.940654	-1.082228
15 H	7.412793	-0.502787	0.103738	38 O	-0.804885	4.274547	0.234558
16 C	5.417354	-0.691135	0.891791	39 O	-1.803535	3.526792	-1.626465
17 H	5.741101	-1.152829	1.820227	40 C	-1.547622	4.431424	-0.856847
18 H	4.280918	0.962186	-2.457707	41 C	-2.030274	5.836857	-1.023602
19 C	2.849918	-0.748042	1.483960	42 H	-2.663313	6.106752	-0.174440
20 N	2.302215	0.277502	-0.557601	43 H	-1.179855	6.522520	-1.025838
21 C	1.733023	-0.224681	0.573364	44 H	-2.591802	5.926876	-1.950890
22 C	2.881706	0.021685	2.809129	45 H	1.748730	0.589339	-1.345288
23 H	3.725631	-0.314646	3.418781				

Table S36: Optimized structure (Cartesian coordinates, Å) of **Ts(7B'-8)(g)**.

	X	Y	Z		X	Y	Z
1 C	-2.137910	0.544996	0.847986	24 H	2.568255	-0.007679	2.872935
2 C	-3.103378	-0.344548	0.528662	25 H	1.318056	-1.205161	3.260064
3 C	-2.905734	-1.318082	-0.542026	26 C	1.989895	-3.081125	1.335705
4 C	-0.644123	-0.425941	-0.963615	27 H	2.713694	-3.659126	1.916767
5 C	-1.755318	-1.342274	-1.256681	28 H	0.999219	-3.255904	1.765341
6 C	-0.813357	0.506000	0.202363	29 H	1.996207	-3.456628	0.309799
7 O	-0.167049	1.712577	0.111485	30 C	0.029316	-0.728288	0.573022
8 O	0.193872	-0.088235	-1.901420	31 H	-0.468569	-1.448521	1.211303
9 C	4.491606	-0.031442	-1.190597	32 H	-0.224788	3.487471	0.590720
10 C	5.776849	-0.481815	-0.897063	33 Cl	-2.351963	1.745026	2.059351
11 C	3.464411	-0.463000	-0.368519	34 Cl	-4.603593	-0.345631	1.373352
12 H	6.606555	-0.166317	-1.520764	35 Cl	-4.174527	-2.428296	-0.886644
13 C	6.013706	-1.325893	0.185047	36 Cl	-1.513236	-2.399078	-2.580548
14 C	3.684274	-1.313017	0.716576	37 H	0.072898	1.912505	-0.815365
15 H	7.024757	-1.658880	0.393584	38 O	-0.069975	4.444940	0.461562
16 C	4.964439	-1.748969	1.003256	39 O	0.581606	3.729349	-1.559172
17 H	5.158918	-2.410362	1.842501	40 C	0.396270	4.632428	-0.772852
18 H	4.299386	0.630956	-2.027174	41 C	0.658247	6.076884	-1.065365
19 C	2.358033	-1.592920	1.386972	42 H	-0.268774	6.645894	-0.959923
20 N	2.100906	-0.137784	-0.449130	43 H	1.369505	6.477312	-0.338954
21 C	1.439851	-0.767643	0.498628	44 H	1.050038	6.183418	-2.074607
22 C	2.314175	-1.069391	2.829020	45 H	1.348460	0.045988	-1.343438
23 H	3.031131	-1.619652	3.444679				

Table S37: Optimized structure (Cartesian coordinates, Å) of **8·AcOH(g)**.

	X	Y	Z		X	Y	Z
1 C	-1.890871	0.466759	0.908809	24 H	2.373363	0.653190	2.297753
2 C	-2.930115	-0.382125	0.744525	25 H	1.222806	-0.542395	2.924890
3 C	-2.936310	-1.366981	-0.334513	26 C	2.313892	-2.777913	1.691106
4 C	-0.662903	-0.690043	-1.033309	27 H	3.030678	-3.065246	2.465344
5 C	-1.901025	-1.471169	-1.199076	28 H	1.309319	-2.963649	2.083240
6 C	-0.671500	0.357934	0.100574	29 H	2.474578	-3.421509	0.822608
7 O	0.039204	1.506916	-0.125525	30 C	0.152382	-0.934640	0.228678
8 O	-0.036007	-0.351944	-2.192951	31 H	-0.265636	-1.652004	0.927910
9 C	4.676604	-0.219507	-1.432979	32 H	0.074906	3.282999	0.521839
10 C	5.972596	-0.378903	-0.948109	33 Cl	-1.907291	1.718815	2.092699
11 C	3.629695	-0.554651	-0.588867	34 Cl	-4.294185	-0.304971	1.793228
12 H	6.815898	-0.126112	-1.582283	35 Cl	-4.318521	-2.380486	-0.504781
13 C	6.202703	-0.858513	0.339626	36 Cl	-1.936150	-2.530958	-2.546198
14 C	3.850139	-1.036186	0.704628	37 H	-0.214959	1.927171	-0.974873
15 H	7.221879	-0.972671	0.693716	38 O	0.085723	4.250264	0.376756
16 C	5.138756	-1.194008	1.179555	39 O	-0.399194	3.617166	-1.716751
17 H	5.330827	-1.568290	2.181091	40 C	-0.191029	4.493089	-0.902437
18 H	4.485426	0.152493	-2.433488	41 C	-0.214634	5.954975	-1.214256
19 C	2.504485	-1.299043	1.331995	42 H	-0.968027	6.449512	-0.596310
20 N	2.253876	-0.477070	-0.874787	43 H	0.752367	6.398877	-0.965692
21 C	1.613271	-0.885285	0.167938	44 H	-0.437031	6.103201	-2.268658
22 C	2.240768	-0.401792	2.548984	45 H	0.934952	-0.314396	-2.011189
23 H	2.936784	-0.653681	3.354058				

Table S38: Optimized structure (Cartesian coordinates, Å) of **Ts(8-9)(g)**.

	X	Y	Z		X	Y	Z
1 C	-1.832646	0.598981	0.749516	24 H	2.338271	1.063977	1.884712
2 C	-2.806750	-0.370121	0.813098	25 H	1.126780	0.078307	2.728508
3 C	-2.856434	-1.452294	-0.120011	26 C	2.189193	-2.410645	2.095325
4 C	-0.651574	-0.970208	-1.110940	27 H	2.878621	-2.521685	2.936716
5 C	-1.935015	-1.602142	-1.132064	28 H	1.170826	-2.475288	2.490081
6 C	-0.610775	0.397463	0.051808	29 H	2.352868	-3.244909	1.408649
7 O	0.147071	1.434411	-0.379247	30 C	0.105451	-0.919385	0.173578
8 O	-0.027447	-0.747777	-2.273007	31 H	-0.323211	-1.546612	0.953373
9 C	4.697758	-0.730973	-1.474070	32 H	0.589021	3.232202	0.301854
10 C	5.976672	-0.792485	-0.925696	33 Cl	-2.058569	2.153065	1.487777
11 C	3.623186	-0.830168	-0.605059	34 Cl	-4.084654	-0.202480	1.965800
12 H	6.841779	-0.718149	-1.576171	35 Cl	-4.212432	-2.523463	-0.060997
13 C	6.161834	-0.948394	0.446476	36 Cl	-2.292124	-2.539870	-2.535313
14 C	3.797690	-0.985497	0.772803	37 H	-0.340968	2.022307	-1.002746
15 H	7.168692	-0.992729	0.848170	38 O	0.619835	4.196308	0.155610
16 C	5.069739	-1.048053	1.310669	39 O	-0.625653	3.640809	-1.620747
17 H	5.227291	-1.169886	2.378384	40 C	-0.081666	4.485732	-0.938370
18 H	4.541015	-0.610975	-2.540322	41 C	-0.124296	5.951021	-1.222240
19 C	2.430369	-1.062160	1.404143	42 H	-0.622228	6.465457	-0.396349
20 N	2.255148	-0.795876	-0.940763	43 H	0.892155	6.345822	-1.286843
21 C	1.582389	-0.923007	0.148357	44 H	-0.661190	6.130379	-2.150994
22 C	2.159761	0.101018	2.368514	45 H	0.945152	-0.717403	-2.096788
23 H	2.821577	0.024935	3.235684				

Table S39: Optimized structure (Cartesian coordinates, Å) of **9·AcOH(g)**.

	X	Y	Z		X	Y	Z
1 C	1.865602	0.727325	-1.010659	24 H	-2.148721	0.203134	-2.371975
2 C	2.886673	-0.266169	-0.777513	25 H	-0.758966	-0.812651	-2.796872
3 C	3.010555	-0.972711	0.385333	26 C	-1.495976	-3.074273	-1.385839
4 C	0.794766	-0.673865	1.430466	27 H	-2.087750	-3.564822	-2.163121
5 C	2.140181	-0.832762	1.529025	28 H	-0.449138	-3.092516	-1.703287
6 C	0.589614	0.574107	-0.594121	29 H	-1.595072	-3.655003	-0.465302
7 O	-0.365317	1.509890	-0.633538	30 C	0.183659	-0.731407	0.042859
8 O	0.039606	-0.404564	2.483019	31 H	0.700647	-1.525047	-0.507375
9 C	-4.532259	-0.875942	1.356392	32 H	-1.548996	2.440825	0.641417
10 C	-5.735111	-1.296219	0.794264	33 Cl	2.310326	2.222181	-1.782102
11 C	-3.383520	-1.038072	0.599545	34 Cl	3.997487	-0.523948	-2.087429
12 H	-6.656273	-1.185160	1.356355	35 Cl	4.303489	-2.119663	0.554260
13 C	-5.772661	-1.857030	-0.480872	36 Cl	2.857217	-0.815847	3.109170
14 C	-3.408301	-1.598884	-0.679094	37 H	-0.033943	2.413649	-0.845113
15 H	-6.722995	-2.175483	-0.896205	38 O	-1.896776	3.276323	1.001969
16 C	-4.606044	-2.014435	-1.231216	39 O	-0.462275	4.150424	-0.481182
17 H	-4.649686	-2.453028	-2.223824	40 C	-1.293945	4.295704	0.391023
18 H	-4.489883	-0.436733	2.346942	41 C	-1.756768	5.620460	0.902618
19 C	-1.992697	-1.631279	-1.197971	42 H	-2.841039	5.699856	0.794973
20 N	-2.068519	-0.677919	0.963031	43 H	-1.528910	5.697999	1.968664
21 C	-1.290643	-0.983351	-0.014235	44 H	-1.262218	6.419735	0.355279
22 C	-1.809485	-0.827328	-2.491521	45 H	-0.909554	-0.422906	2.194450
23 H	-2.386426	-1.292501	-3.295555				

Fluorescence emission, fluorescence excitation and electronic absorption spectra spectra of compounds 11a-f in heptane

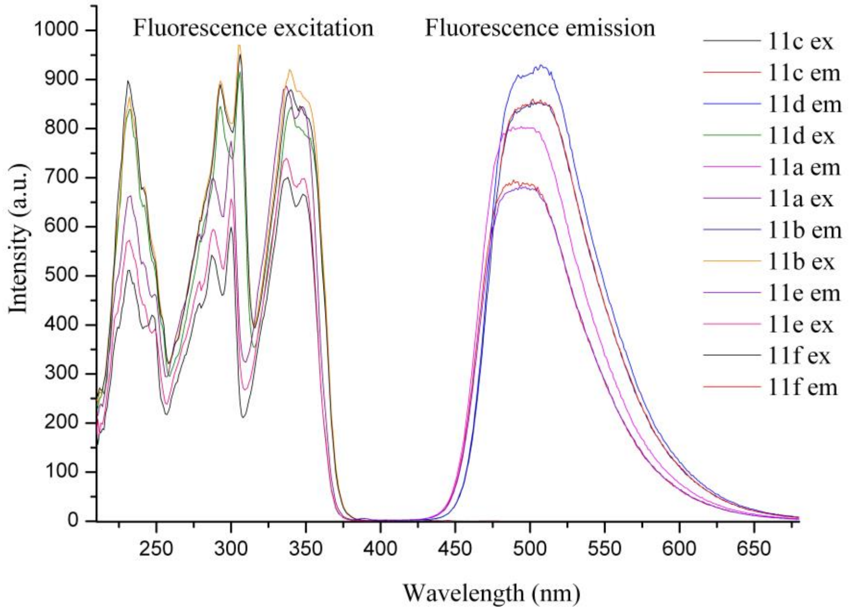

Figure S7: Fluorescence emission (λ_{ex} = 350 nm) and fluorescence excitation (λ_{obs} = 495-510 nm) spectra of compounds **11a-f** in heptane ($C = 2{\cdot}10^{-5}$ mol×l^{-1}, l = 1 cm) at 293 K.

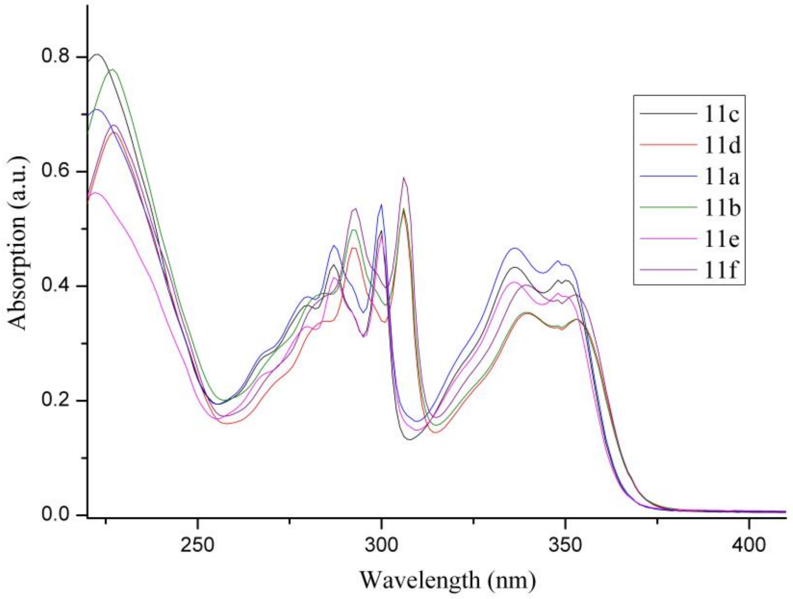

Figure S8: Electronic absorption spectra of compounds **11a-f** in heptane ($C = 2{\cdot}10^{-5}$ mol×l^{-1}, l = 1 cm) at 293 K.

References

1. Parker C.A. in *Photoluminescence of Solutions with Applications to Photochemistry and Analytical Chemistry*, Elsevier Publishing Company, Amsterdam-London-New York, NY, **1968**.

2. Krasovitskii B.M., Bolotin B.M. in *Organicheskie Lyuminofory*, Khimiya, Moscow, **1984**, 292, (in Russian).

3. Sheldrick G.M., SHELXTL v. 6.14, Structure Determination Software Suite; Bruker AXS, Madison, Wisconsin, USA, **2000**.

4. Perdew J.P., Burke K., Ernzerhof M., *Phys. Rev. Lett*., **1996**, *77*, 3865-3868.

5. *Gaussian 09*, Revision D.01; Gaussian, Wallingford, CT, 2013.

6. Tomasi J., Mennucci B., Cammi R, *Chem. Rev.*, **2005**, *105* , 2999-3093.

7. Bondareva I. O., Sayapin Yu. A., Komissarov V. N., Tkachev V. V., Shilov G. V., Aldoshin S. M., Minkin V. I., *Russ. Chem. Bull*., **2011**, *60*, 1384-1386.

8. Tupaeva I. O., Sayapin Yu. A., Bang Z. N., Komissarov V. N., Tkachev V. V., Shilov G. V., Aldoshin S. M., Minkin V. I., *Russ. Chem. Bull*., **2013**, *62*, 492-496.
