# Supplementary material for: 2-Hetaryl-1,3-tropolones based on five-membered nitrogen heterocycles: synthesis, structure and properties
Source: Beilstein J Org Chem. 2015 Nov 12;11:2179–88. doi: 10.3762/bjoc.11.236 (PMC4661002; doi:10.3762/bjoc.11.236)

Supporting Information File 2

for

2-Hetaryl-1,3-tropolones based on five-membered nitrogen heterocycles: synthesis, structure and properties

Yury A. Sayapin[1,2], Inna O. Tupaeva[2], Alexandra A. Kolodina[2], Eugeny A. Gusakov[2], Vitaly N. Komissarov[2], Igor V. Dorogan[2*], Nadezhda I. Makarova[2], Anatoly V. Metelitsa[2], Valery V. Tkachev[3], Sergey M. Aldoshin[3] and Vladimir I. Minkin[1,2].

Address: [1]Southern Scientific Center of Russian Academy of Sciences, 141 Chekhov St., 344006 Rostov on Don, Russian Federation, [2]Institute of Physical and Organic Chemistry, Southern Federal University, 194/2 Stachka St., 344090 Rostov on Don, Russian Federation and [3]Institute of Problems of Chemical Physics of Russian Academy of Sciences, 1 Akad. Semjonov N.N. Ave., 142432 Chernogolovka, Moscow region, Russian Federation

Email: Igor V. Dorogan - id@ipoc.sfedu.ru

* Corresponding author

Copies of ^{1}H and ^{13}C NMR spectra of 5a–g, 6a–g, 11a–g, 13

5a

^{1}H NMR of **5a** ($CDCl_3$)

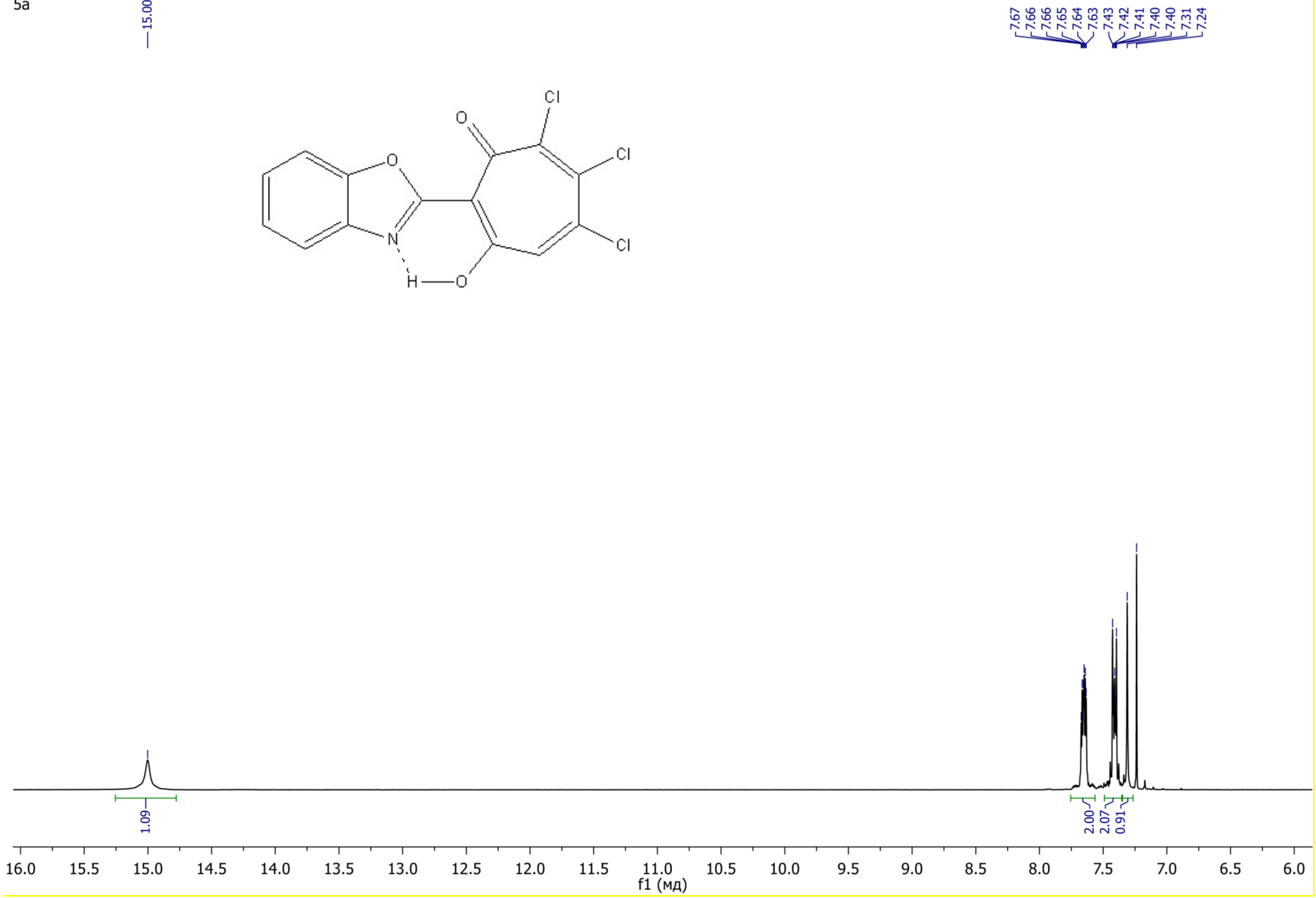

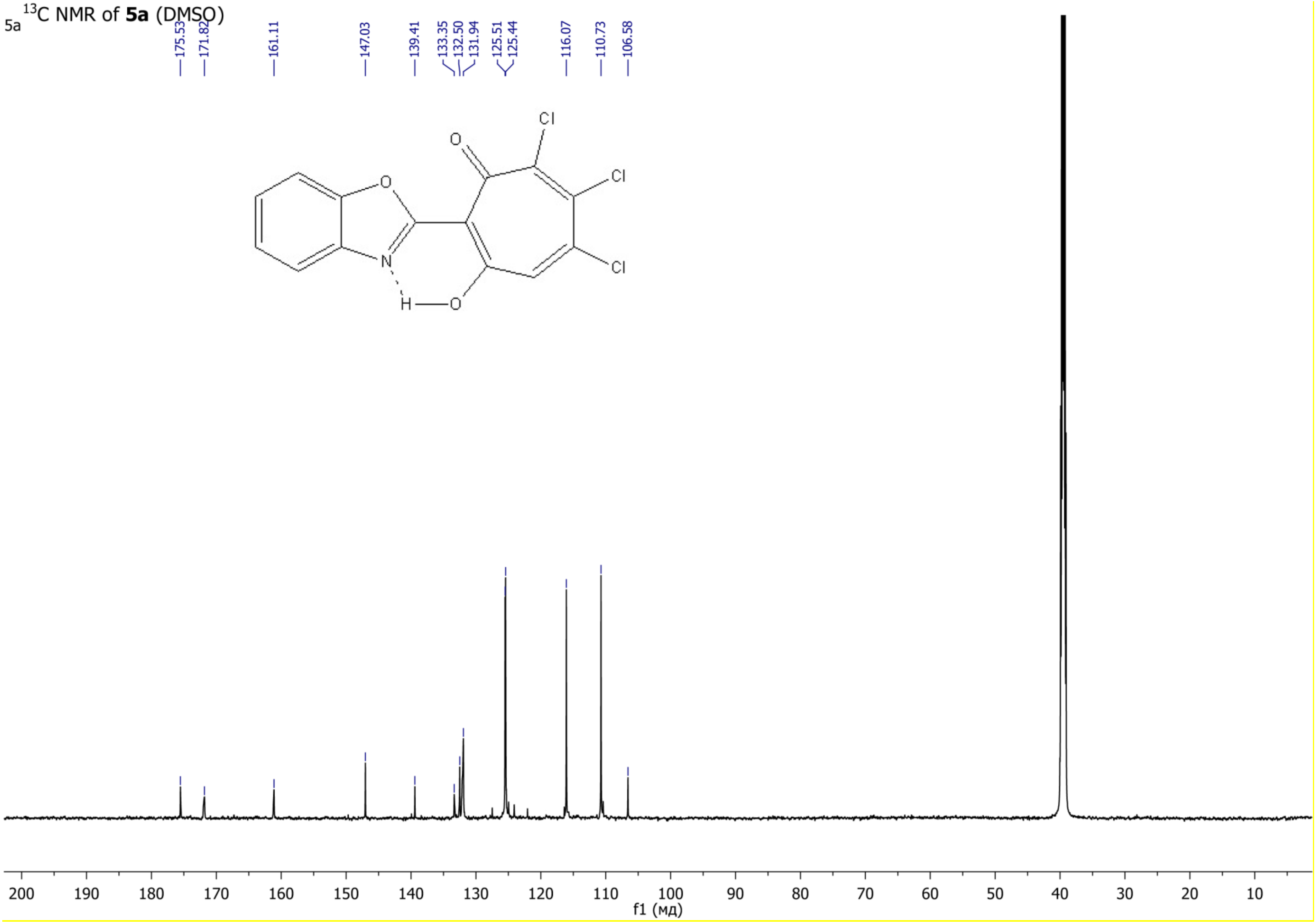
5a
13C NMR of 5a (DMSO)
175.53
171.82
161.11
147.03
139.41
133.35
132.50
131.94
125.51
125.44
116.07
110.73
106.58
O
Cl
Cl
Cl
O
N
H
O
200 190 180 170 160 150 140 130 120 110 100 90 80 70 60 50 40 30 20 10
f1 (мд)

5b

^{1}H NMR of **5b** ($CDCl_3$)

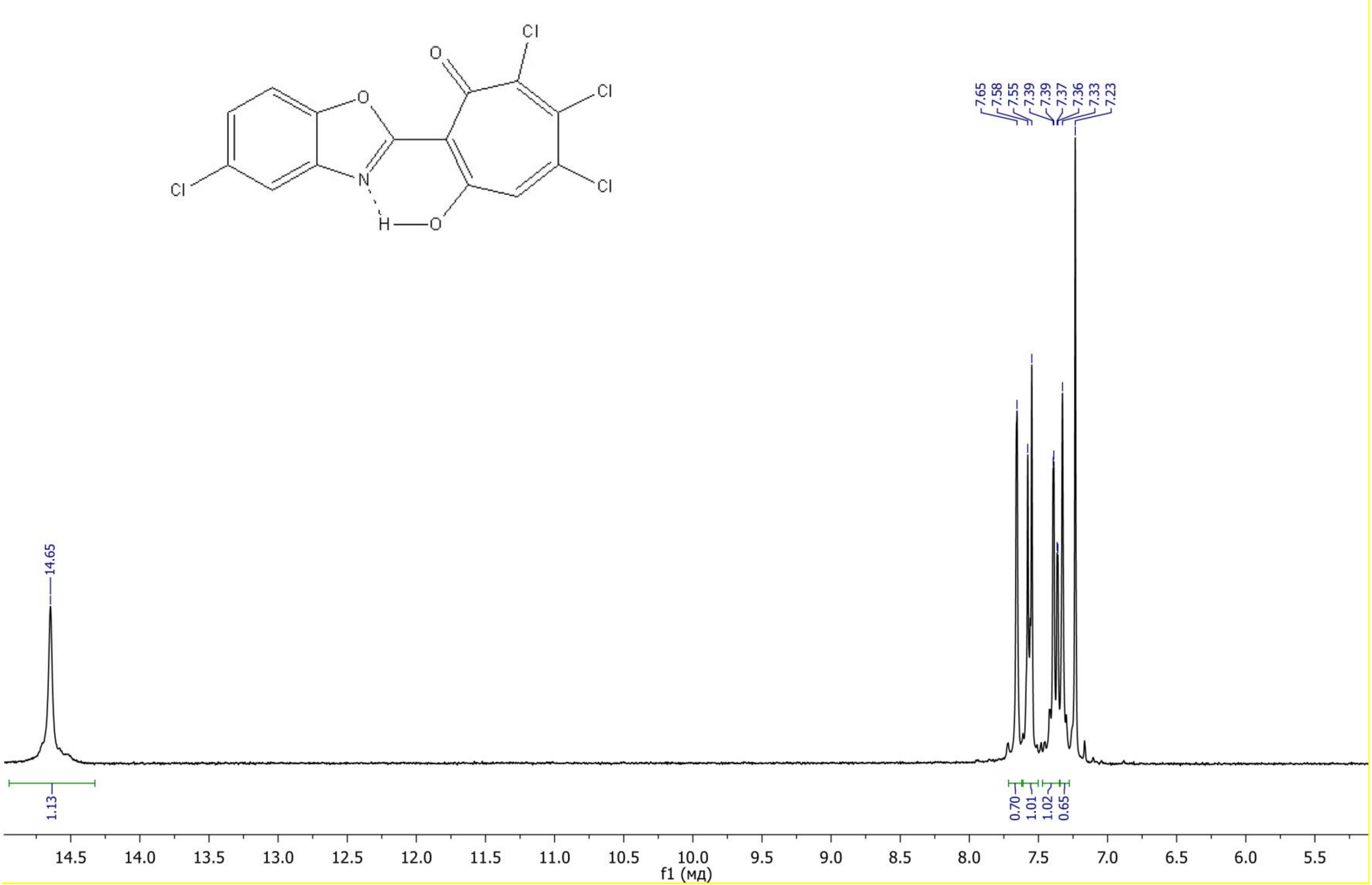

5b ^{13}C NMR of **5b** (DMSO_d$_6$)

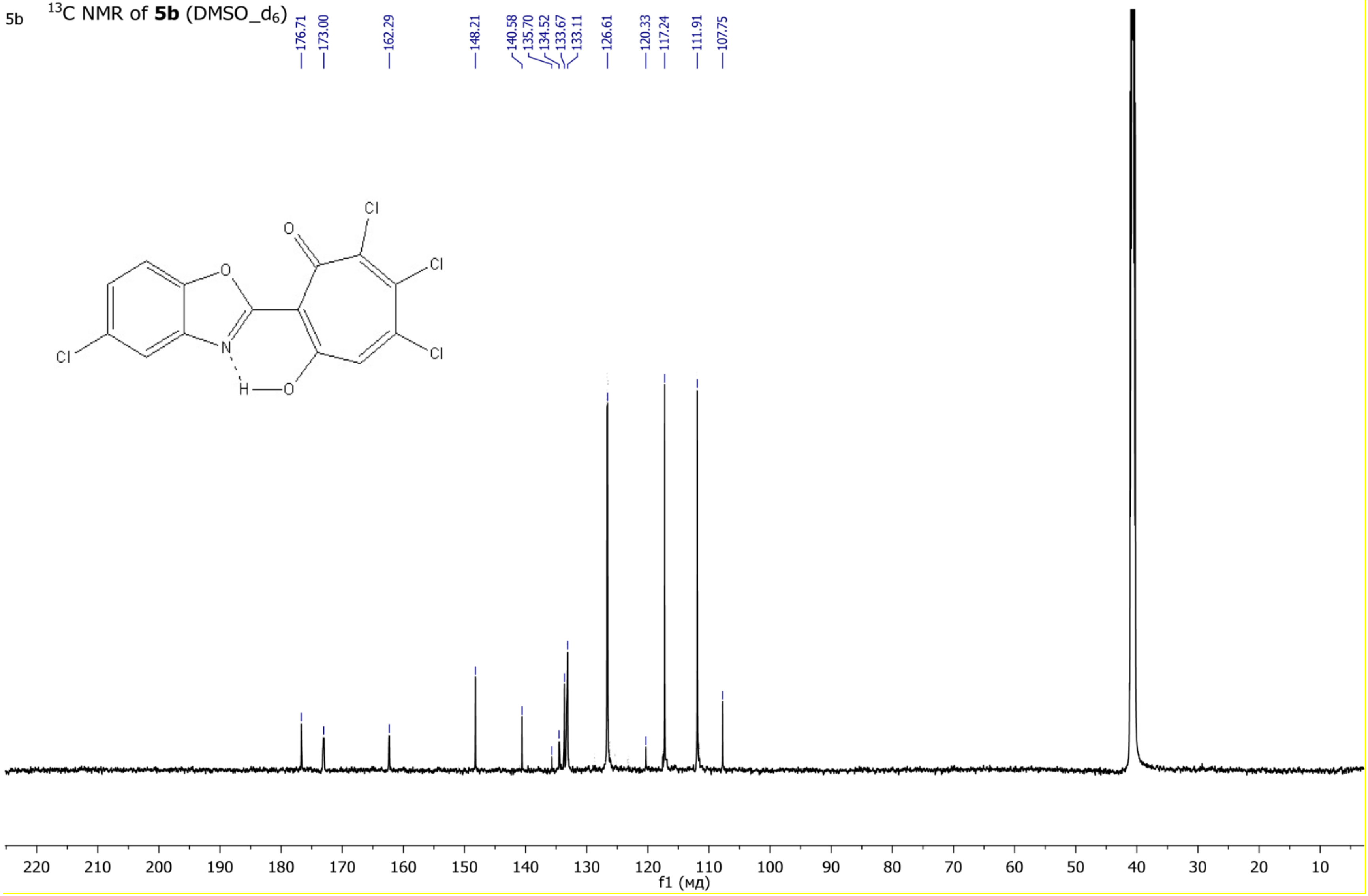

5c

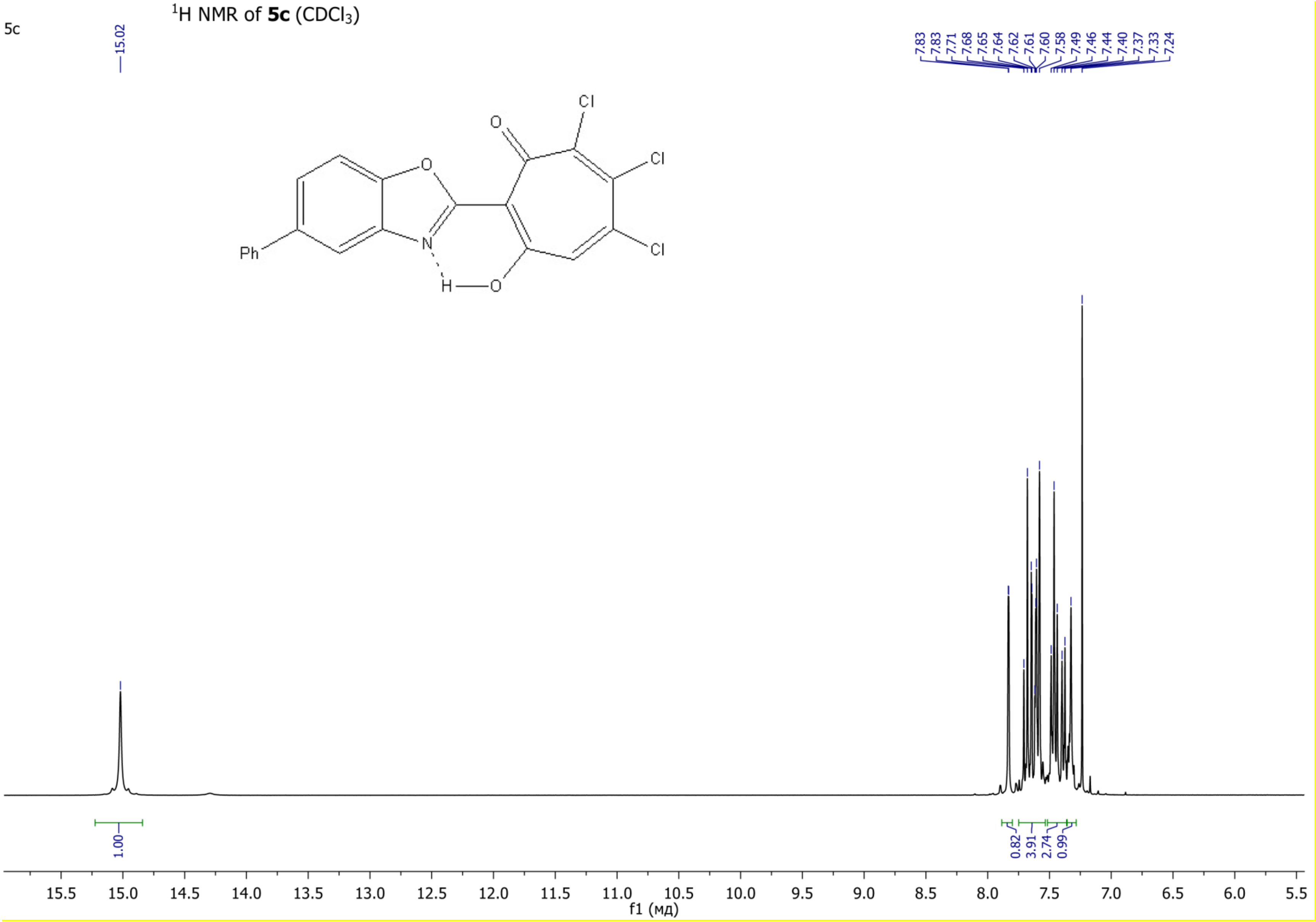

^{1}H NMR of **5c** ($CDCl_3$)
15.02
7.83
7.83
7.71
7.68
7.65
7.64
7.62
7.61
7.60
7.58
7.49
7.46
7.44
7.40
7.37
7.33
7.24
Cl
O
Cl
O
Cl
Ph
N
H
O
1.00
0.82
3.91
2.74
0.99
15.5
15.0
14.5
14.0
13.5
13.0
12.5
12.0
11.5
11.0
10.5
10.0
9.5
9.0
8.5
8.0
7.5
7.0
6.5
6.0
5.5
f1 (мд)

5c ^{13}C NMR of **5c** (DMSO)

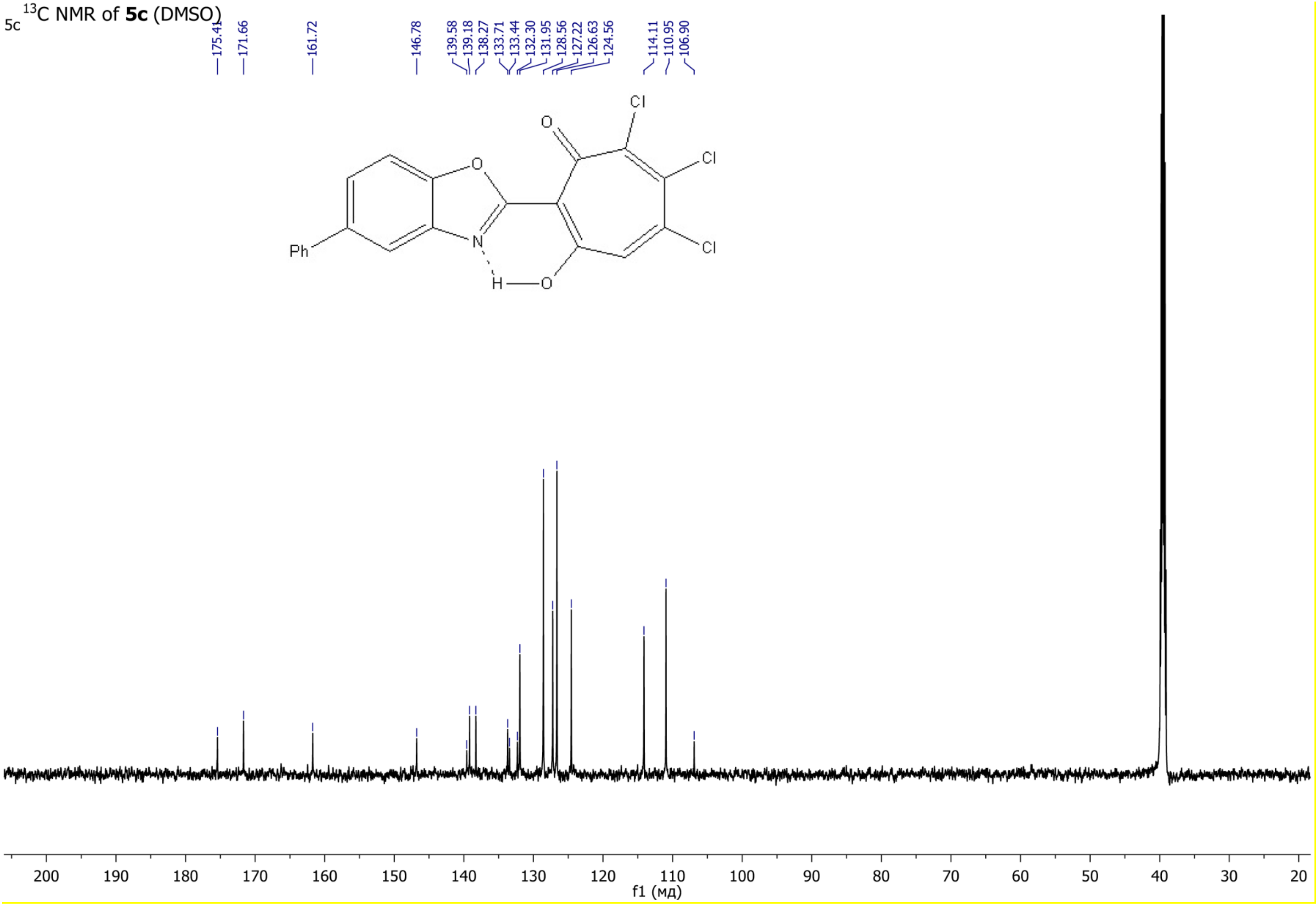

5d

^{1}H NMR of **5d** ($CDCl_3$)

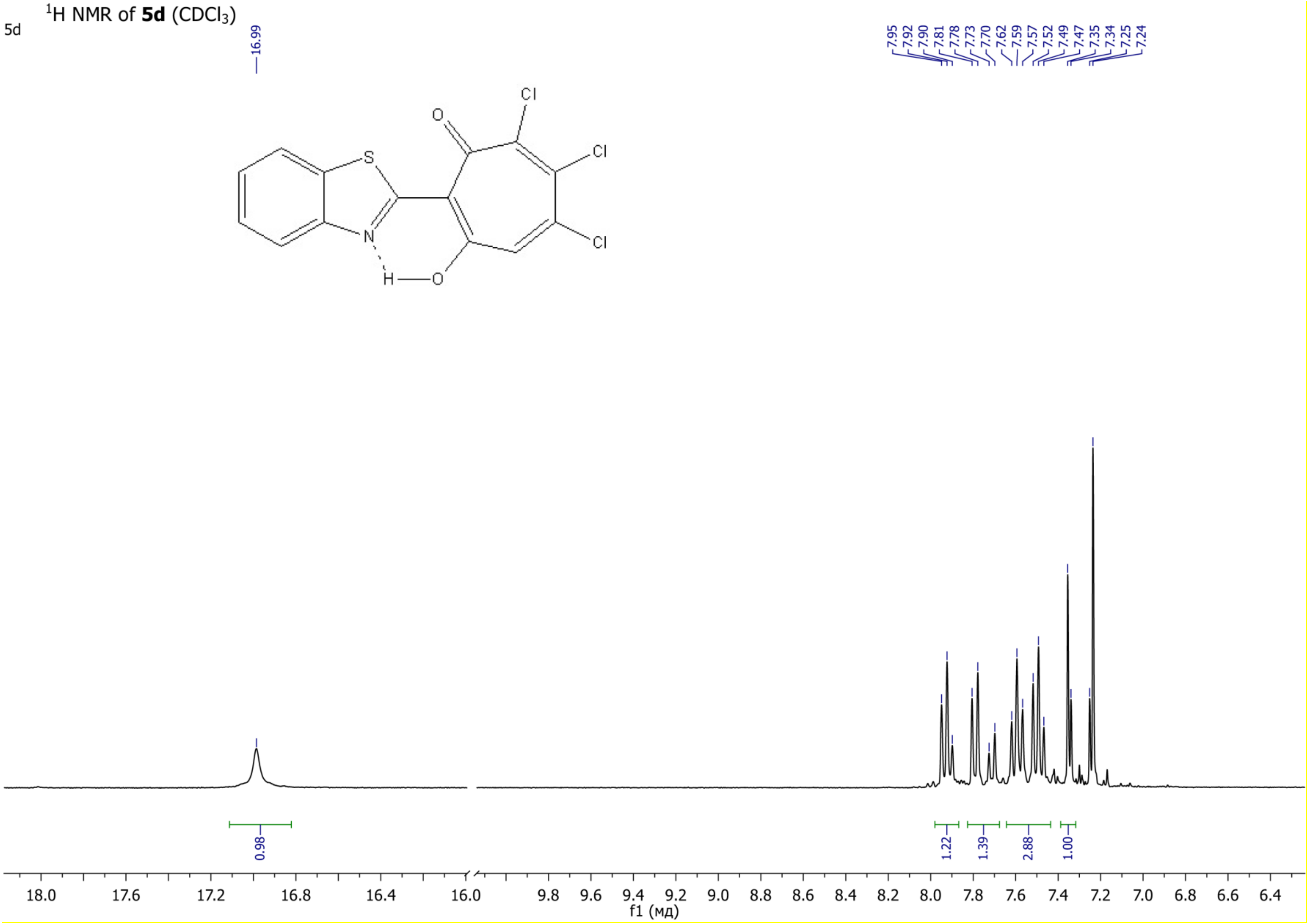

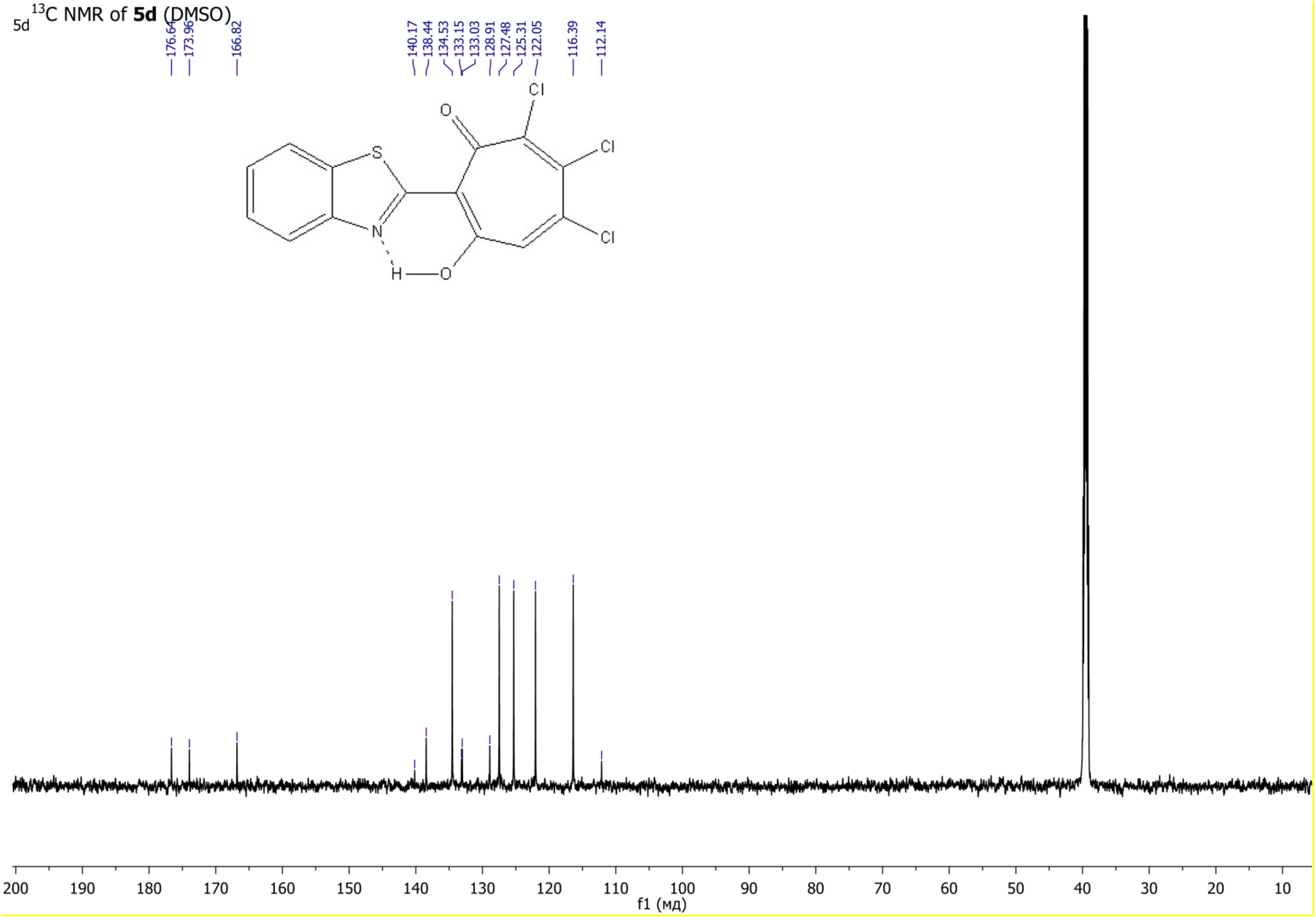

5d ^{13}C NMR of **5d** (DMSO)
176.64
173.96
166.82
140.17
138.44
134.53
133.15
133.03
128.91
127.48
125.31
122.05
116.39
112.14
O
Cl
Cl
Cl
S
N
H
O
200 190 180 170 160 150 140 130 120 110 100 90 80 70 60 50 40 30 20 10
f1 (мд)

^{1}H NMR of **5e** (DMSO_d$_6$)

5e

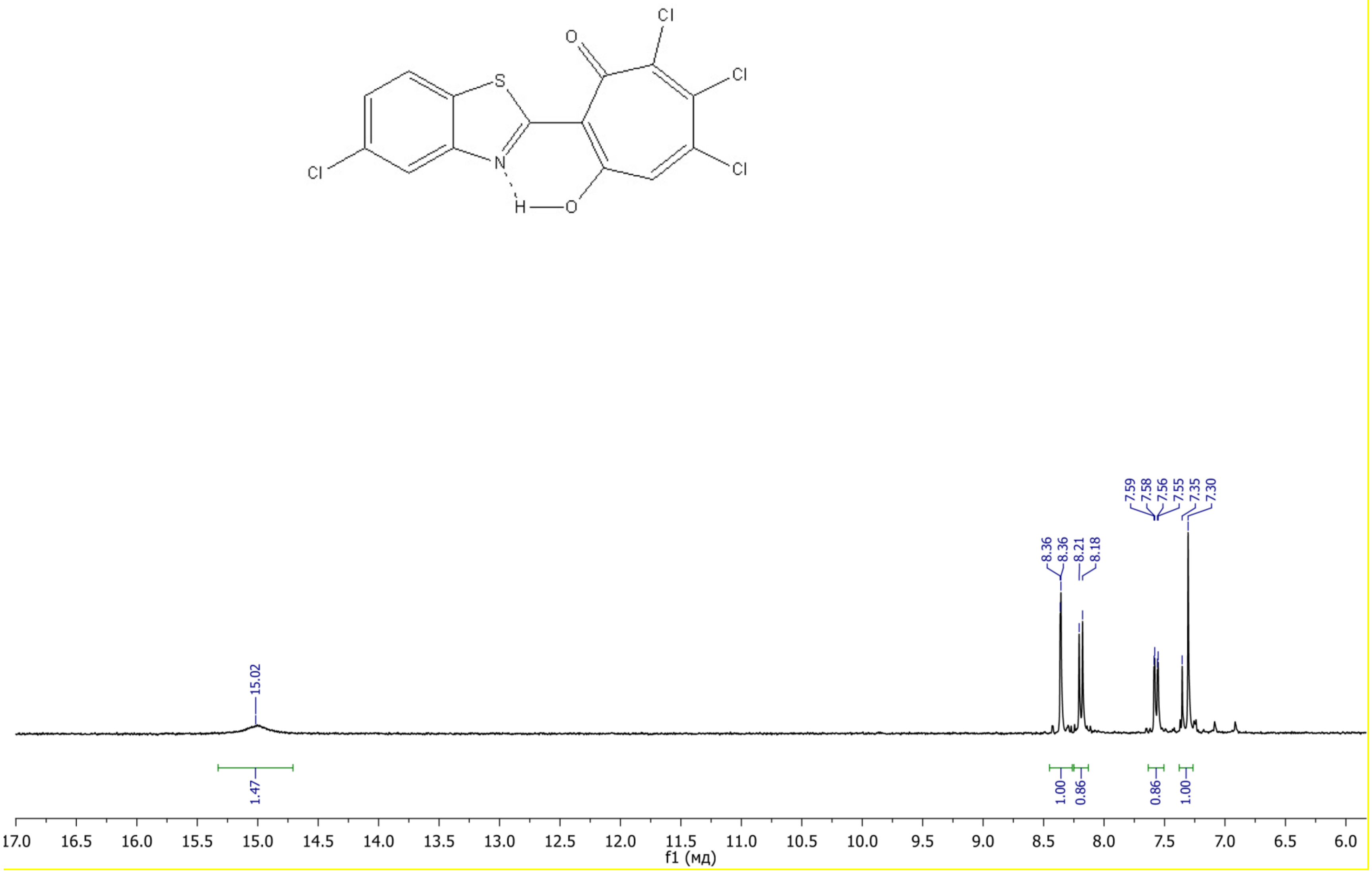

5e ^{13}C NMR of **5e** (DMSO)

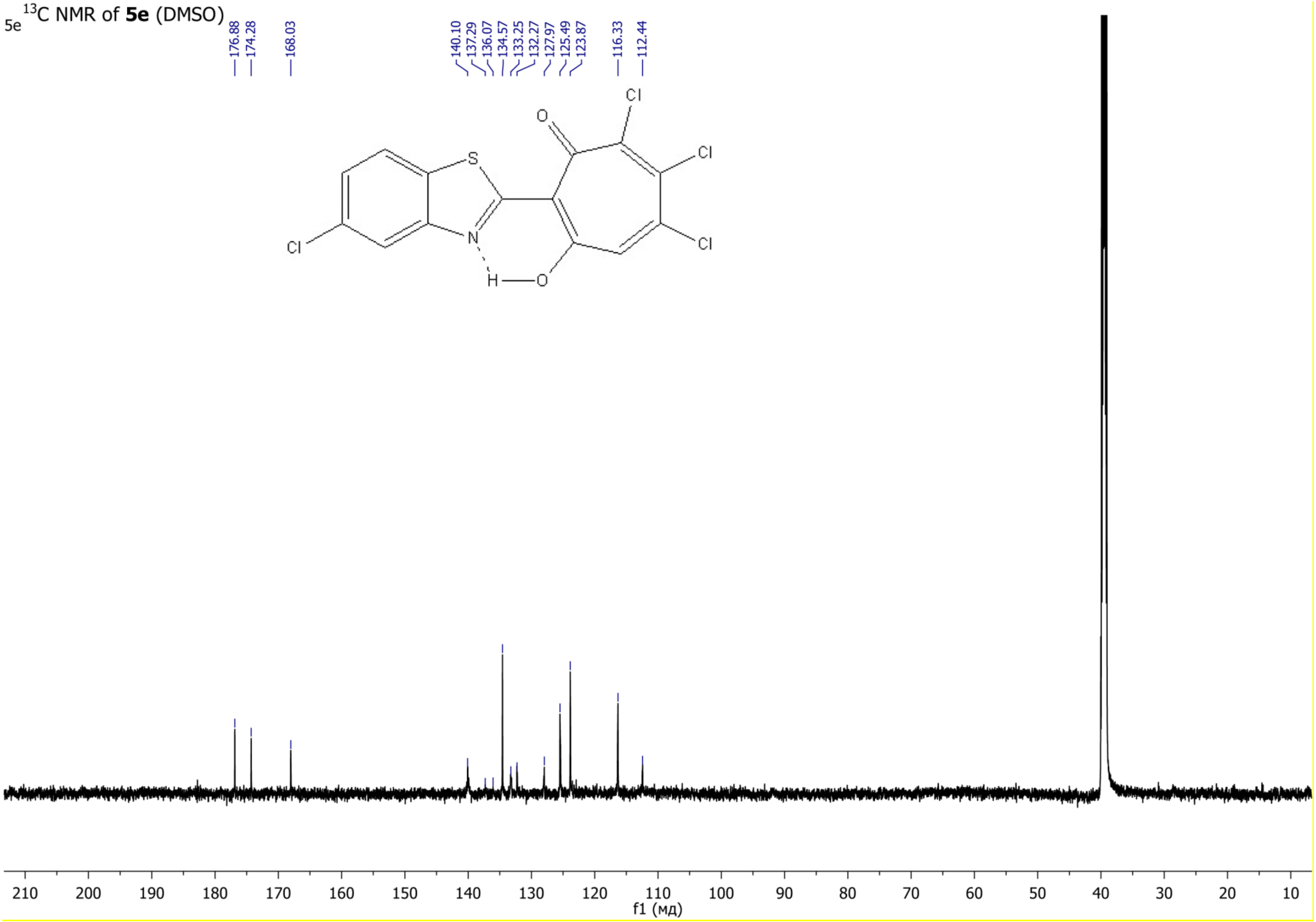

^{1}H NMR of **5f** ($CDCl_3$)

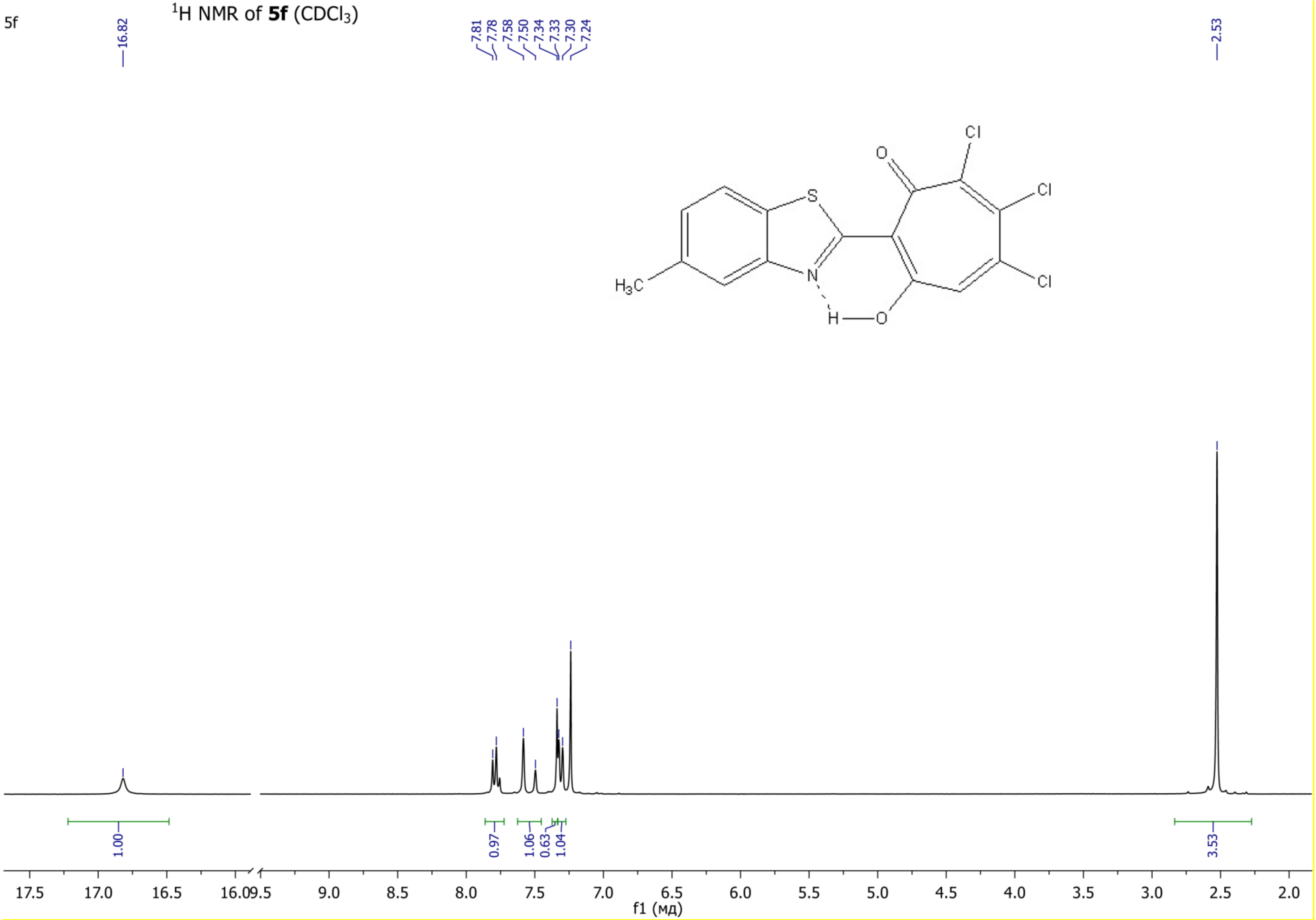

5f ^{13}C NMR of **5f** (DMSO)

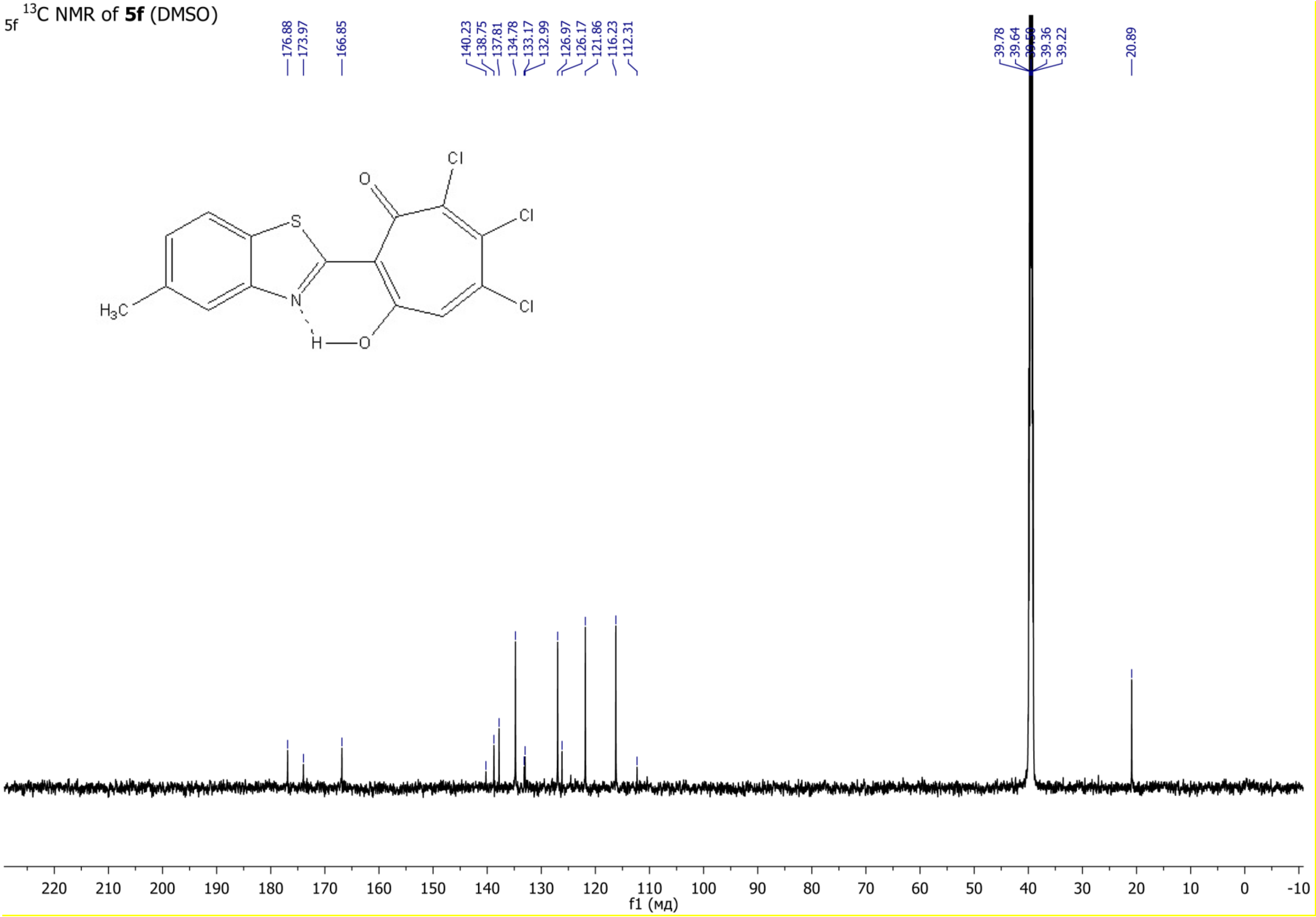

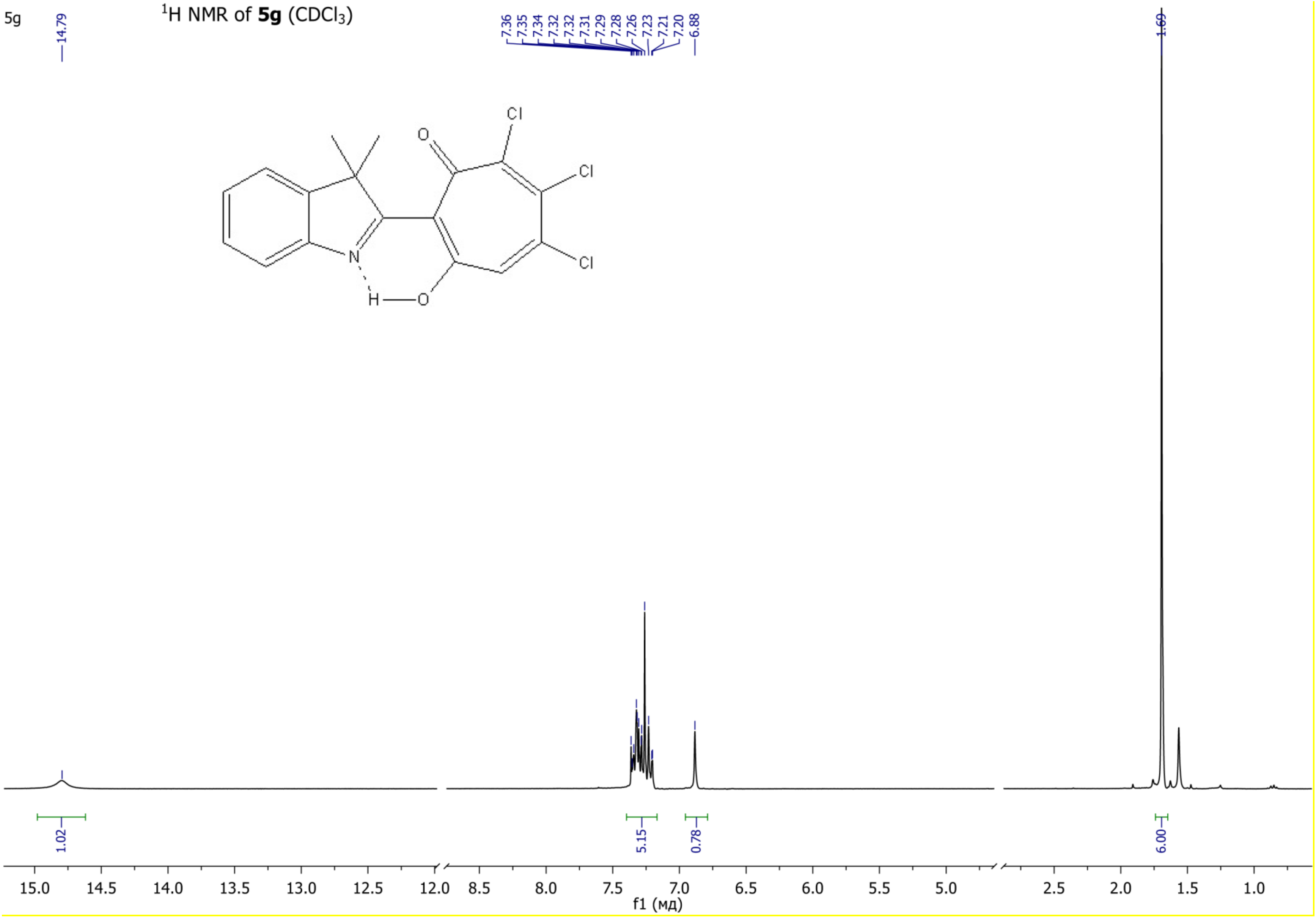
5g
¹H NMR of 5g (CDCl3)
14.79
7.36 7.35 7.34 7.32 7.32 7.31 7.29 7.28 7.26 7.23 7.21 7.20 6.88
1.69
O
Cl
Cl
Cl
N
H
O
1.02
5.15
0.78
6.00
15.0 14.5 14.0 13.5 13.0 12.5 12.0 8.5 8.0 7.5 7.0 6.5 6.0 5.5 5.0 2.5 2.0 1.5 1.0
f1 (мд)

5g ^{13}C NMR of **5g** (DMSO)

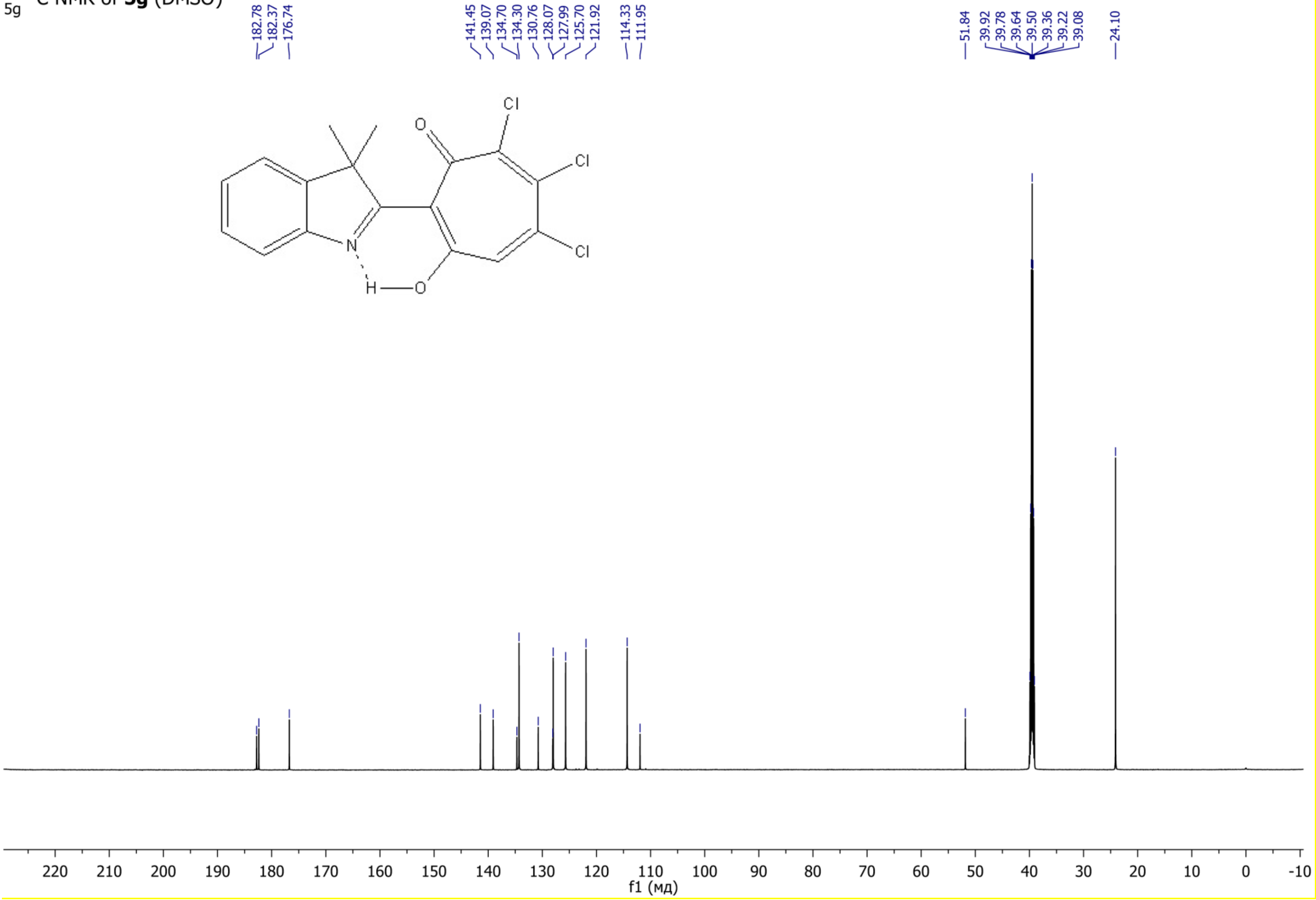

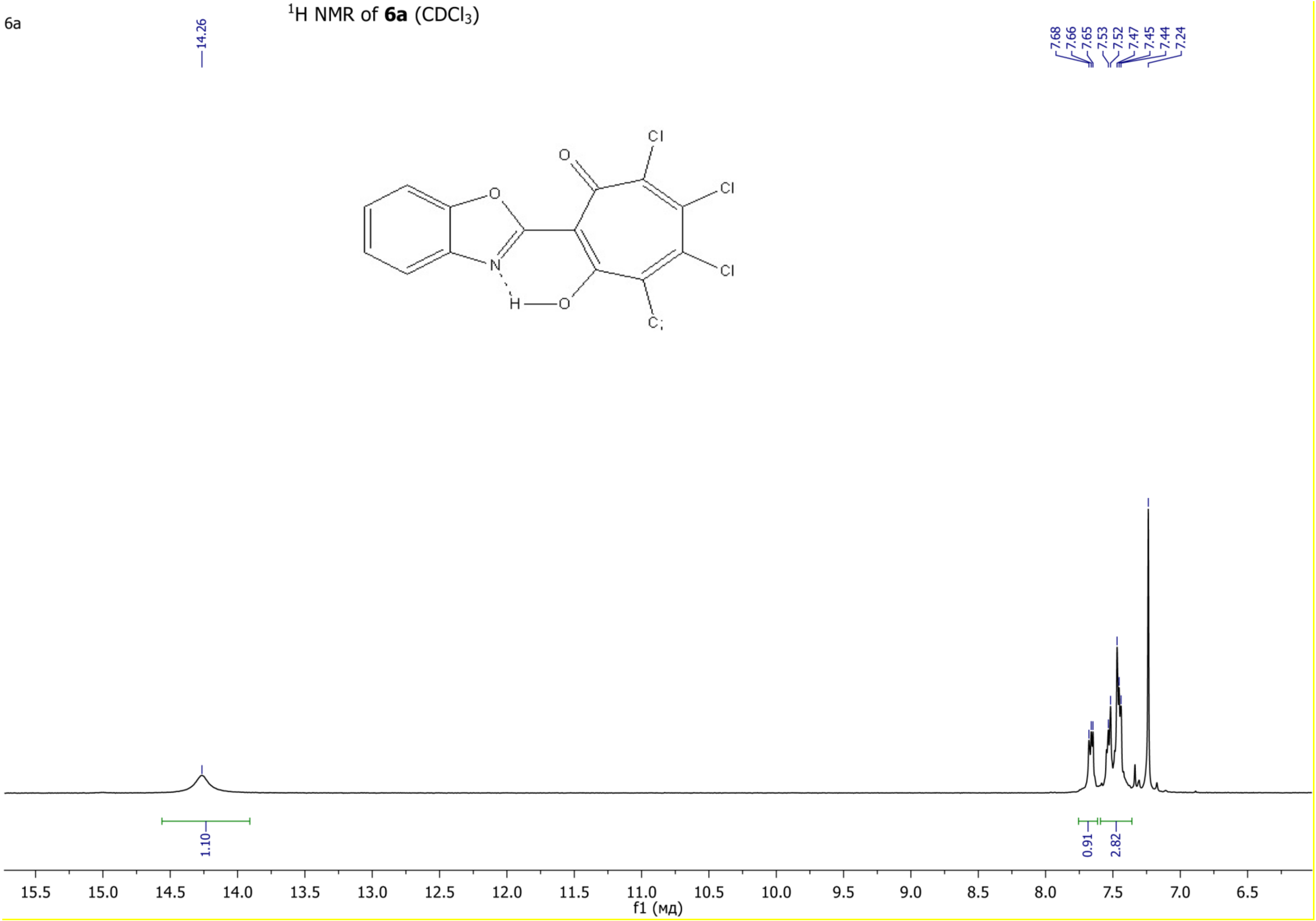
6a
¹H NMR of 6a (CDCl3)
14.26
7.68
7.66
7.65
7.53
7.52
7.47
7.45
7.44
7.24
O
Cl
Cl
Cl
N
H
1.10
0.91
2.82
15.5 15.0 14.5 14.0 13.5 13.0 12.5 12.0 11.5 11.0 10.5 10.0 9.5 9.0 8.5 8.0 7.5 7.0 6.5
f1 (мд)

6a ^{13}C NMR of **6a** (DMSO)

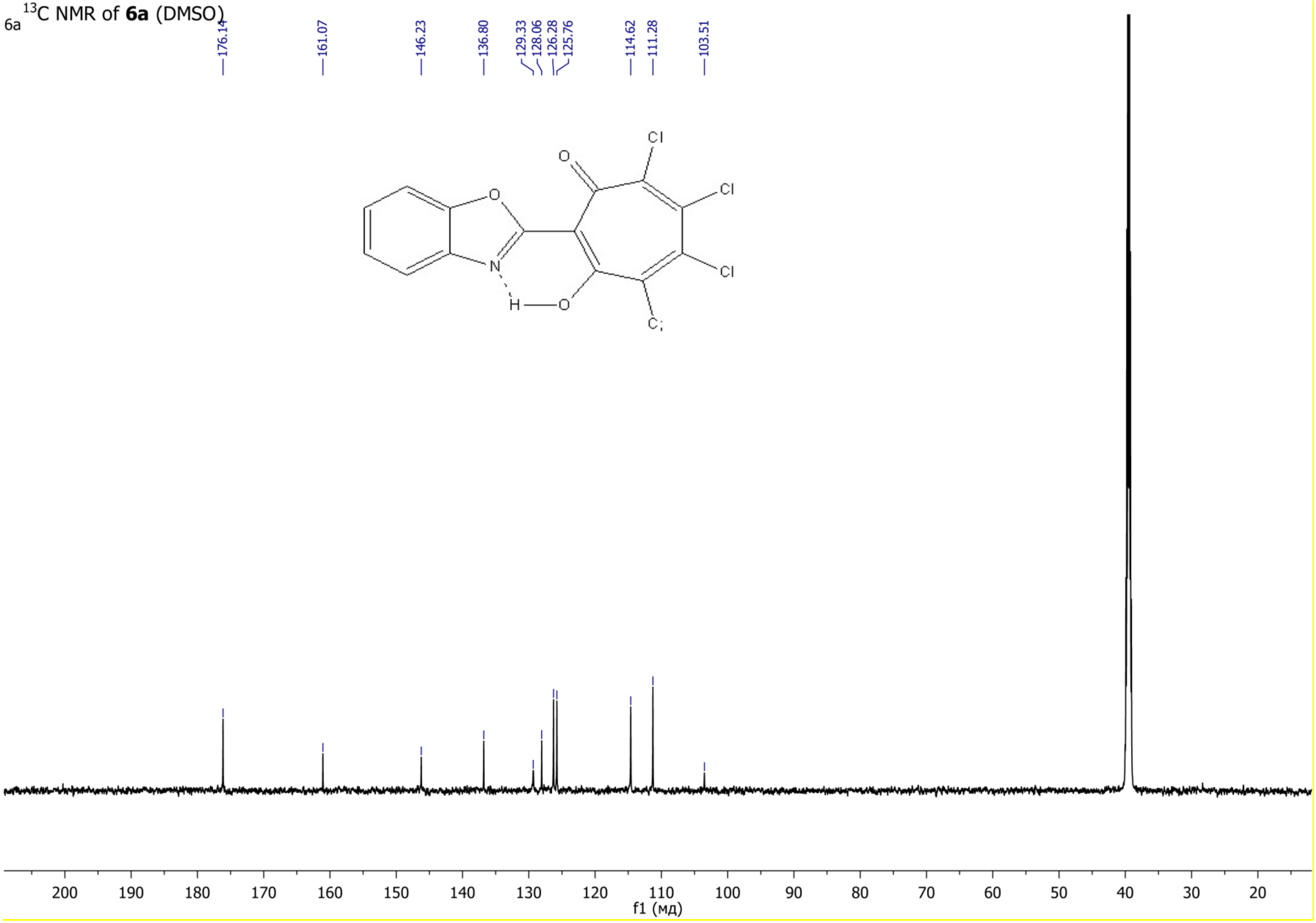

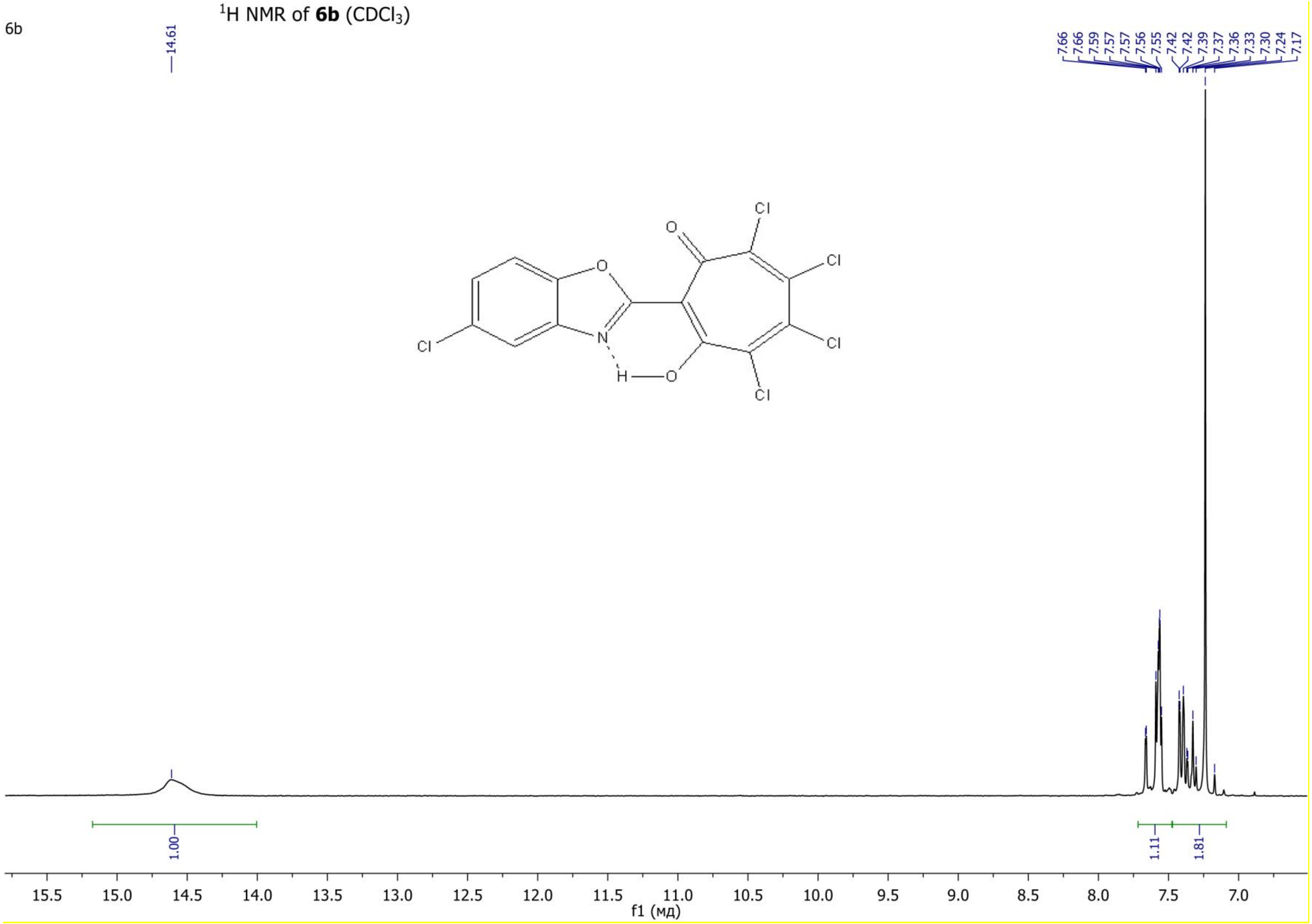

^{1}H NMR of **6b** ($CDCl_3$)

6b ^{13}C NMR of **6b** (DMSO)

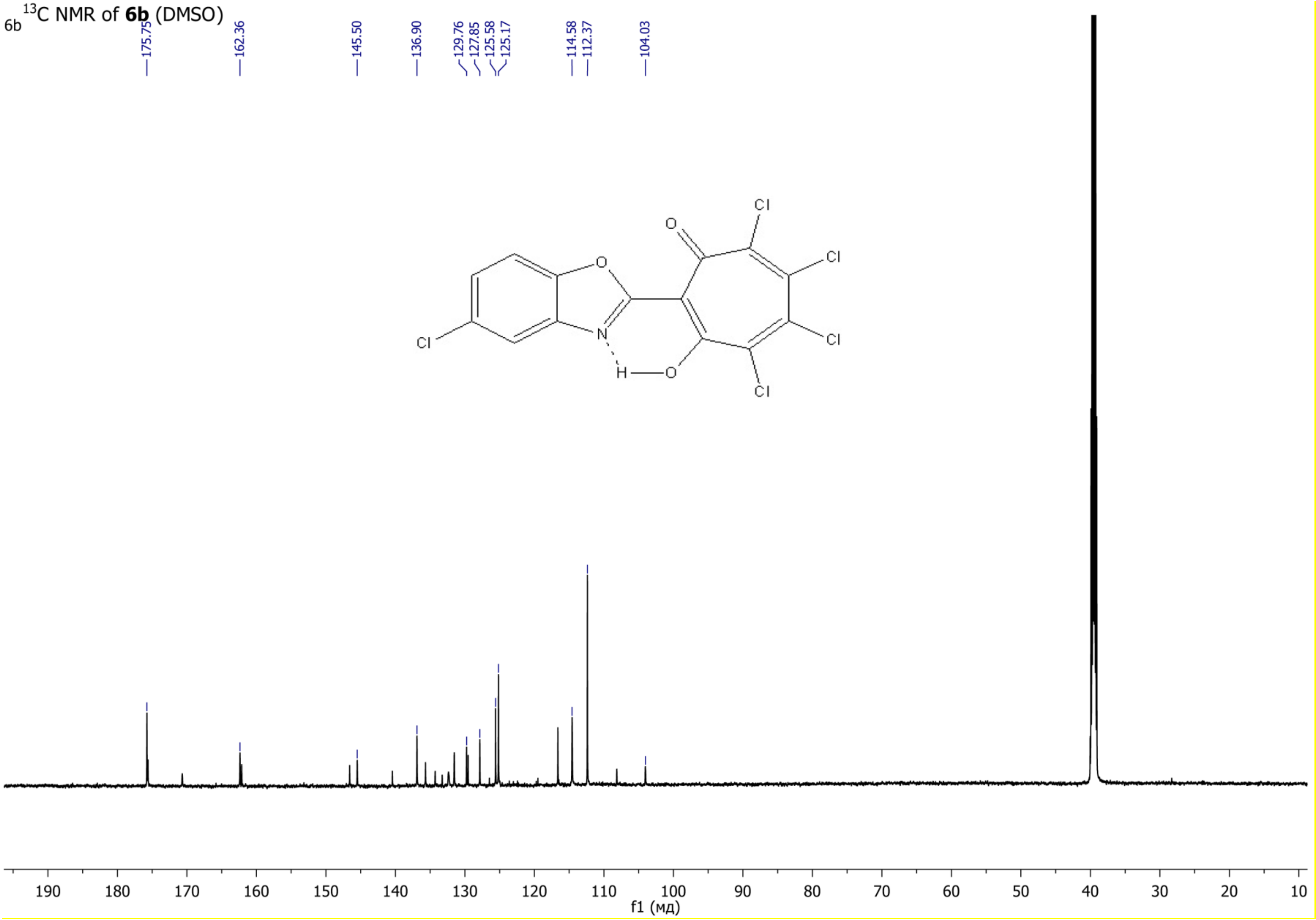

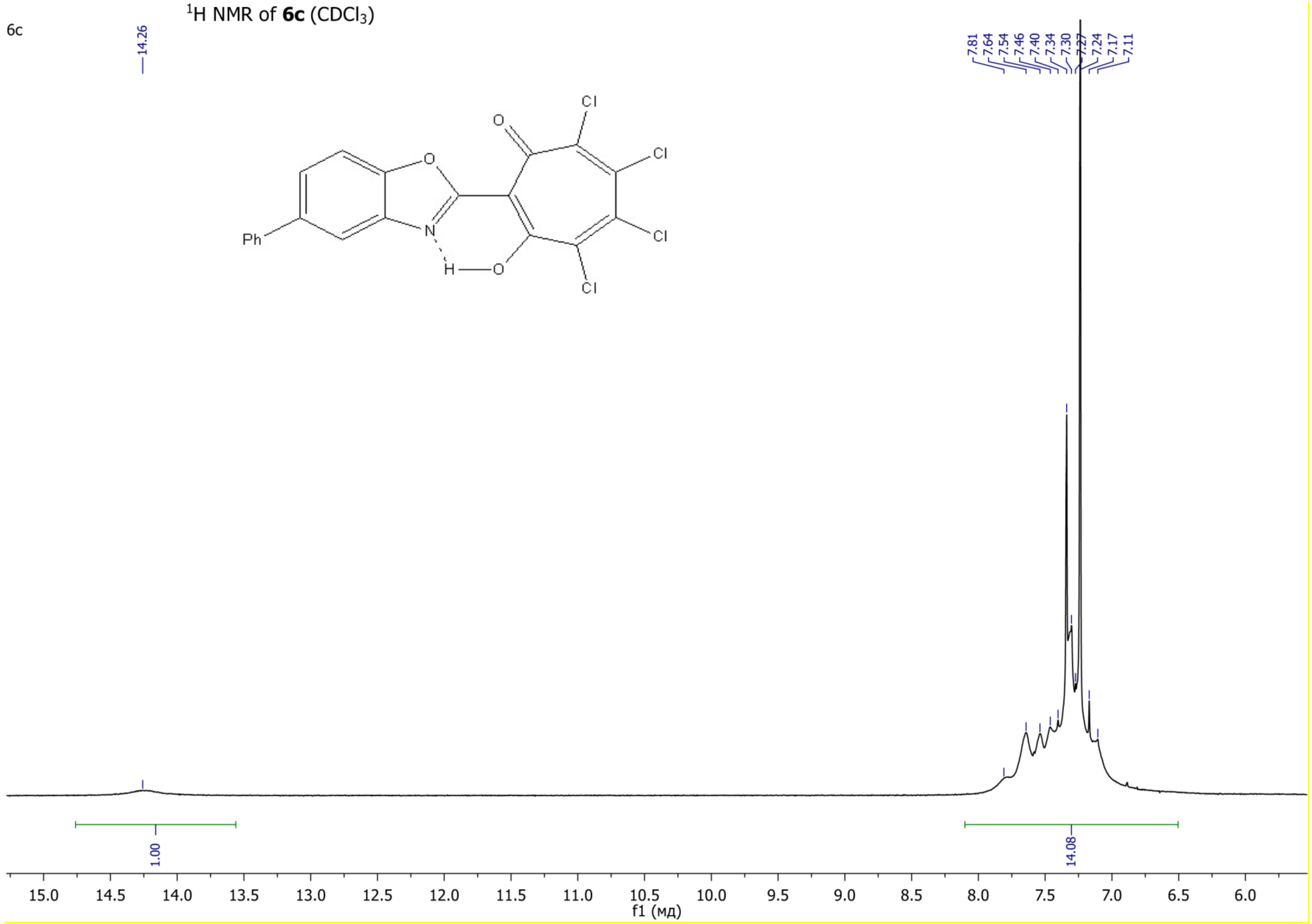

6c
^{1}H NMR of **6c** ($CDCl_3$)
14.26
7.81
7.64
7.54
7.46
7.40
7.34
7.30
7.27
7.24
7.17
7.11
O
Cl
O
Cl
Cl
Ph
N
H
O
Cl
1.00
14.08
15.0
14.5
14.0
13.5
13.0
12.5
12.0
11.5
11.0
10.5
10.0
9.5
9.0
8.5
8.0
7.5
7.0
6.5
6.0
f1 (мд)

6c ^{13}C NMR of **6c** (DMSO)

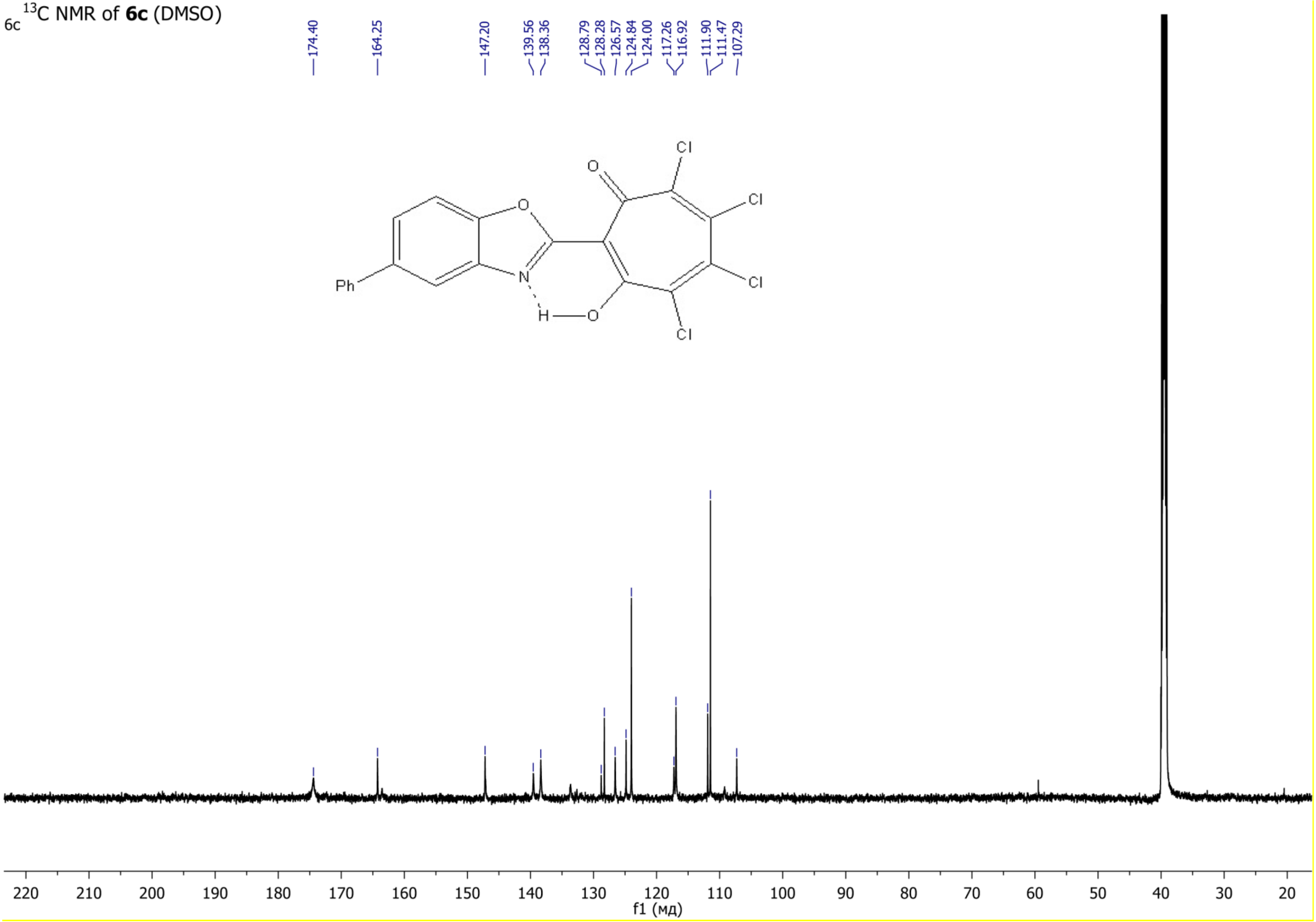

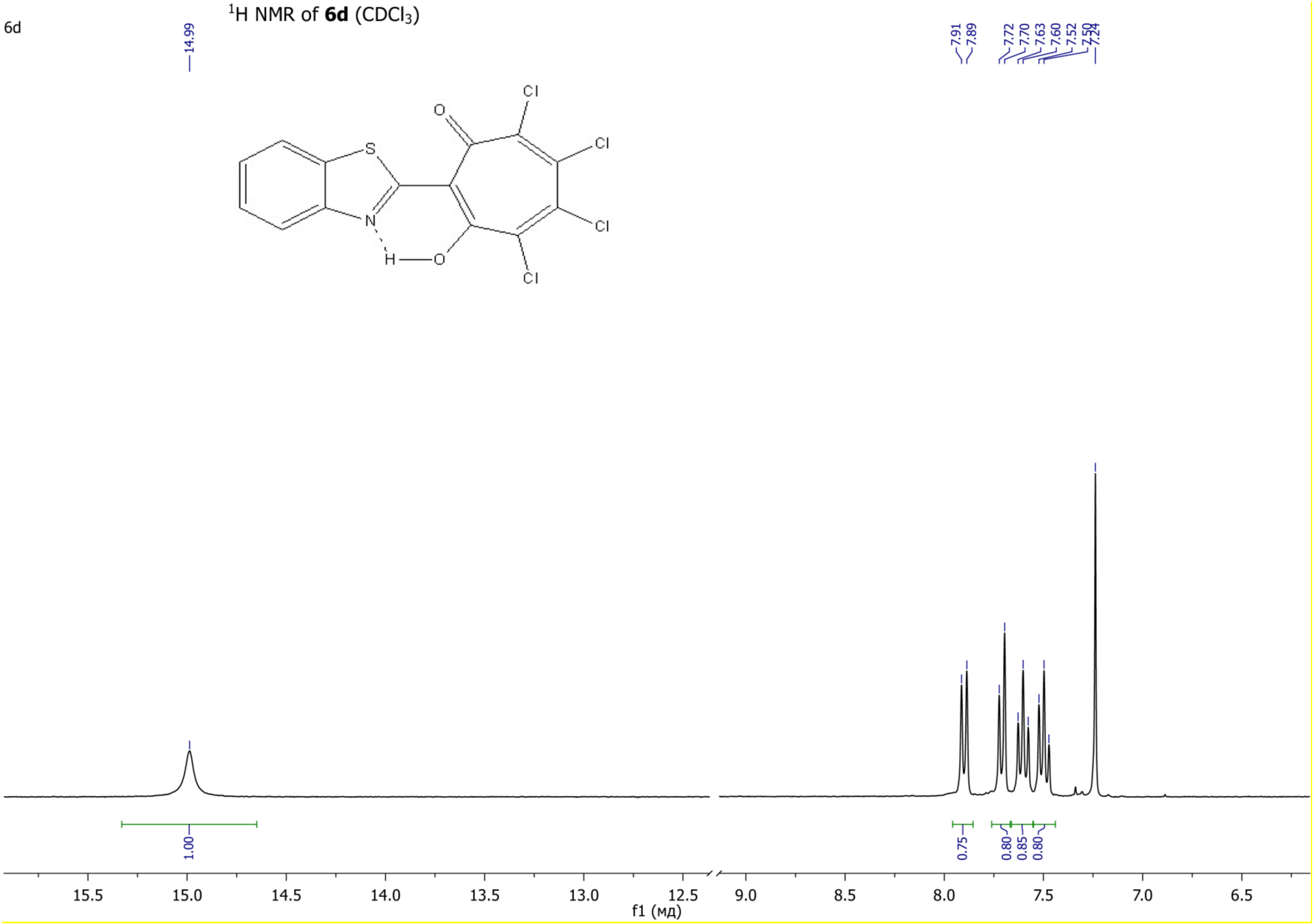

6d
^{1}H NMR of **6d** (CDCl$_3$)
14.99
7.91
7.89
7.72
7.70
7.63
7.60
7.52
7.50
7.24
Cl
O
S
Cl
N
Cl
H
O
Cl
1.00
0.75
0.80
0.85
0.80
15.5
15.0
14.5
14.0
13.5
13.0
12.5
9.0
8.5
8.0
7.5
7.0
6.5
f1 (мд)

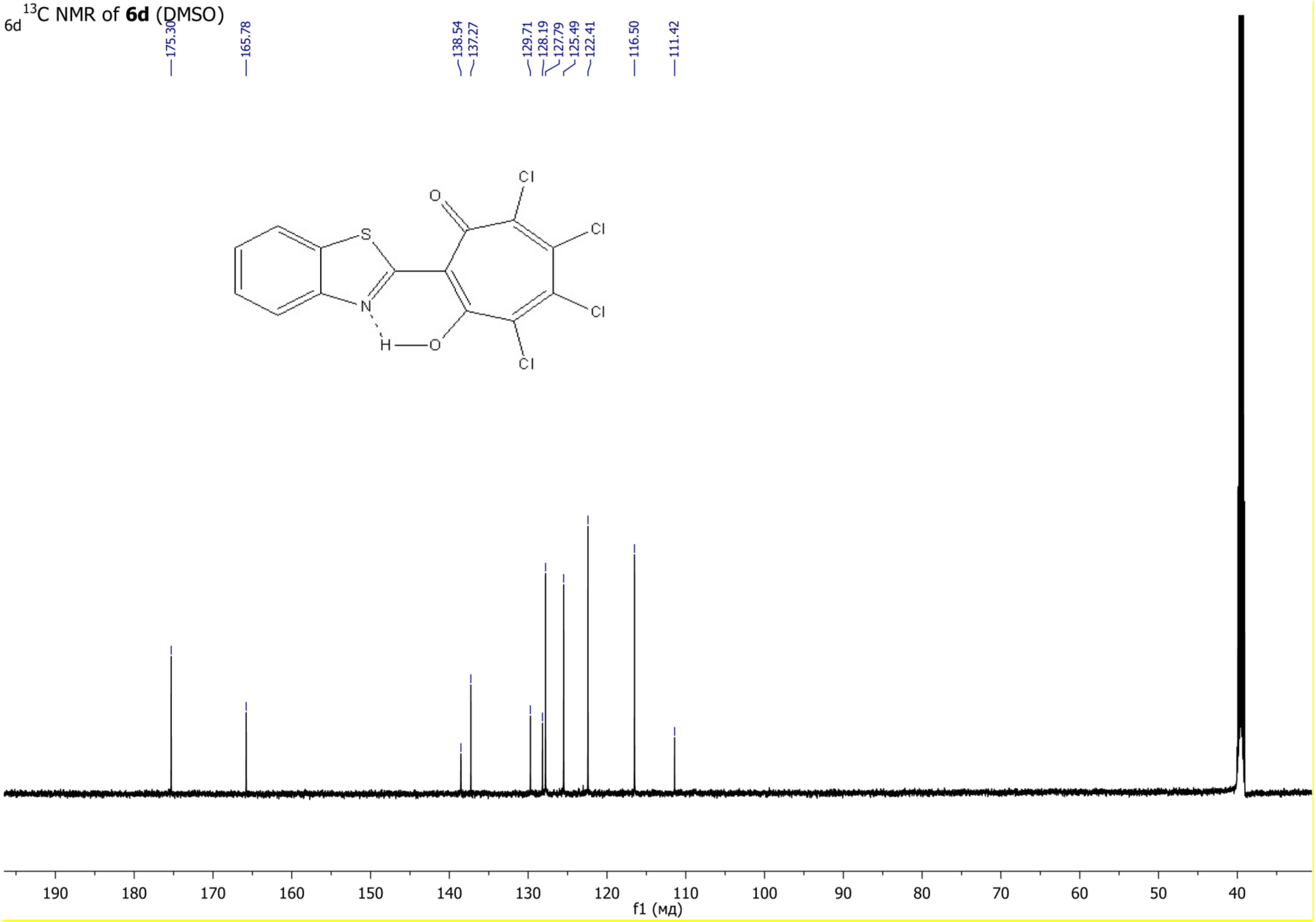
6d ^{13}C NMR of **6d** (DMSO)
175.30
165.78
138.54
137.27
129.71
128.19
127.79
125.49
122.41
116.50
111.42
O
Cl
S
N
H
190 180 170 160 150 140 130 120 110 100 90 80 70 60 50 40
f1 (мд)

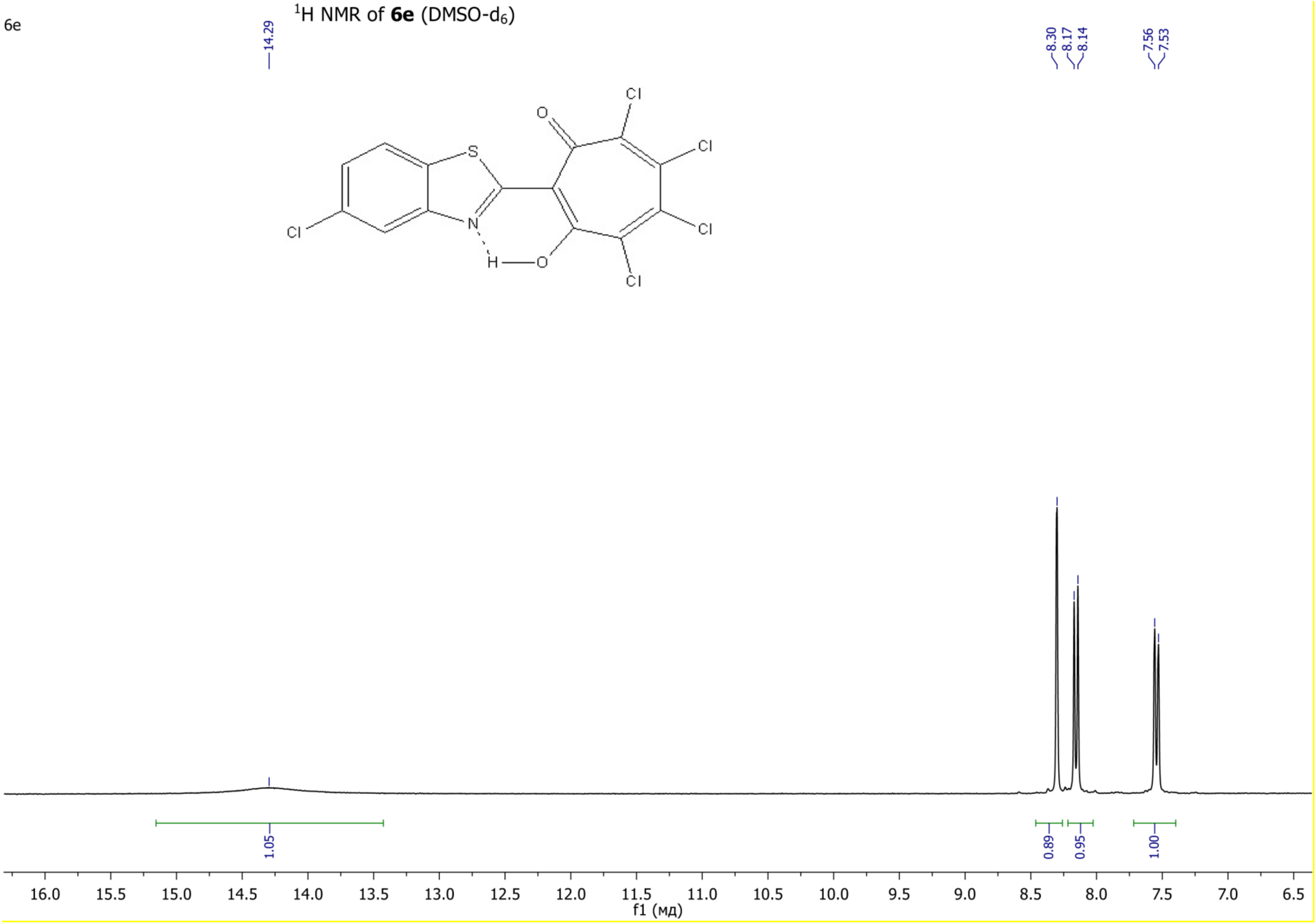
6e
^{1}H NMR of **6e** (DMSO-d_6)
14.29
8.30
8.17
8.14
7.56
7.53
Cl
O
S
N
H
O
Cl
Cl
Cl
Cl
1.05
0.89
0.95
1.00
16.0
15.5
15.0
14.5
14.0
13.5
13.0
12.5
12.0
11.5
11.0
10.5
10.0
9.5
9.0
8.5
8.0
7.5
7.0
6.5
f1 (мд)

6e ^{13}C NMR of **6e** (DMSO)

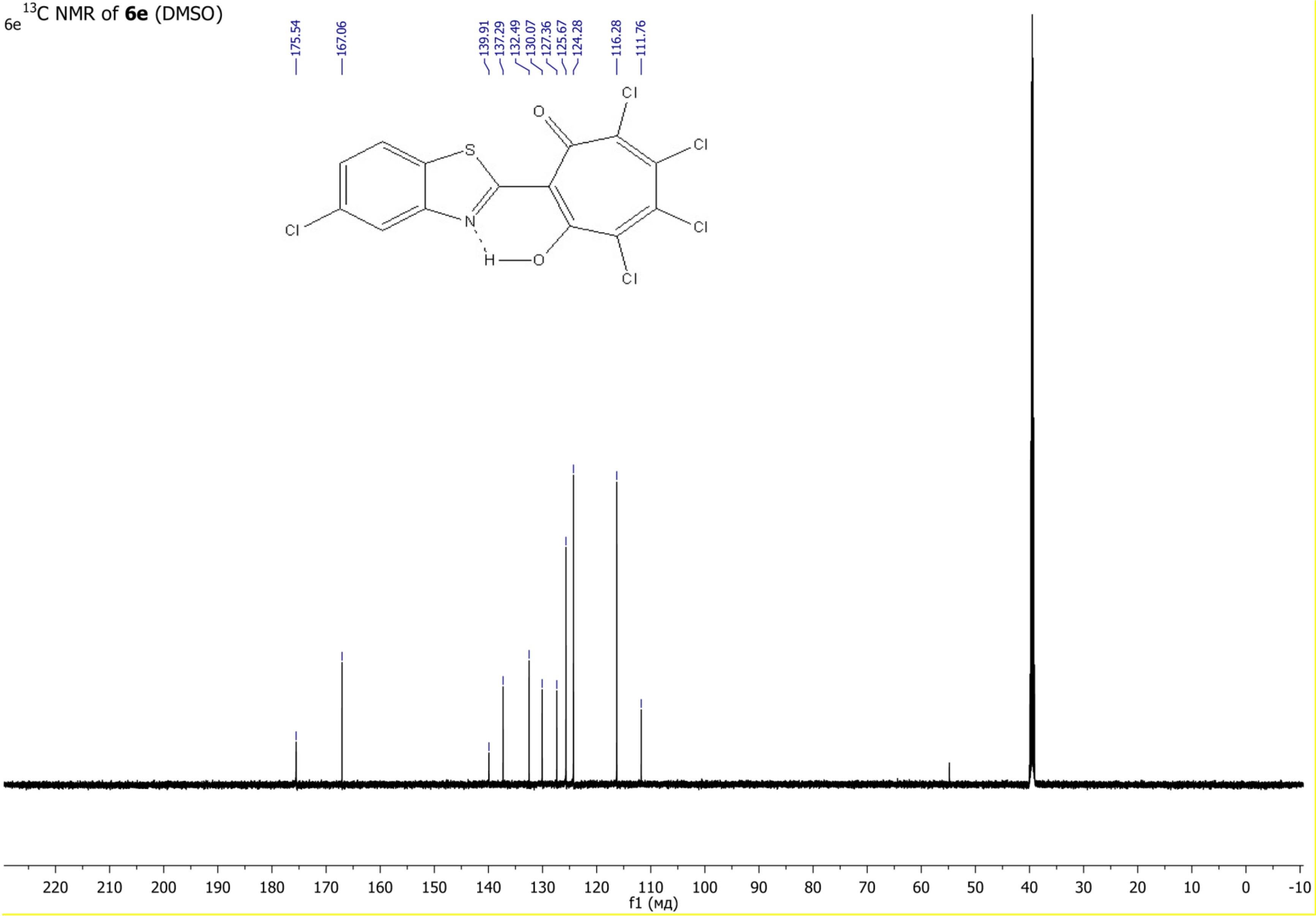

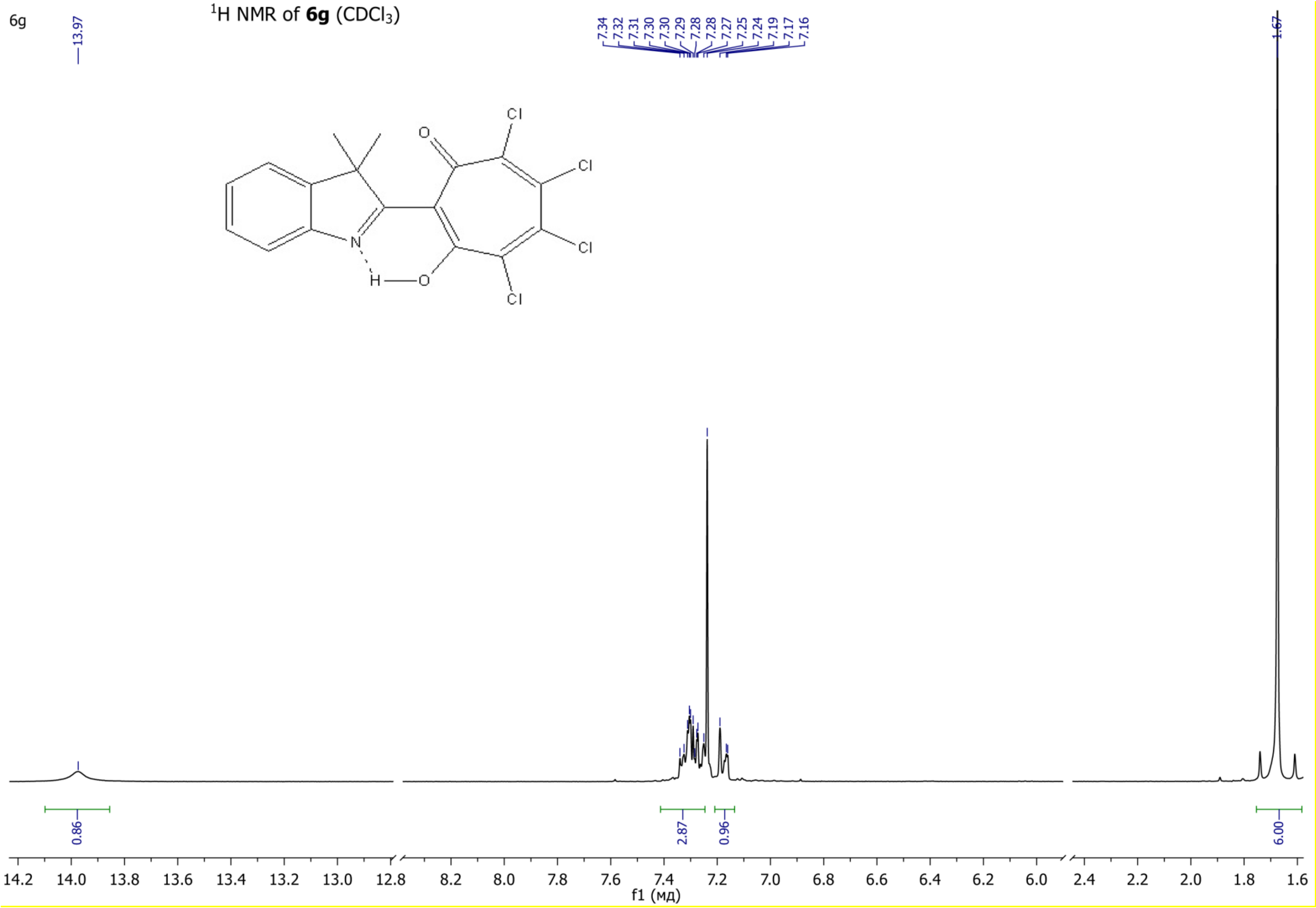

6g
^{1}H NMR of **6g** ($CDCl_3$)
13.97
7.34 7.32 7.31 7.30 7.30 7.29 7.28 7.28 7.27 7.25 7.24 7.19 7.17 7.16
1.67
Cl
Cl
Cl
Cl
O
N
H
O
0.86
2.87
0.96
6.00
14.2 14.0 13.8 13.6 13.4 13.2 13.0 12.8 8.2 8.0 7.8 7.6 7.4 7.2 7.0 6.8 6.6 6.4 6.2 6.0 2.4 2.2 2.0 1.8 1.6
f1 (мд)

6g ^{13}C NMR of **6g** (DMSO)

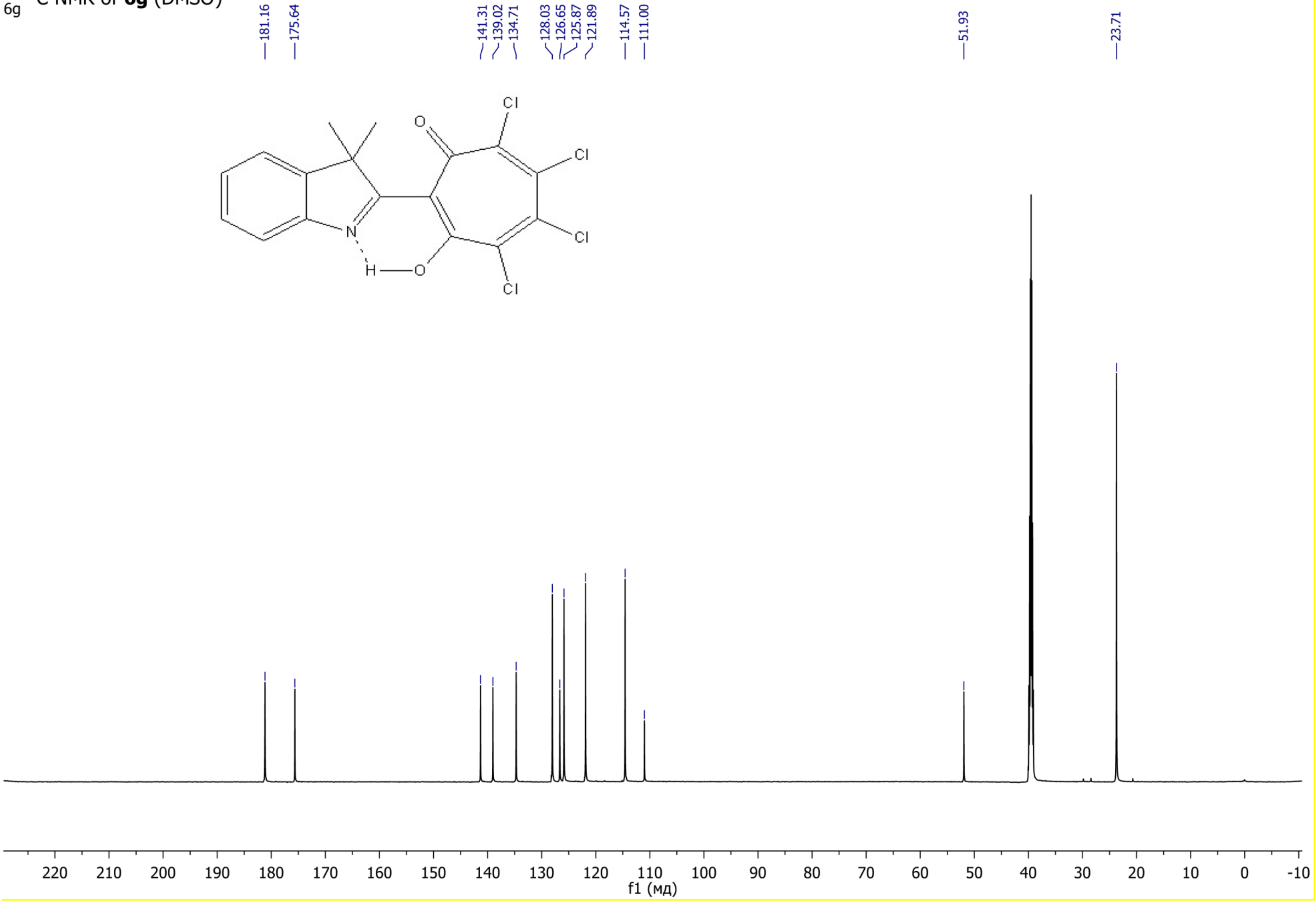

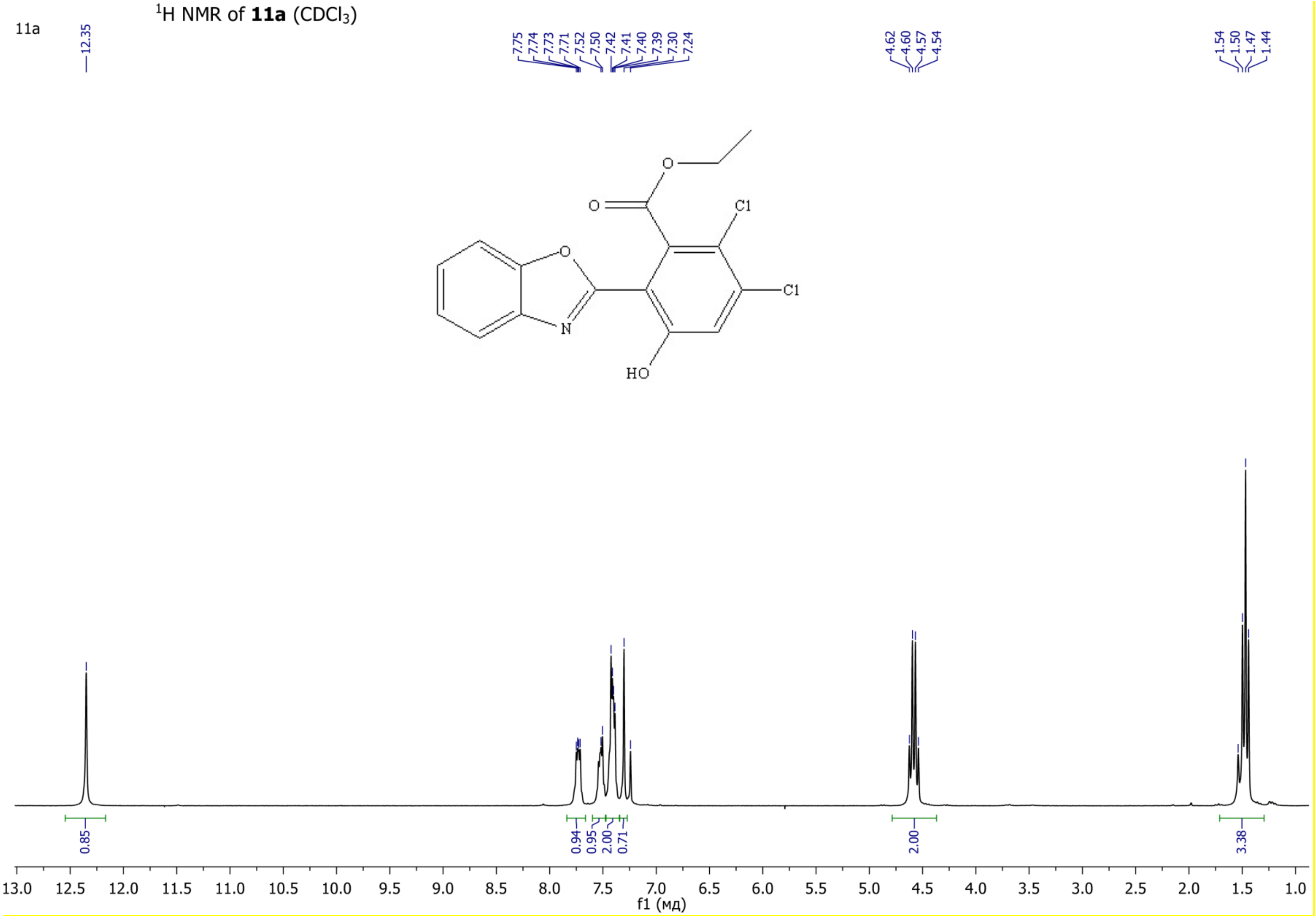

11a
^{1}H NMR of **11a** ($CDCl_3$)
12.35
7.75 7.74 7.73 7.71 7.52 7.50 7.42 7.41 7.40 7.39 7.30 7.24
4.62 4.60 4.57 4.54
1.54 1.50 1.47 1.44
O
O
Cl
Cl
O
N
HO
0.85
0.94 0.95 2.00 0.71
2.00
3.38
13.0 12.5 12.0 11.5 11.0 10.5 10.0 9.5 9.0 8.5 8.0 7.5 7.0 6.5 6.0 5.5 5.0 4.5 4.0 3.5 3.0 2.5 2.0 1.5 1.0
f1 (мд)

^{13}C NMR of **11a** ($CDCl_3$)

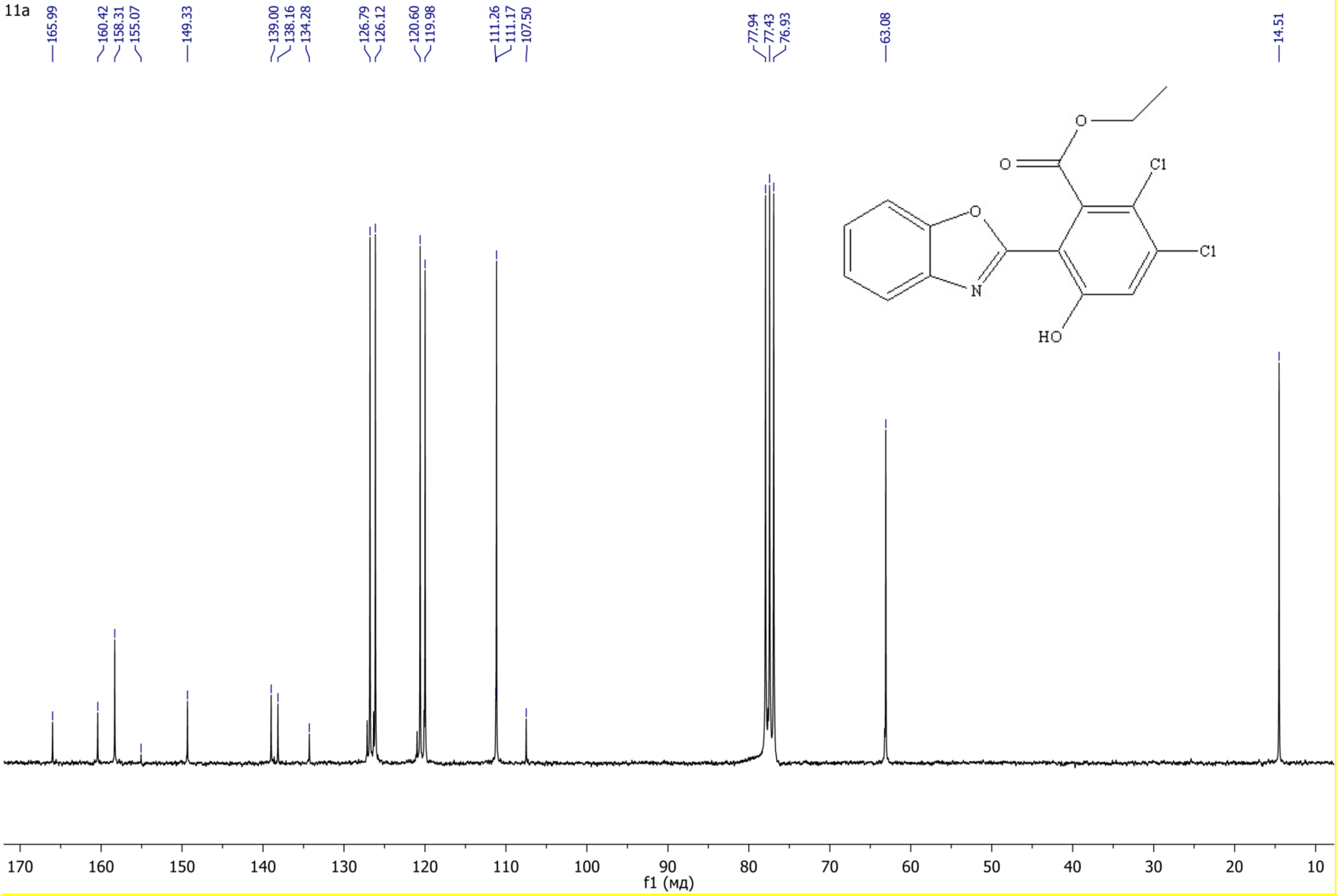

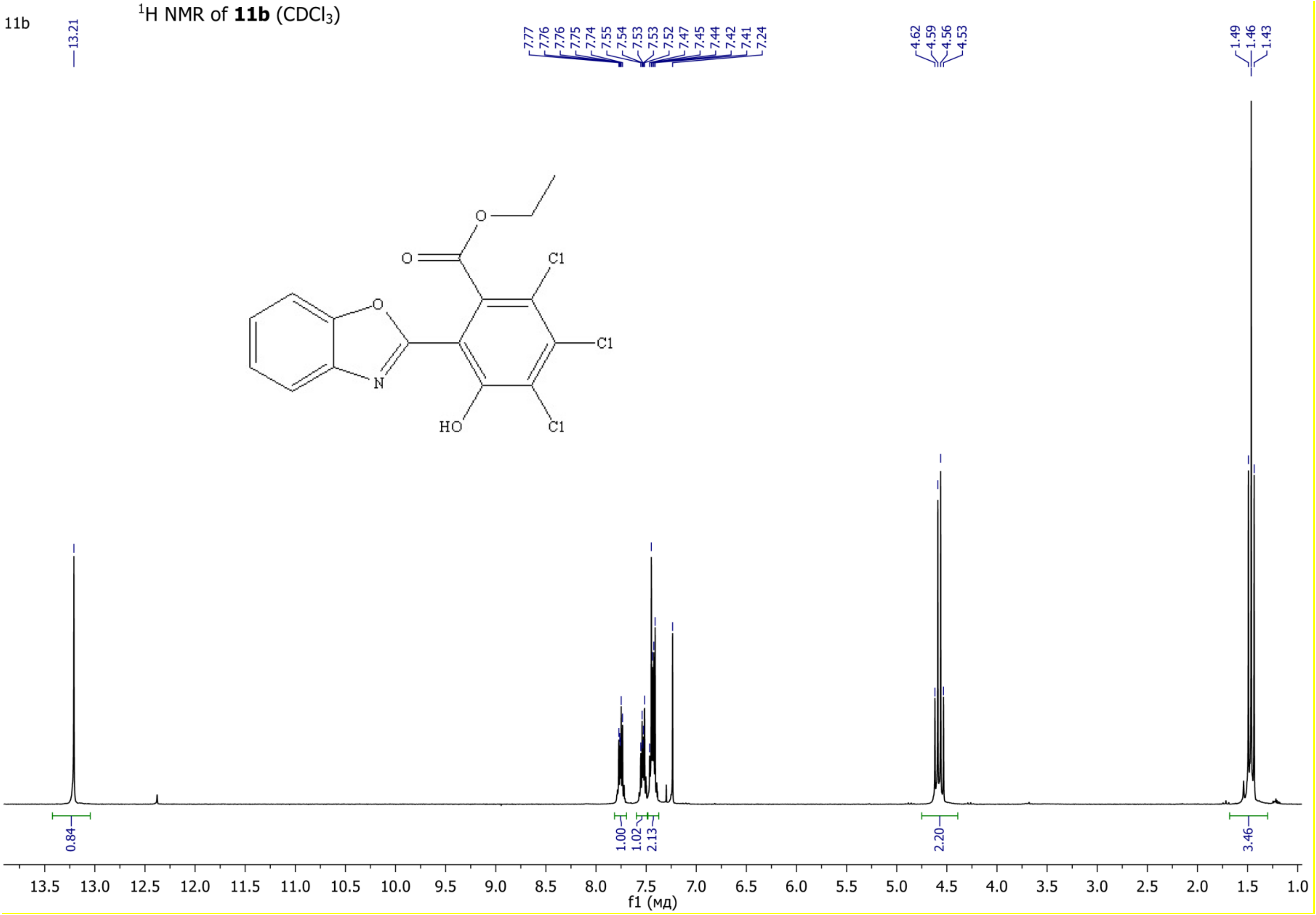
11b
^{1}H NMR of **11b** ($CDCl_3$)
13.21
7.77 7.76 7.76 7.75 7.74 7.55 7.54 7.53 7.53 7.52 7.47 7.45 7.44 7.42 7.41 7.24
4.62 4.59 4.56 4.53
1.49 1.46 1.43
O
O
Cl
O
Cl
N
HO
Cl
0.84
1.00
1.02
2.13
2.20
3.46
13.5 13.0 12.5 12.0 11.5 11.0 10.5 10.0 9.5 9.0 8.5 8.0 7.5 7.0 6.5 6.0 5.5 5.0 4.5 4.0 3.5 3.0 2.5 2.0 1.5 1.0
f1 (мд)

^{13}C NMR of **11b** ($CDCl_3$)

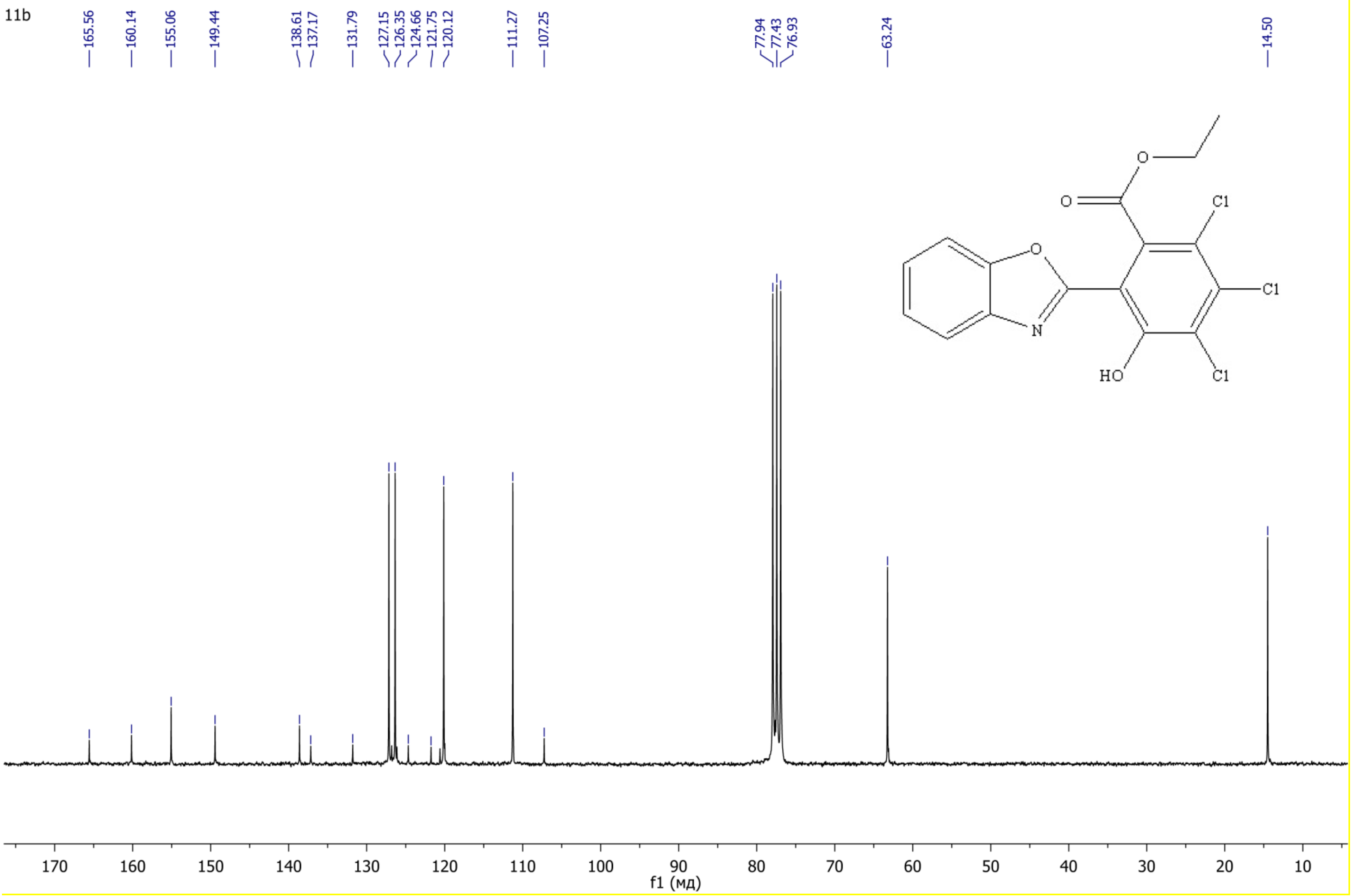

^{1}H NMR of **11c** ($CDCl_3$)

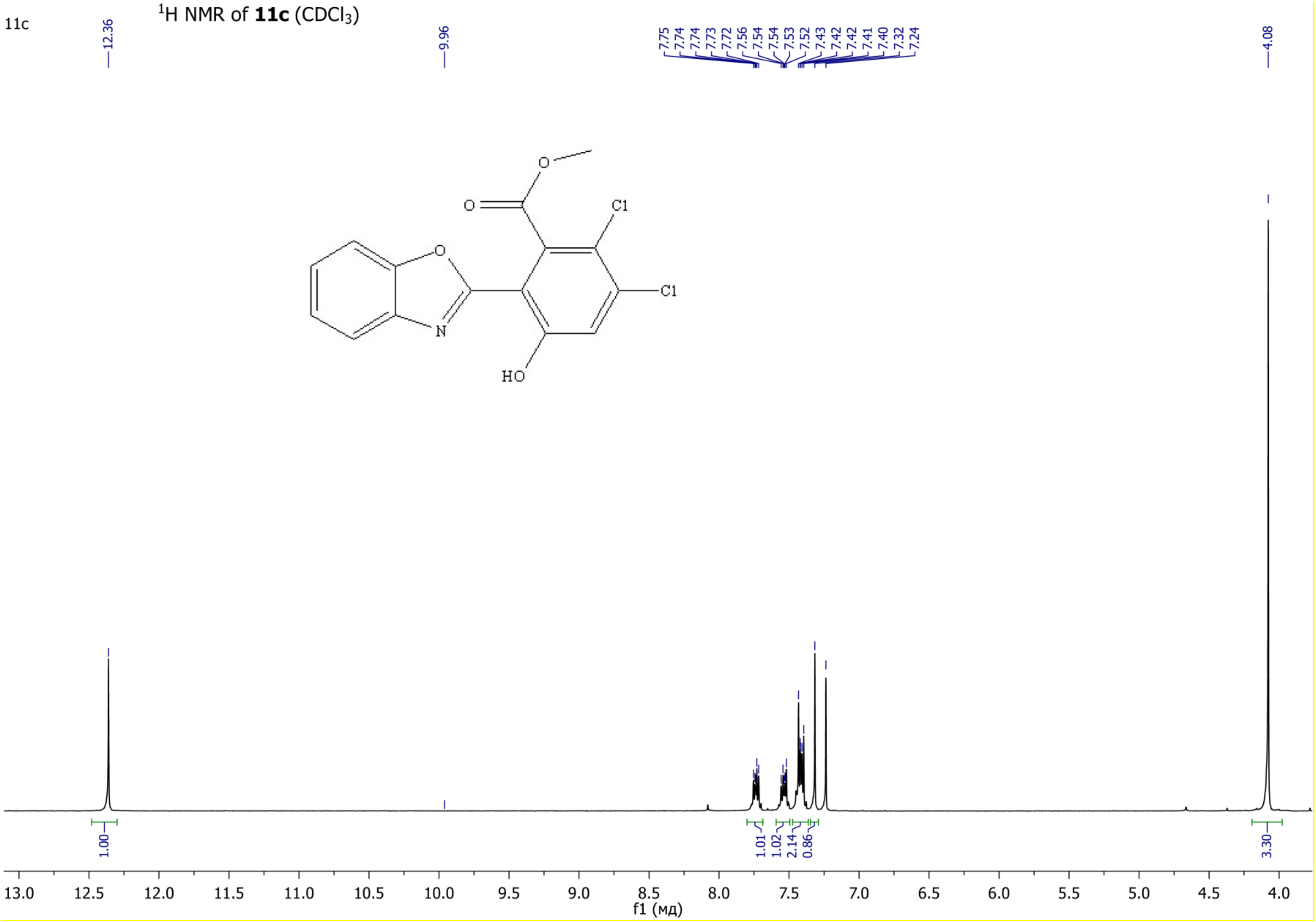

^{13}C NMR of **11c** ($CDCl_3$)

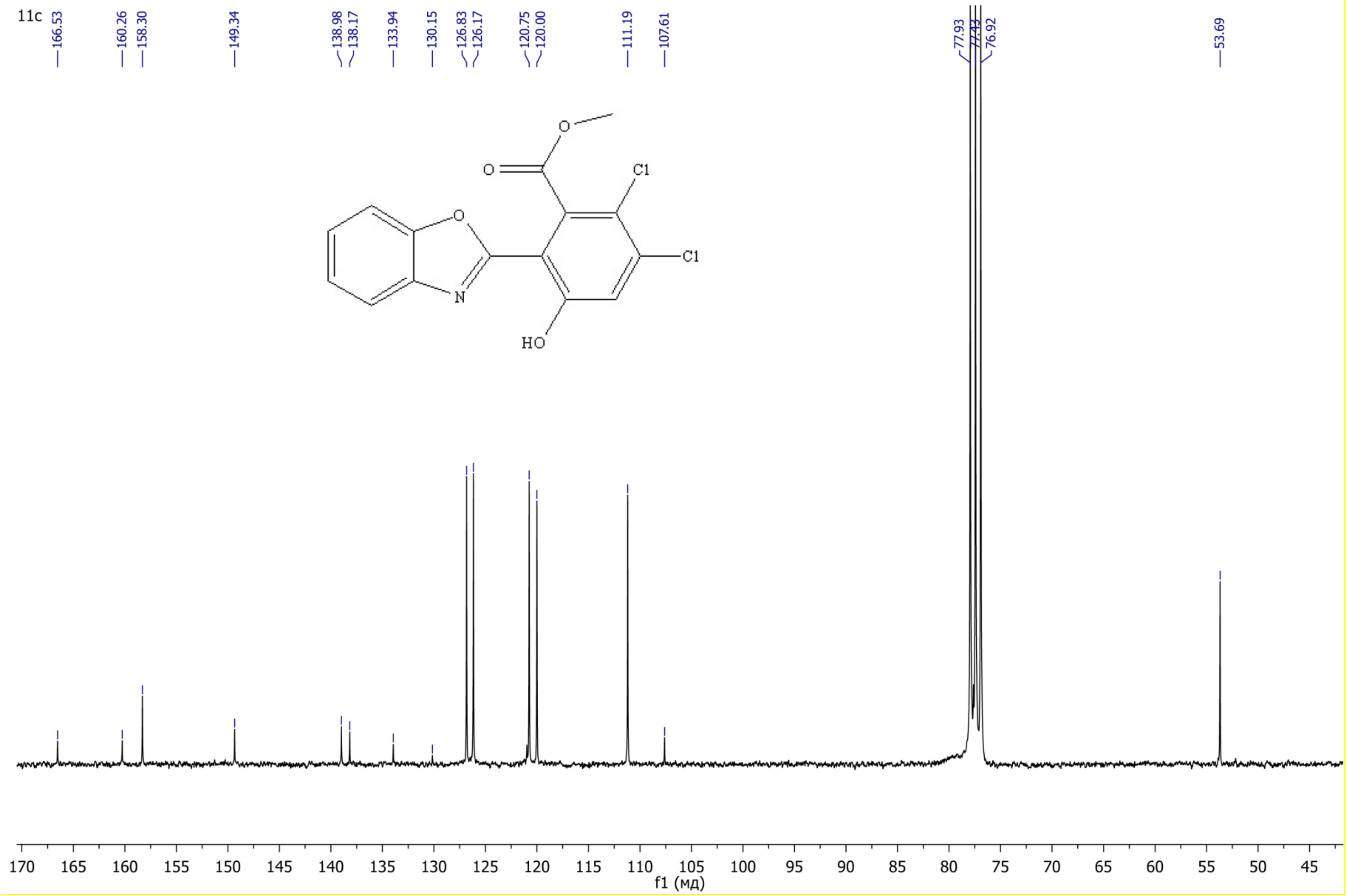

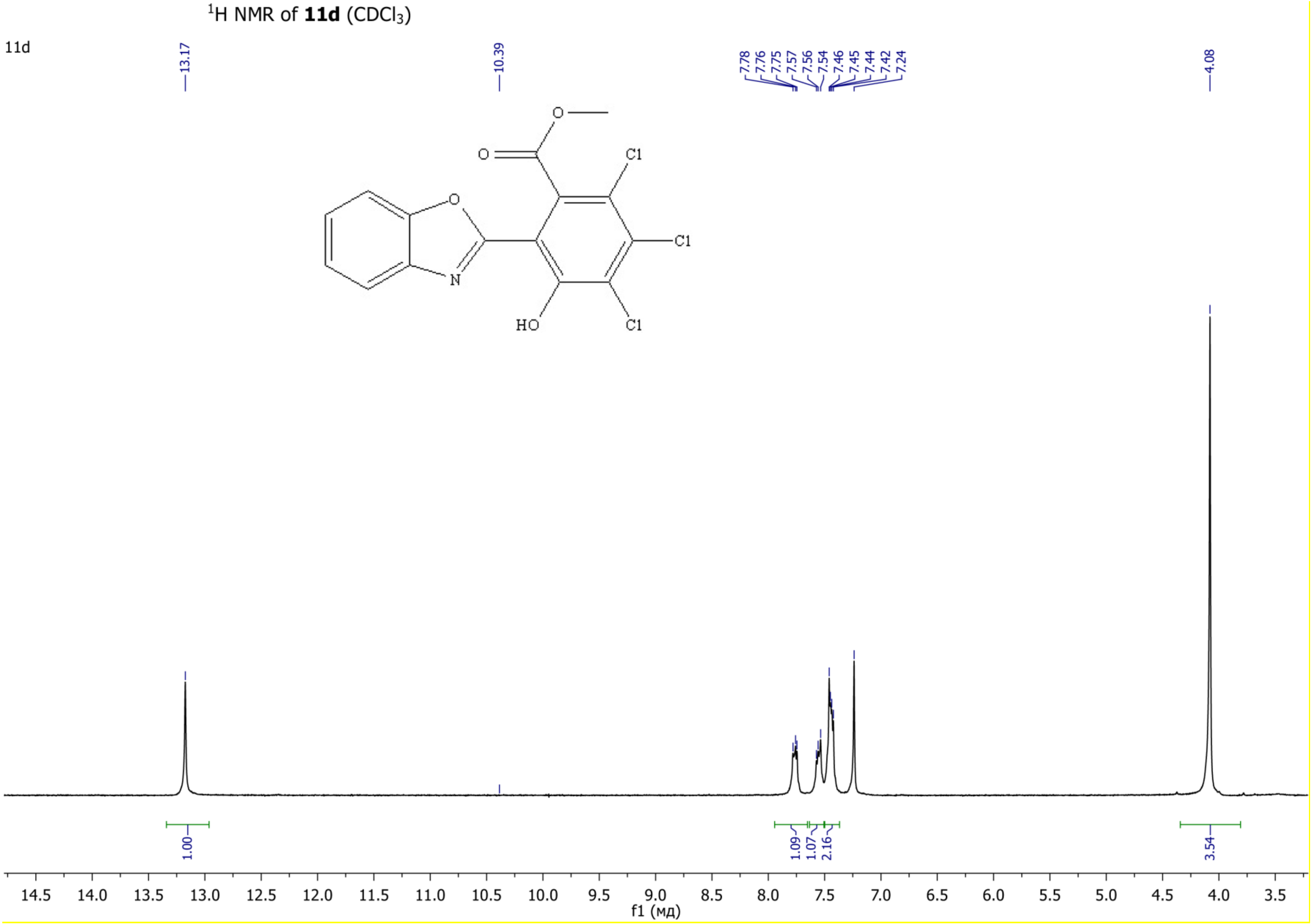

^{1}H NMR of **11d** ($CDCl_3$)
11d
13.17
10.39
7.78
7.76
7.75
7.57
7.56
7.54
7.46
7.45
7.44
7.42
7.24
4.08
O
O
Cl
O
Cl
N
HO
Cl
1.00
1.09
1.07
2.16
3.54
14.5
14.0
13.5
13.0
12.5
12.0
11.5
11.0
10.5
10.0
9.5
9.0
8.5
8.0
7.5
7.0
6.5
6.0
5.5
5.0
4.5
4.0
3.5
f1 (мд)

^{13}C NMR of **11d** ($CDCl_3$)

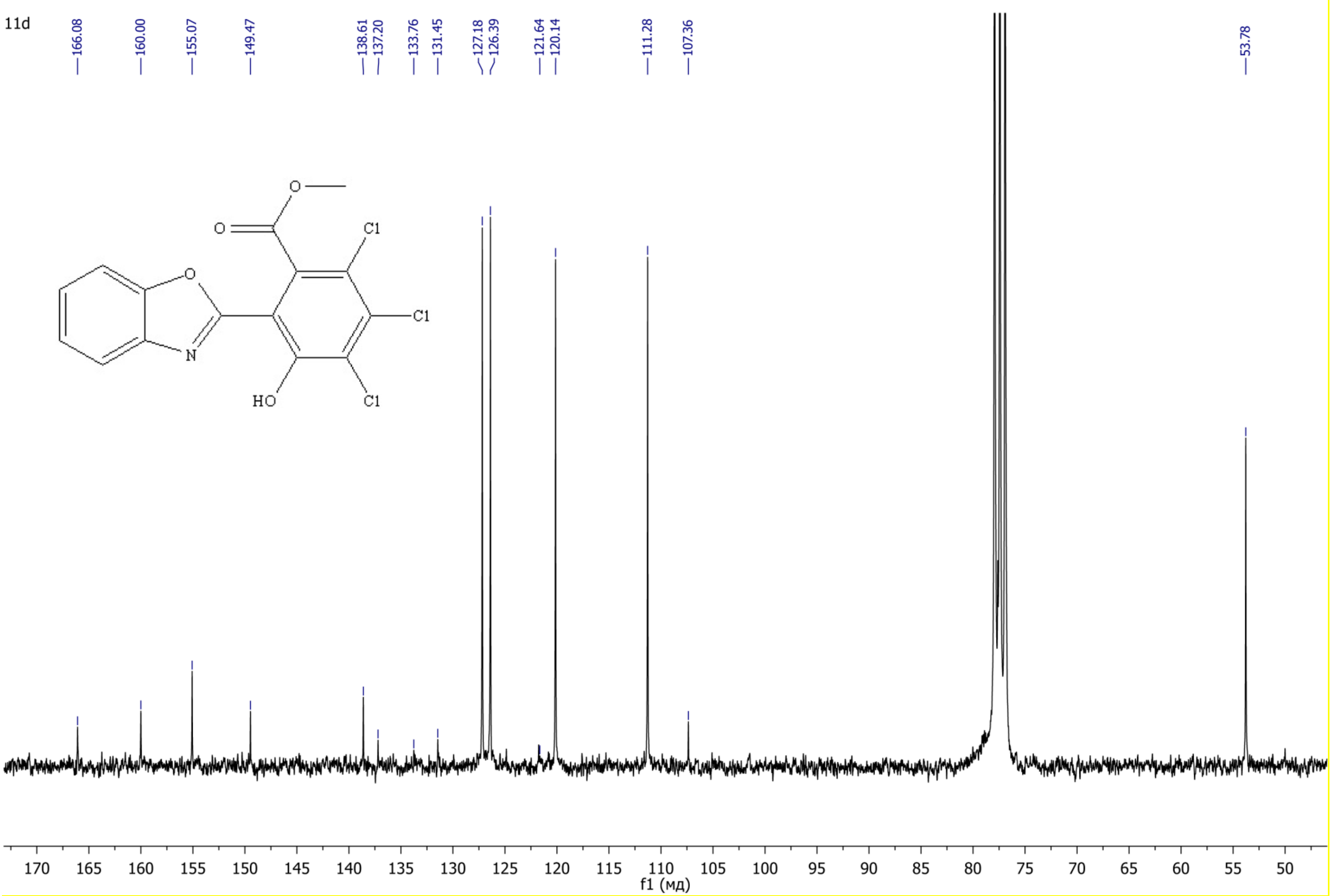

^{1}H NMR of **11e** ($CDCl_3$)

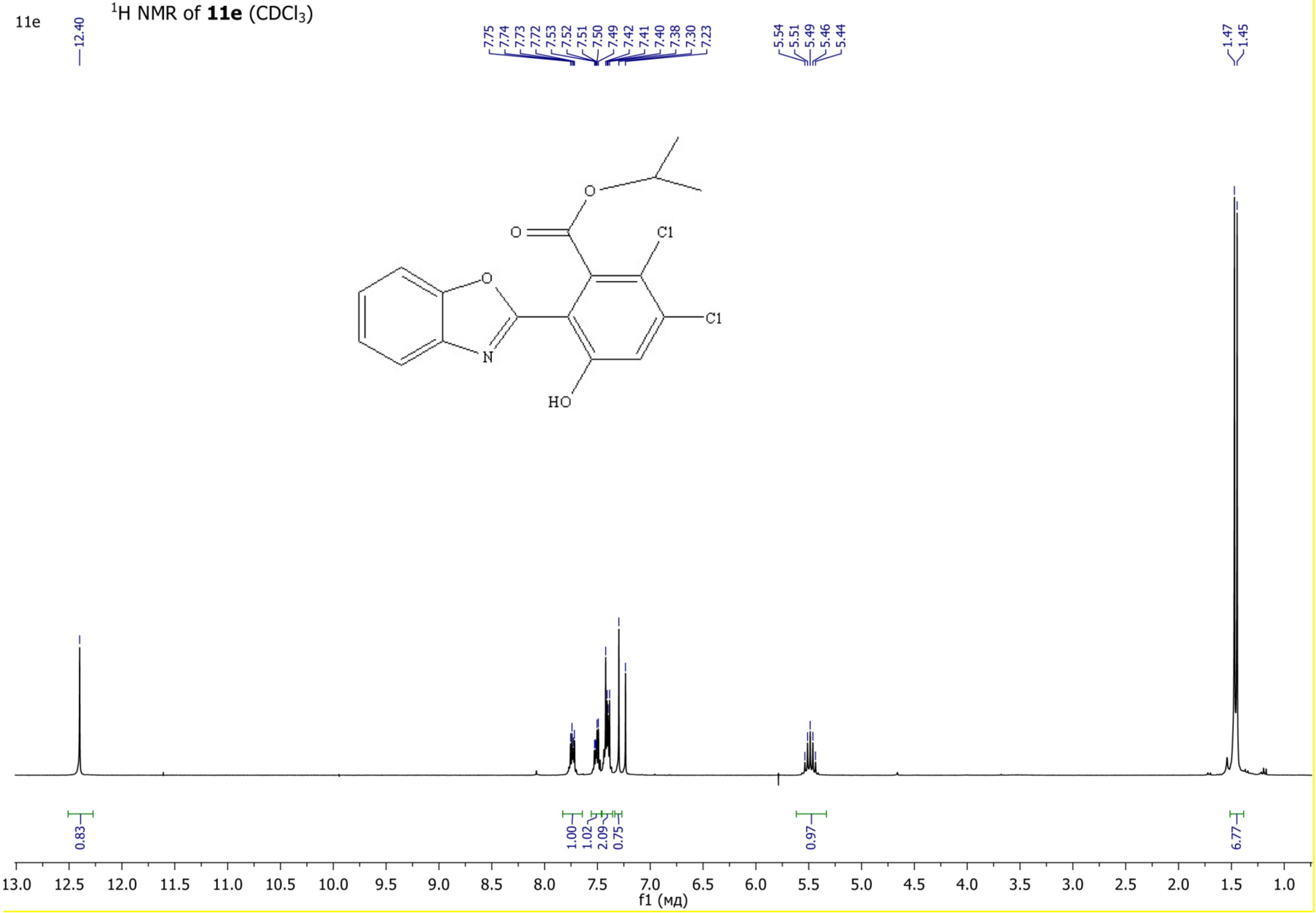

^{13}C NMR of **11e** ($CDCl_3$)

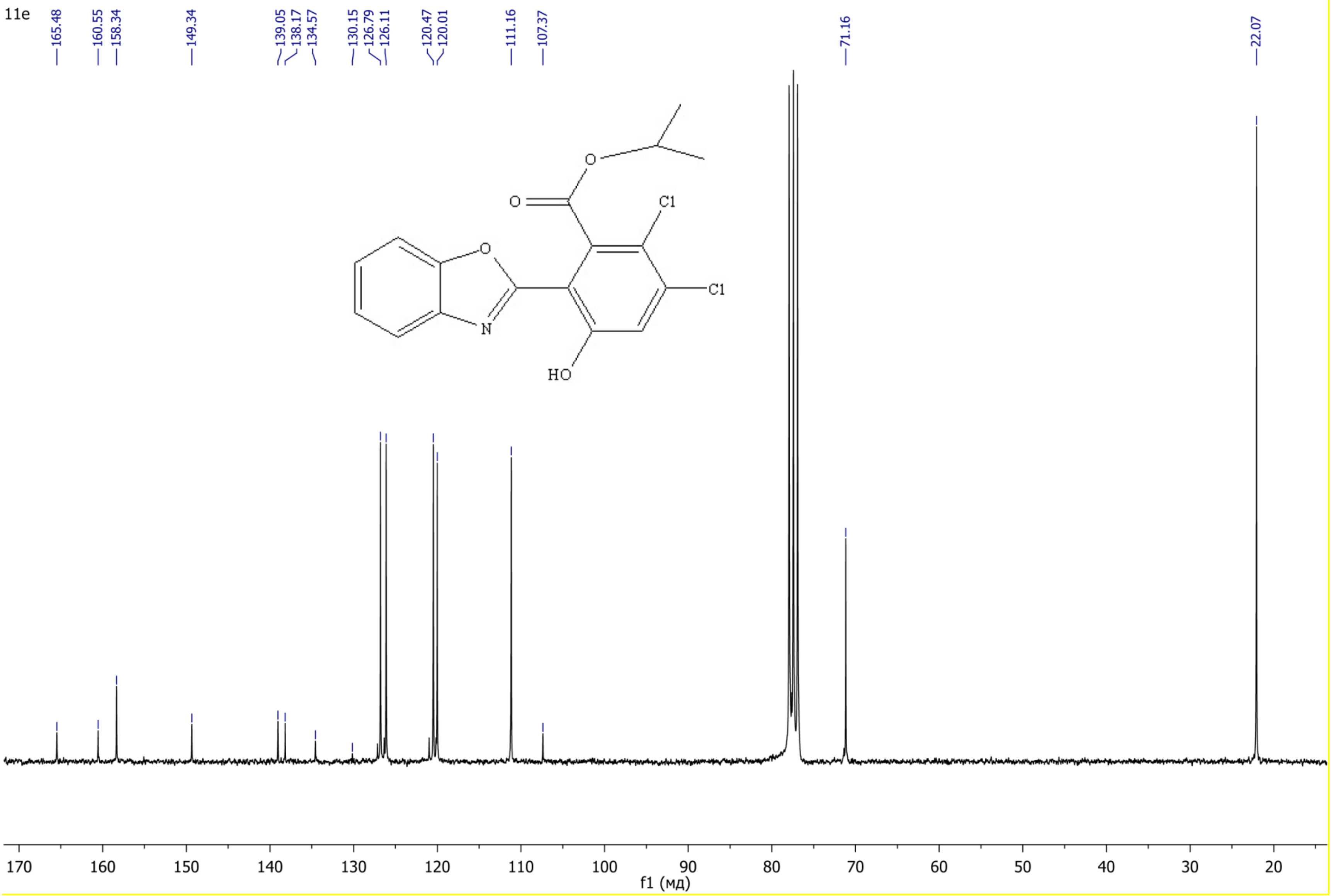

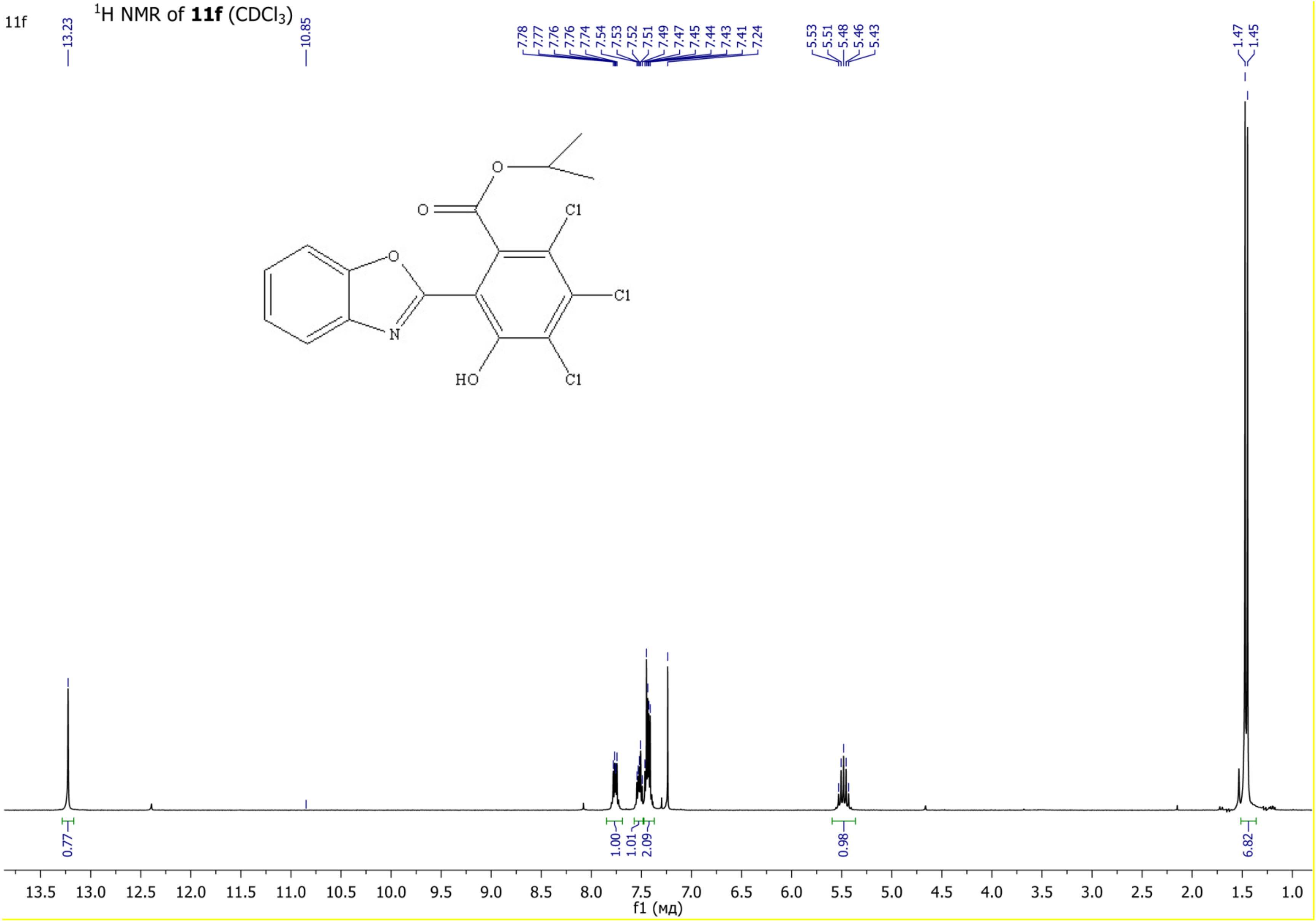
11f
^{1}H NMR of **11f** ($CDCl_3$)
13.23
10.85
7.78 7.77 7.76 7.76 7.74 7.54 7.53 7.52 7.51 7.49 7.47 7.45 7.44 7.43 7.41 7.24
5.53 5.51 5.48 5.46 5.43
1.47 1.45
O
O
Cl
Cl
Cl
HO
N
O
0.77
1.00
1.01
2.09
0.98
6.82
13.5 13.0 12.5 12.0 11.5 11.0 10.5 10.0 9.5 9.0 8.5 8.0 7.5 7.0 6.5 6.0 5.5 5.0 4.5 4.0 3.5 3.0 2.5 2.0 1.5 1.0
f1 (мд)

^{13}C NMR of **11f** ($CDCl_3$)

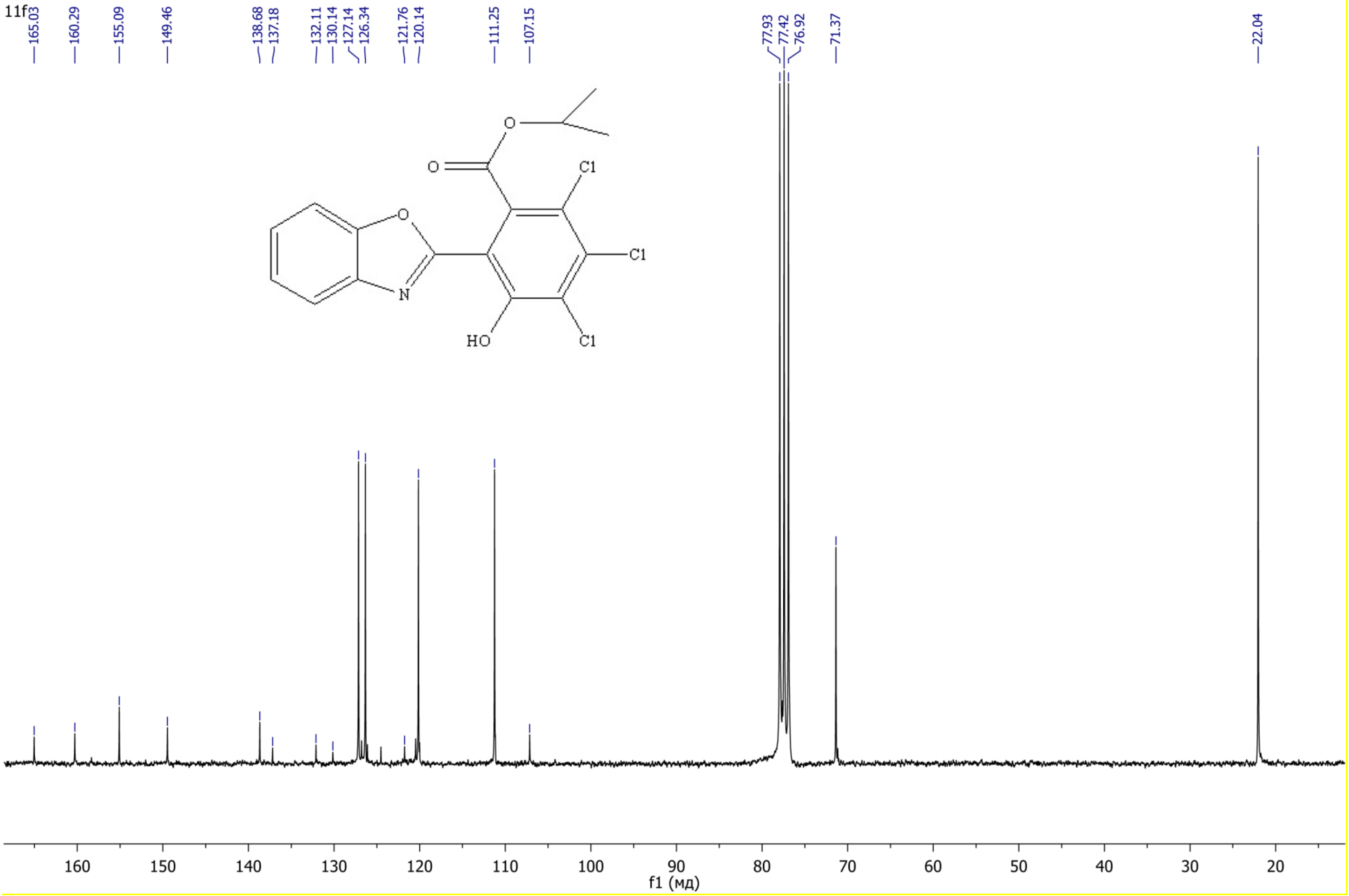

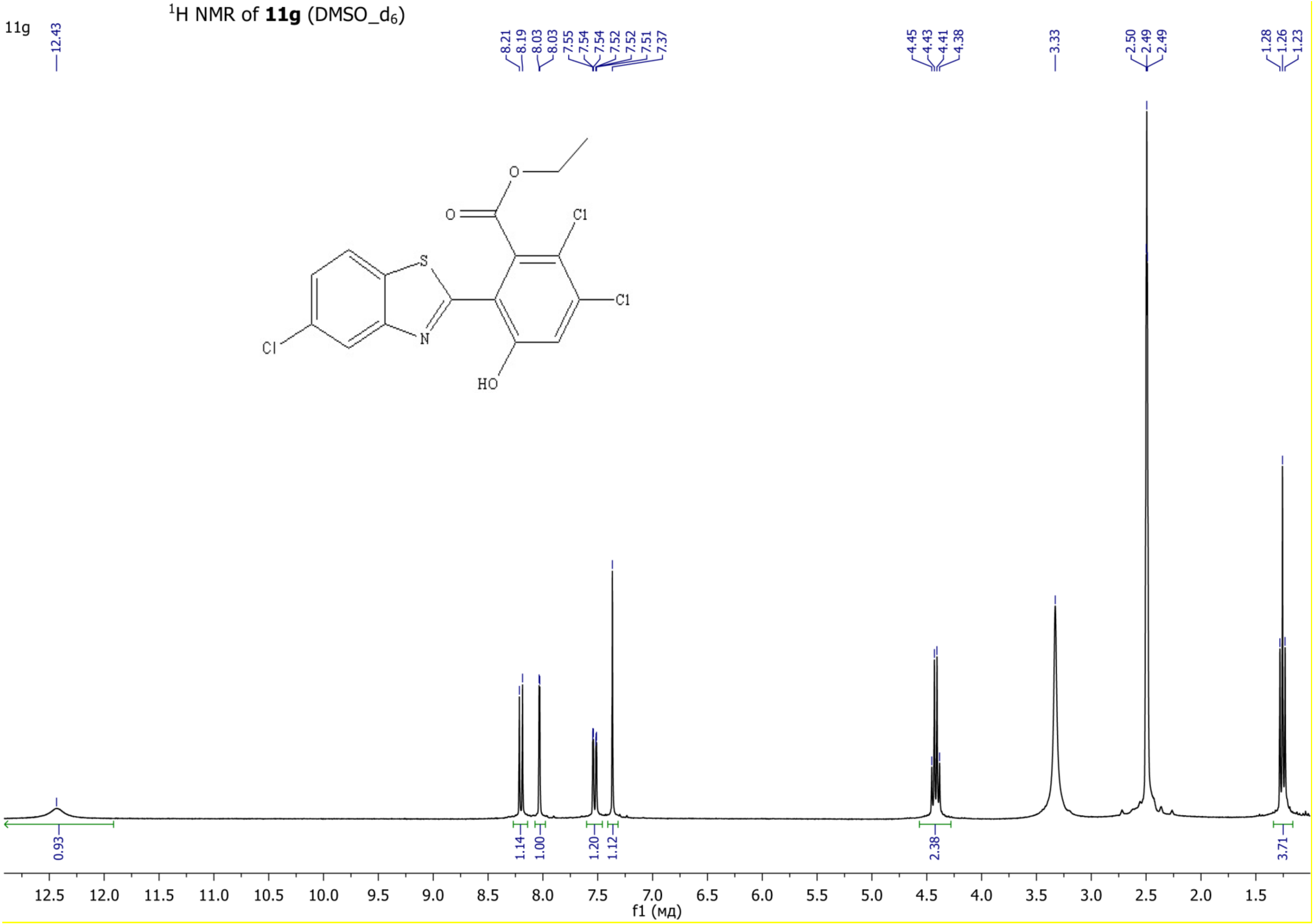
11g
^{1}H NMR of **11g** (DMSO_d$_6$)
12.43
8.21
8.19
8.03
8.03
7.55
7.54
7.54
7.52
7.52
7.51
7.37
4.45
4.43
4.41
4.38
3.33
2.50
2.49
2.49
1.28
1.26
1.23
O
O
Cl
Cl
S
N
Cl
HO
0.93
1.14
1.00
1.20
1.12
2.38
3.71
12.5
12.0
11.5
11.0
10.5
10.0
9.5
9.0
8.5
8.0
7.5
7.0
6.5
6.0
5.5
5.0
4.5
4.0
3.5
3.0
2.5
2.0
1.5
f1 (мд)

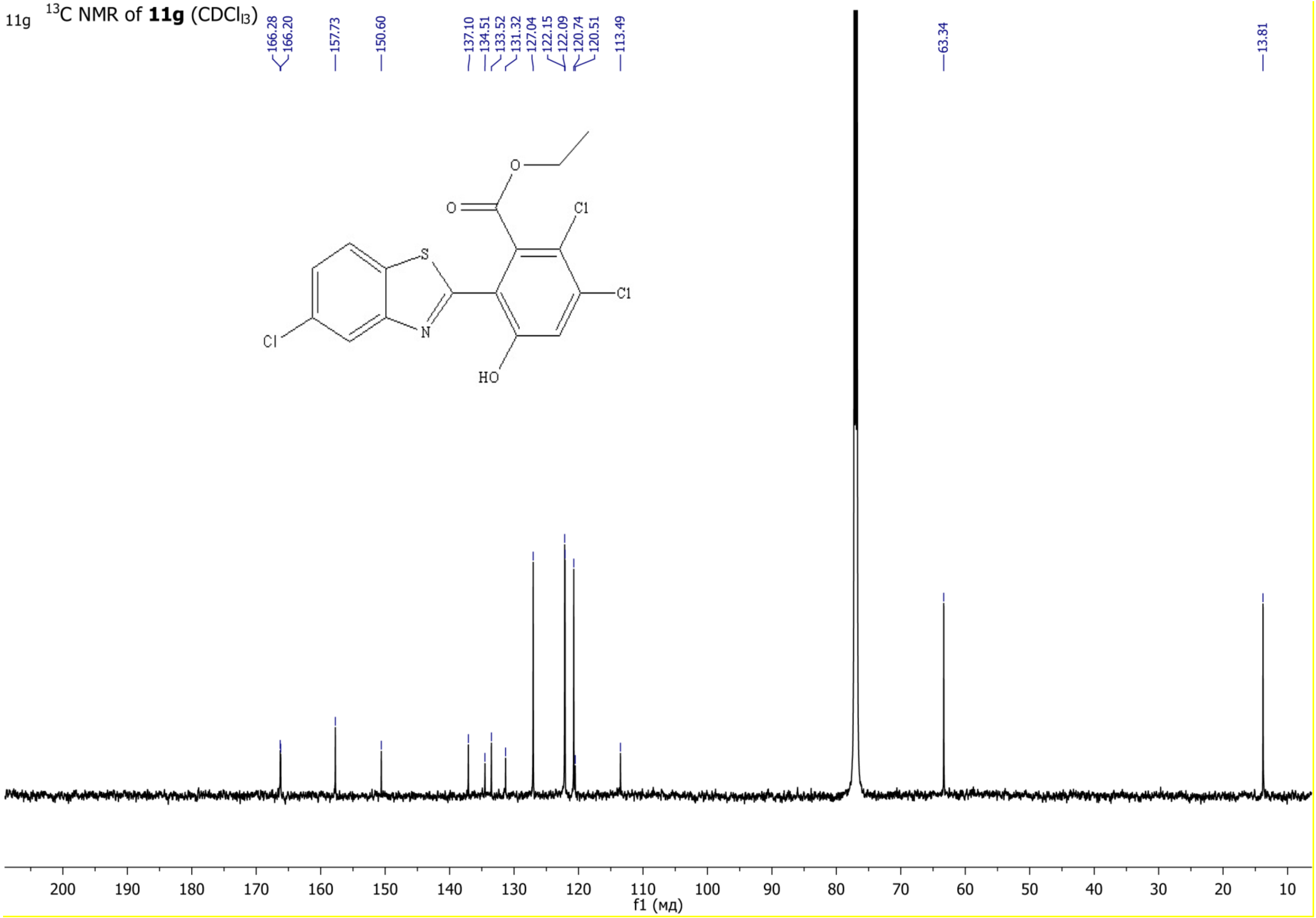
11g
^{13}C NMR of **11g** ($CDCl_3$)
166.28
166.20
157.73
150.60
137.10
134.51
133.52
131.32
127.04
122.15
122.09
120.74
120.51
113.49
63.34
13.81
O
O
Cl
Cl
S
N
Cl
HO
200 190 180 170 160 150 140 130 120 110 100 90 80 70 60 50 40 30 20 10
f1 (мд)

13

^{1}H NMR of **13** ($CDCl_3$)

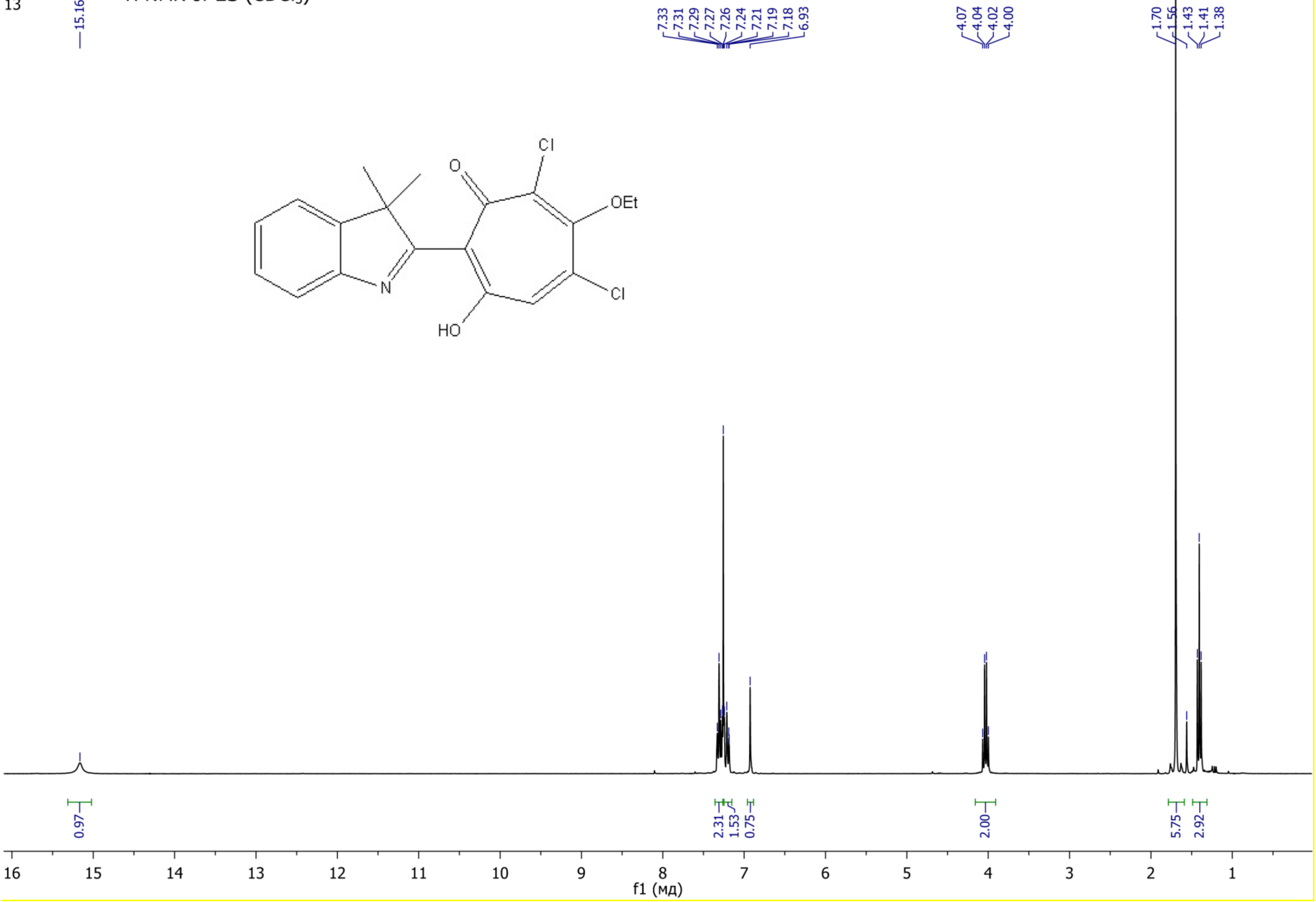

^{13}C NMR of **13** ($DMSO\_d_6$)

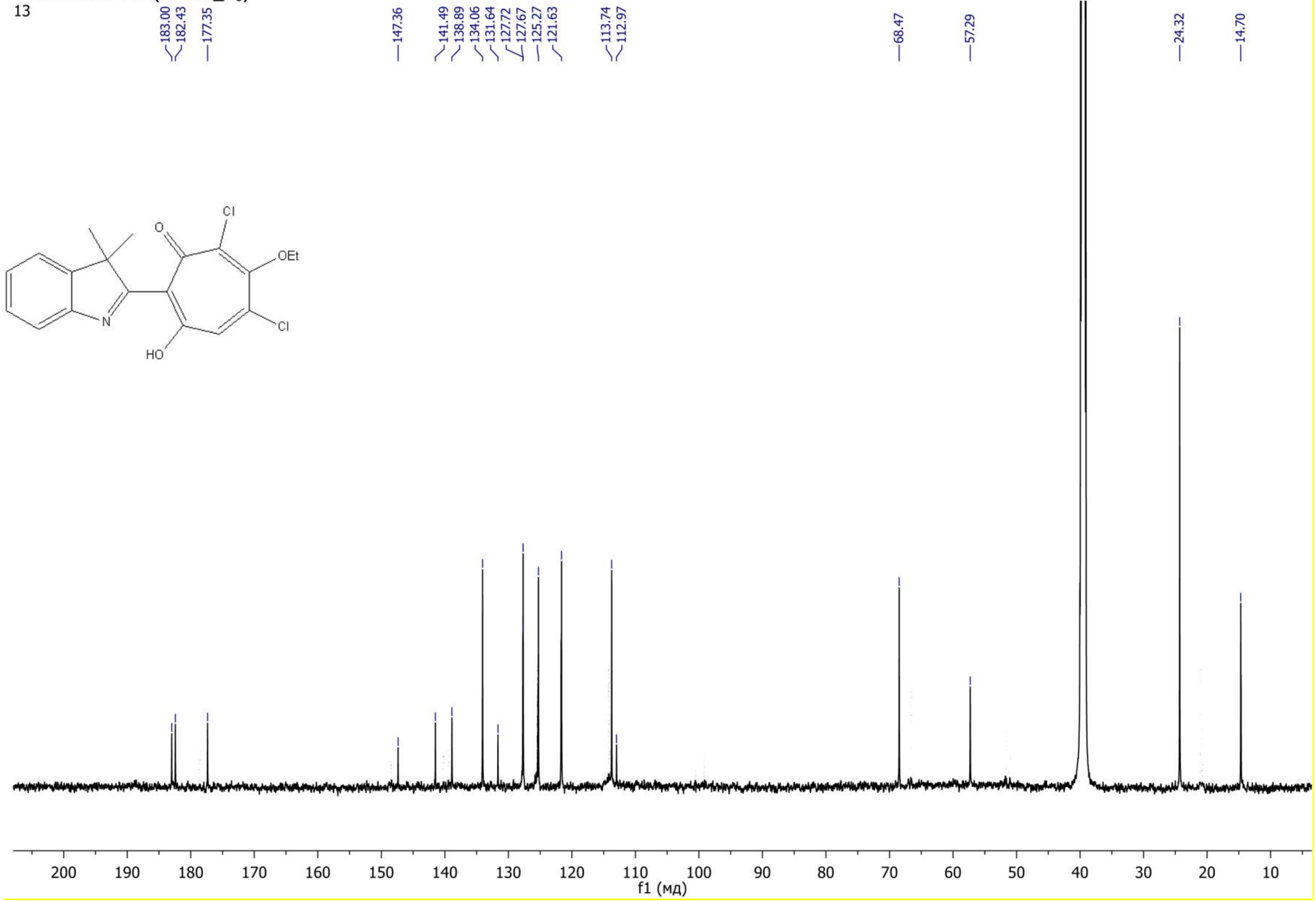

Supplement: File 2 — Copies of 1H and 13C NMR spectra of 5a-g, 6a-g, 11a-g, 13. [file Beilstein_J_Org_Chem-11-2179-s002.pdf]
